# Assessment of the Use of Patient Vital Sign Data for Preventing Misidentification and Medical Errors

**DOI:** 10.3390/healthcare10122440

**Published:** 2022-12-02

**Authors:** Jared Maul, Jeremy Straub

**Affiliations:** Department of Computer Science, North Dakota State University, Fargo, ND 58102, USA

**Keywords:** patient identification, artificial intelligence, vital sign data, medical error prevention, gradient descent trained expert system

## Abstract

Patient misidentification is a preventable issue that contributes to medical errors. When patients are confused with each other, they can be given the wrong medication or unneeded surgeries. Unconscious, juvenile, and mentally impaired patients represent particular areas of concern, due to their potential inability to confirm their identity or the possibility that they may inadvertently respond to an incorrect patient name (in the case of juveniles and the mentally impaired). This paper evaluates the use of patient vital sign data, within an enabling artificial intelligence (AI) framework, for the purposes of patient identification. The AI technique utilized is both explainable (meaning that its decision-making process is human understandable) and defensible (meaning that its decision-making pathways cannot be altered, just optimized). It is used to identify patients based on standard vital sign data. Analysis is presented on the efficacy of doing this, for the purposes of catching misidentification and preventing error.

## 1. Introduction

Medical mistakes are a significant problem. They range from systematic failures to isolated accidents and provider issues. A study, in 2013, estimated that medical errors cost over USD 20 billion each year and result in the death of 100,000 people [1].

Patient misidentification is a component of this issue and can result when providers lose track of which locations patients are assigned to, swap charts, or otherwise confuse one patient with another. Notably, a 2016 study found that the problem actually starts in patient registration [2], with patient misidentification at registration being the leading cause of misidentification, generally. Each hospital, on average, looses USD 17.4 million per year due to misidentification-attributable denied claims, and 86% of providers “have witnessed or have known of” a misidentification-attributable medical error [2].

This problem is considered to be “wicked” meaning that it is “complex and without a clear solution” and “difficult to solve” [3]. Despite procedures requiring providers to verify patients’ names, dates of birth, and ID bands, time pressures, ID band issues, and chaoticness can still result in issues [3].

This paper proposes the use of an artificial intelligence-based solution, which utilizes patient vital sign data, for the purposes of patient identification. It assesses whether, using this approach, patients can be readily identified to a level that would be useful for preventing misidentification. It also characterizes the level of misidentification prevention accuracy that is possible using the proposed approach.

The paper continues in Section 2, with a review of prior work which provides a foundation for the work presented herein. Next, in Section 3, the system design for the test system is presented. Section 4 presents the data that has been collected and the analysis of it. Finally, the paper concludes in Section 5 and discusses areas of potential future work.

## 2. Background

This section reviews prior work in three areas which provide a foundation for the current study. First, the issues caused by, and consequences of patient misidentification are discussed. Second, prior work on artificial intelligence, expert systems and neural networks is reviewed. Finally, the gradient descent trained expert system (GDES) technique is presented.

### 2.1. Patient Misidentification and Its Consequences

Patient misidentification has been labeled a “wicked” problem by Ferguson et al. [3]. It drains staff time, costing “clinicians” approximately 30 min during each shift searching for records, and costs hospitals millions of dollars in lost revenue [3]. If patients are given the wrong procedure or medication, it can result in injury or even patient death. A study [4] at the Veterans Health Administration, for example, found that, in 31% of reported misidentification incidents, a procedure was performed on the wrong patient. In other cases, misidentification can result in a failure to diagnose a condition or inform the patient of the diagnosis, delaying treatment [5]. The impact of misidentification can even extend beyond the death of the patient, as it can impair the process of relatives claiming or moving a deceased person’s corpse [6].

O’Neil et al. [7] also note the potential legal risks to providers and facilities from misidentification, which include liability under the torts of battery, false imprisonment, and emotional distress. In their study, they found that wristbands were missing in 70% of cases, nearly 8% of cases had illegible wristbands, and over 20% of cases had missing, erroneous, or conflicting ID information. In only 1% of cases, though, the wristband was actually on the wrong patient [7].

Ortiz and Amatucci [8] found that hurried staff were partially responsible for 64% of misidentifications and that an identification policy was not followed in nearly 50% of incidents. Language issues (46%), missing ID bands (38%), patients answering to incorrect names (38%), staff carelessness (35%) and using yes/no questions for identification (33%) were also large contributors.

In laboratory medicine, Dunn and Moga [9] identified patient identification as being responsible for “182 of 253 adverse events” and identified causes including admissions identification issues, mislabeling specimens, not using two sources of patient identification, and not using two person verification as being responsible. These incidents resulted in issues with cancer diagnoses, blood transfusions to incorrect patients (including incidents which resulted in incompatible blood being transfused) and information being placed in incorrect patient records.

In neonatal intensive care units, Gray et al. [10] found significant potential for misinformation. They found that approximately 50% of patients were at risk for potential misidentification due to similarities with their medical record numbers, name similarities, and common surnames.

Registration issues were examined by Bittle and Charache [11], who found that they occurred between seven and fifteen times each month due to issues with IT systems, training issues and not having a single index of patients. Levin, Levin and Docimo [12] examined the impact of computerized physician order entry on medical errors, noting that orders on misidentified patients occurred just under 1% of the time (across all orders) and had correlations with provider fatigue factors. Other studies found name similarity issues [13] and missing wrist bands [14] to be leading causes of misidentification.

A variety of solutions to this problem have been proposed ranging from procedural [7] and wrist band information [7] changes to bedside checks [15], real-time audits [16], training changes [17], and increasing “communication and collaboration” between providers and laboratory staff [18]. Technological solutions such as improved wristband technologies [19], the use of neural network image processing to identify patient sex to identify and reduce errors [20], using RFID and wireless technologies [21] and “proximity-based medical record retrieval” [22] have been proposed.

### 2.2. Artificial Intelligence, Expert Systems, and Neural Networks

AI is used for numerous applications including screening applicants for loans [23], scanning social media posts [24], and aiding people with psychological [25] and medical [26] needs. Some AI systems are able to improve their performance through unsupervised [27], semi-supervised [28], and supervised [29] learning processes. 

One of the most common forms of supervised learning is gradient descent [30], where corrections are applied iteratively to produce outputs closer to a goal. Backpropagation [31], which alters a network’s weightings working from the end of the network toward the beginning, is one type of gradient descent.

Expert systems are another AI technique, which are loaded with decision pathways from a human expert and are not typically used with machine learning. They were introduced in the 1960s [32,33] and 1970s [33] and, in their classical form, include a network of rules and facts and an inference engine [34]. Adaptations featuring optimization [35], neural networks [36], fuzzy set concepts [37], and fuzzy logic [38] have been developed.

### 2.3. Gradient Descent Trained Expert Systems

Arrieta et al. [39] proposed that there are two types of explainable artificial intelligence (XAI) systems: those that are inherently understandable and those that have been retrofitted to provide human understandability. A rule-fact expert system-based approach to machine learning [40] is inherently understandable. The system proposed in [40] goes beyond this standard, though, as it cannot create new associations: its learning process optimizes the weightings of existing associations. This prevents learning invalid associations, such as those that are responsible for AI system bias.

**Figure 1:**
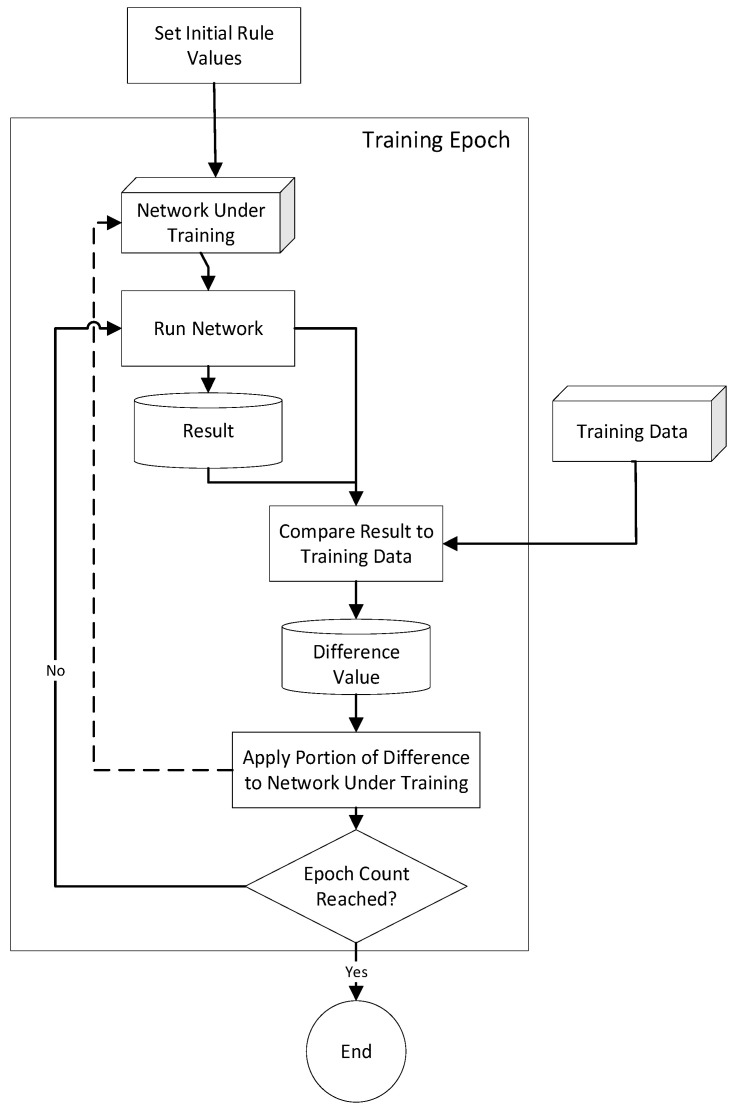
Gradient descent expert system training algorithm [45].

This system uses a gradient descent-style backpropagation learning algorithm on the expert system’s rule-fact network [40]. The rule-fact network is constructed by a human system developer, based on their understanding of the phenomena being modeled, and can be refined through performance testing, as needed. The backpropagation algorithm changes the weightings of rules’ inputs to better match the phenomena and reduce error, during the training process. To do this, a fraction of the error (between the current system output and target value for a training run) is proportionally allocated to each rule, based on its contribution to the output value. The algorithm for system training is depicted in Figure 1.

Several techniques have been proposed to enhance the system, including techniques designed to reduce error [41], change the way training is performed [42], and automate network development [43]. Its use has also been previously demonstrated for a limited number of applications (see, e.g., [44]).

## 3. System Design

To effectively use vital sign data for patient misidentification prevention, the vital sign data must be available to be correlated with patient identity information. Two scenarios for use are illustrative of how this could be achieved (though others are also possible). These scenarios are presented in Section 3.1. Then, in Section 3.2, system operations are described. Finally, limitations are discussed in Section 3.3.

### 3.1. System Use Scenarios

The first scenario is a smart hospital bed which would contain or be connected to the vital sign monitoring equipment and also know the supposed identity of the patient occupying it. Under this scenario, if the bed were to detect an anomaly it could notify hospital staff, display an alert and annotate any patient identity information with a misidentification concern. Notably, in addition to detecting possible patient misidentification, the same techniques proposed herein could be useful for detecting changes in medical condition; thus, any change would need to be investigated to confirm both patient identity and condition.

The second scenario is a patient record-keeping system. This system would be conceptually similar to the smart bed; however, it would not require specialized bed hardware. Instead, the system would look for indications that new incorrect information is being loaded for a patient, through the loading of vital signs data associated with the patient. If this data indicated a potential problem, patient misidentification would be suspected, and other contemporaneously loaded information would be flagged with the concern. Additionally, any access to the record for performing a procedure or dispensing a medication would trigger a misidentification concern alert to the provider.

Under both scenarios, the system would start with a presumed patient identity and historical vital sign data for the patient. It would collect or have vital sign data provided to it. The historical and current vital sign data, along with the presumed identity, would be provided to the misidentification system. This system would assess the likelihood of misidentification, using the process described subsequently and, if needed, provides a patient misidentification alert. 

If no prior data are available for the patient, the system would not be able to provide misidentification warnings until data has been collected for a period of time. Thus, when prior data are unavailable, or where it has been invalidated by a change in a patient’s medical condition (i.e., where an alert has been generated, the patient identity reverified and the system instructed that the identification is correct), the system would enter an initial data collection mode. While in this mode, it could caution users that it is learning the patient and not yet able to provide misidentification alerts, thus encouraging providers to be particularly careful. This could be augmented with provider practices that include additional verification, while the system is in this mode, to ensure that the patient is correctly verified manually (and, thus, also assuring that the data that is being collected will be associated with the correct individual).

**Figure 2:**
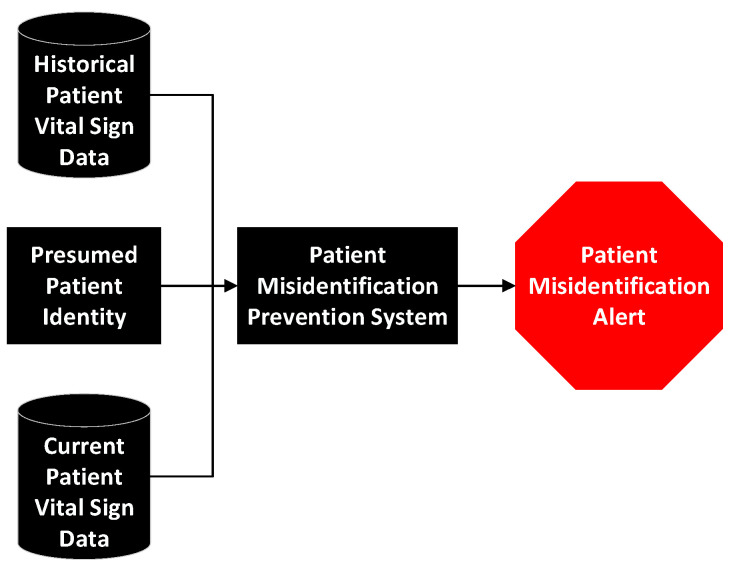
Patient misidentification prevention system diagram.

The basic operations of the system are depicted in Figure 2.

### 3.2. System Operations

A key decision, for system implementation, is what data to analyze. Ideally, the system would operate using vital signs which are easily and already commonly recorded. This would facilitate the use of sensors that would already be needed for other purposes also being used for this application. This would reduce costs and collection burden on the patient. From the variety of vital sign information available, the following four pieces of information were selected for use: heart rate, end-tidal carbon dioxide, respiratory rate, and blood pressure. These four vital signs were selected primarily because of availability. Each patient within the dataset has an abundance of data for each of these vital signs and these data were available for all patients. A key area of future work will be to identify whether other vital signs perform as well as, outperform, or underperform the ones analyzed in herein. In addition to assessing performance generally, the correlation of vital sign performance with demographic characteristics also merits assessment in future work.

Each piece of vital sign information’s numeric value is converted into a value between 0 and 1, to supply to the system for training. The data set contains a set of patients with vital sign data collected during surgery. Ten of these patients are used in this experiment. Each of these ten patients was selected because their procedures lasted longer than 70 min. Data used for training the system for a given patient were taken from times 30:00.00 through 39:59.99 of the patient’s procedure. This ten-minute period allows for 60,000 entries for each of the four vital signs.

The system was tested using a series of trials. Trial numbers are common across the datasets that were used for analysis and refer to a specific set of steps that were used for data processing and analysis, in all instances.

For example, trial 1 is performed by supplying the network with training data from patient one to produce a single output value. This output value also lies between 0 and 1. Following this, a data sample is taken from each of the ten patients at intervals within times 1:00:00.00 and 1:09:59.99. These samples are supplied to the network and their output values are compared with the original value generated by training the network at the beginning of the trial. For trial 1, this means that patient one will have ten minutes of data used to train the network and produce an output value. Following this each of the ten patients will have a small amount of data supplied to the network, producing ten output values, each corresponding to one of the ten patients. Ideally, the value generated by the sample from patient one will be most similar to the original value produced by training the network. Meanwhile, the other nine patients should produce output that differs from the original value. For trials 1 through 10, patients one through ten are each used to train the network for their respective trials. This convention is followed for trials 11 through 50, with each collection of ten trials being modified slightly as described below. Each of these 50 trials is performed three times, in order to test three different networks. Each of these three networks is run four times to explore four slightly different approaches, which is also described below.

For the experimentation presented herein, converting the raw data to the value between 0 and 1 was completed using methods that vary by trial. In trials 1 through 10 and 21 through 50, the method used was to take the value and divide it by the maximum value found in any of the patients. For example, at 30:00.00 patient one has a heart rate of 53 bpm. This was divided by the maximum heart rate of all patients, in this case 135 bpm, resulting in a value of 000.393. This approach is referred to as the comprehensive data conversion method. Trials 11 through 20 divide the particular value by the greatest value found in a given patient’s own data. This is referred to as the isolated data conversion method.

Both of these approaches could be used by a system operating in the real world and would simply use the largest value recorded to date; however, this would potentially necessitate caping values at 1.000, should a higher value be detected during operations, or retraining the network. In any case, for a real-world implementation, the divisor value would need to be set, potentially based on an in-situ pilot study building upon the experimentation, which is presented herein, so as to be consistent throughout operations. Notably, the largest value of all patients also approximates using the largest reasonable value, which would not change over time.

Once the values are converted, they are supplied to the system. Procedurally, this is completed using a set fact (SF) command (see [45]). A 32-character globally unique identifier (GUID) is assigned to each fact and rule which is used for identifying nodes within the network when issuing commands. For example, the command below sets the heart rate input fact to 000.393:

SF:{24da3290-e934-4a9c-84e9-6a0d856e5073}=000.393

Set fact commands are issued for each of the variables used in the given tests. Once the SF commands are issued, a training (TR) command is then issued. Training commands begin with a reference to a starting fact. The blood pressure fact is used as the starting fact and the second GUID included in the TR command is that of the output fact. 

Note that one of the SF commands is vestigial and not strictly necessary, as the initial fact included in a TR command is set by the TR command; however, all SF commands were issued for simplicity, as this approach allows the TR command issued to be changed without requiring changing the block of SF commands (and the issuance of an additional SF does not materially impact system operations). An extended discussion of this can be found in Appendix A along with technical details regarding the system commands.

The four input values (and the starting fact value from the TR command) are drawn from the data row currently being used for training. For the output value, 000.500, which is the midpoint of the valid output range, is used in all cases. This approach makes the trained patient the middle output value with other patients able to show a positive or negative deviation from this patient.

The command set (which is presented in Appendix A) of four SF commands followed by one TR command, is repeated for each row of data in the patient spreadsheets (60,000 records). Following these commands, a present (PR) command is issued.

Two forms of the PR command were utilized. For some tests, a set value (e.g., 000.500) was used that is at or near the middle of the allowable fact value range. The value of 000.500 was used in trials 1 through 20 of each set of data. In trials 21 through 30 the value of 000.600 was used, trials 31 through 40 used 000.700, and trials 41 through 50 used 000.550.

A second approach utilized, as input for the PR command, the blood pressure value from the last training row (so as to produce a natural output from the network). The form of this command was the same, except that the value assigned to blood pressure varied depending on the value in the final row.

A third approach was tested, which uses a fixed value (just like the first approach). The difference is that, just prior to the PR command being issued, all four facts are also set to that value.

Finally, a fourth and final approach is similar to the third, with one primary difference. The average value of each fact is calculated. These averages are used in a set of SF command and the PR command.

The results from these four different approaches to generating a baseline value for a given patient are compared herein. The output of the PR command is what all other results, which are based on performing PRs using data from later in the patient data sets, were compared against. This is referred to as the initial training output value or the target value.

### 3.3. Limitations

It is important to note that this system is intended for use as an additional layer of protection against patient misidentification. It is not designed to uniquely identify patients, nor is it designed to guarantee that patients will be identified correctly in all circumstances. Rather, it is designed to augment existing patient identification and misidentification prevention methods. It will provide partial support or refutation for the presumed patient identity provided. This analysis can occur in tandem with other commonly used patient identification methods, such as verifying identity details with the patient and, in this context, it is capable of reducing patient misidentification. Further expanding the identity assessment capabilities of the proposed system with the use of additional data elements and methods is a potential topic for future work.

## 4. Data and Analysis

The data used to test this system was sourced from patient monitoring and vital sign data that was recorded during surgical cases where patients underwent anesthesia at the Royal Adelaide Hospital (RAH) [46]. The data are typical of biometric information that is frequently collected during hospital stays.

Data were utilized from 10 patients: case01, case03, case04, case05, case06, case09, case11, case12, case13, and case14 in the RAH dataset [46]. These files correspond to patients one through ten, respectively, in the data presented in this section (e.g., patient one is associated with case01, patient two is associated with case03). Patients whose procedures were completed in less than 70 min (e.g., case02, case07, case08, and case10), and thus did not have a full set of data, were excluded. Training data were sourced from the fourth file for each patient (e.g., uq_vsd_case01_fulldata_04.csv for patient 1), which contains data collected from the times 30:00.00 to 39:59.99. For each patient, 60,000 rows of data are included covering this timeframe (however, in many cases, values are repeated so that only a handful of unique values are present). 

After the system was trained, data from later in patients’ surgery was used to test it. The results were generated by presenting data from the seventh file of each patient (e.g., uq_vsd_case01_fulldata_07.csv for patient 1), which covers the time from 1:00:00.00 to 1:09:59.99. For each patient, seven rows were presented for data collection: these were rows 3 (rows 1 and 2 contain header data), 10,000, 20,000, 30,000, 40,000, 50,000, and 60,000. Each of these rows was used to create a group of set fact commands (in the same way as was used for the training data) and a PR command was then run. After each run, all intermediate facts were reset (using the SF command) to the default value of 000.500 before performing the next run. Data from this experimental process, using three different network configuration designs, are presented in Section 4.1, Section 4.2 and Section 4.3.

### 4.1. First Network Design

**Figure 3:**
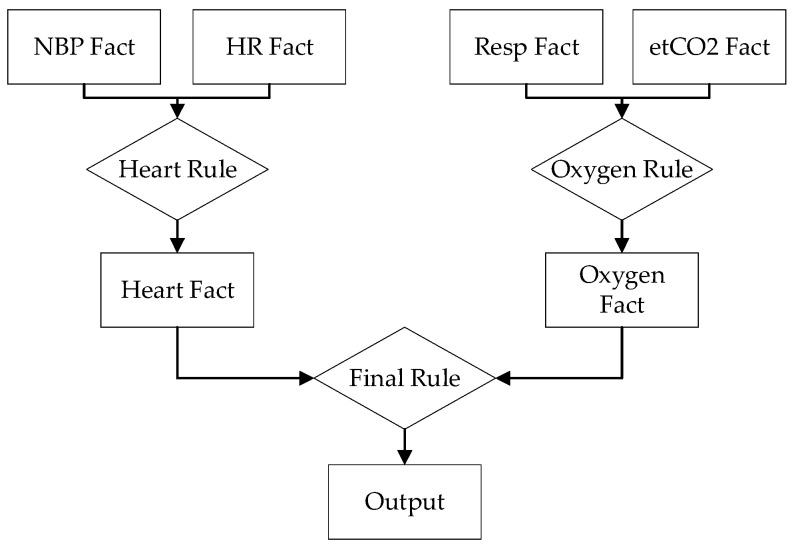
First GDES network.

The first network is comprised of seven facts and three rules. It uses a heart rule, which gets its value by combining blood pressure and heart rate data, as well as an oxygen rule, determined by combining respiratory rate and end-tidal carbon dioxide data. These two rules provide the heart fact and oxygen fact, respectively, which are inputs to the final rule and output fact. This model is presented in Figure 3.

The following table represents the results obtained using the first GDES network model, hence the header including Set 1. Trials 1 through 10 of Set 1 are presented in Table 1 and the first PR approach described in Section 3.2 is used. The average error column displays, for each trial, how much all patients tend to deviate from the initial output training fact on average. The avg err column focuses on this deviation in just the target patient, rather than the average of all patients. The lowest column displays a yes or no value, depending on whether the target patient had the lowest deviation from the initial training output of all patients. The correct at columns also have a yes or no value representing whether or not the target patient falls within a given margin of error. The false at column represents how many of the incorrect patients also fall within that margin of error. An example of how to read row one is as follows: In set 1, trial 1, the average deviation from the initial training output value across all ten patients is 0.037, while the average value of patient one only deviated by 0.012. This deviation, while lower than average, is not the lowest of all ten patients in trial 1; therefore, the lowest column displays N. Because the average deviation of patient one is greater than 0.01 the correct at 0.01 column also displays an N. However, the deviation is lower than 0.025 so the correct at 0.025 column (and the remaining correct at columns) display a Y value. In trial 1, no patients deviate from the initial output value by less than 0.01, as indicated in the False at 0.01 column. Five patients deviated by less than 0.025, seven patients deviated by less than 0.05, and eight patients deviated by less than 0.10.

From these results, a ratio of correct-to-incorrect patients can be determined for each margin of error by examining the collection of all ten rows. In these ten trials, there were two correct patients that fell within a 0.01 margin of error (these being trials 6 and 9) while the trials averaged 0.8 incorrect patients also falling within this margin (with 0.8 being the average of the false at 0.01 column). This ratio is 2:0.8, or 2.5. At a 0.025 margin of error, this ratio is 2.22. At a 0.05 error margin, the ratio is 1.66. Finally, at an error margin of 0.10, this ratio is 1.52. A high number of correct patients falling within a given error margin corresponding to a low number of incorrect patients also falling within that error margin means a high-performing model.

Each of the following tables highlight the highest and lowest performing collections of all trials. All remaining results are shown in Appendix B.

Table 2 displays the results from the lowest performing collection of trials that use the first GDES network model, with rows one through ten corresponding to trials 11 through 20, respectively. Because these are trials 11 through 20, the isolated data conversion method is used. The third PR approach described in Section 3.2 is what was utilized for these ten trials. As seen in the table, the target patient often deviates from the initial training output value more than the average patient, indicating these trials do not favor the correct patient at any margin of error. This is the case in trials 3, 4, 6, 7, 8, and 9. No patients, correct or incorrect, fall within any listed margins of error.

Table 3 displays results from the highest performing collection of trials using the first GDES model. It is trials 21 through 30, indicating that the comprehensive data conversion method is used and that the default fact value is 000.600. In all but one trial (trial 8), the target patient deviates from the target value less than the average patient. The highest ratio of correct-to-incorrect patients is found at an error margin of 0.01. At this margin, three correct patients are included with an average of 0.6 incorrect also being included. Thus, the ratio is 5. Notably, at an error margin of 0.025, there are 6 correct patients included with an average of 2.1 incorrect patients per trial. While this ratio is lower, at 2.86, there are twice as many correct patients included within the margin. No other trial collections produced multiple ratios this high utilizing the first GDES network model, although other models do have higher-performing trials.

### 4.2. Second Network Design

**Figure 4:**
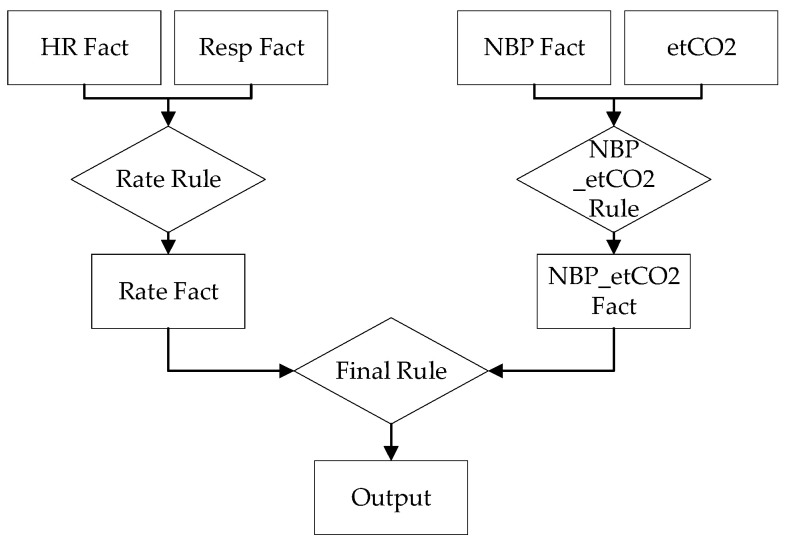
Second GDES network.

The second network design, shown in Figure 4, links heart rate and respiratory rate into a single rate rule while blood pressure and end-tidal carbon dioxide combine into another rule. These rules produce facts which serve as inputs to the final rule, which produces the output.

A similar set of tests were performed with this second network. Trials 1 through 10 utilized a default weight of 0.5 and the comprehensive data conversion method. Trials 11 through 20 used a default weight of 0.5 and the isolated data conversion method. Trials 21 through 50 utilized the comprehensive data conversion method with default weights of 0.6, 0.7, and 0.55 for trials 21 through 30, 31 through 40, and 41 through 50, respectively.

Table 4 displays the results of trials 1 through 10 utilizing the second GDES network model and the fourth PR approach, as described in Section 3.2. These trials are noteworthy as at an error margin of 0.01 there are four correct patients included with an average of 0.7 incorrect patients also included in the margin. This ratio, 4:0.7, or 5.71, is the second highest of any collection of trials. While this is noteworthy, the actual number of correct patients within the margin of error is only four, meaning that in most of the trials the correct patient did not fall within a 0.01 margin of error.

Table 5 represents the lowest performing collection of trials that utilize the second GDES network model. This is trials 11 through 20, indicating the use of the isolated data conversion method. The third PR approach described in Section 3.2 is used. In trials 11, 13, 14, 17, 18, and 19 the target patient deviated from the target value more than the average of all patients. No patients, correct or incorrect, fall within any listed margin of error.

Table 6 displays the highest performing trial collection utilizing the second GDES network model. It represents trials 21 through 30, indicating the default fact value used is 000.600. The second PR approach described in Section 3.2 is used. At a margin of error of 0.01 there are 3 correct patients included and an average of 0.6 incorrect per trial. This is a correct-to-incorrect ratio of 5. At a 0.025 margin of error 5 correct patients are included along with an average of 1.2 incorrect patients. This ratio is 4.17.

### 4.3. Third Network Design

**Figure 5:**
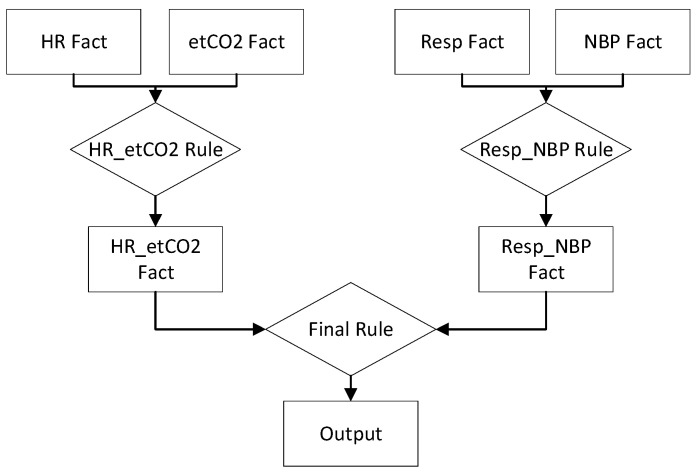
Third GDES network.

The third network design, which is depicted in Figure 5, combines the heart rate and end-tidal carbon dioxide facts with a single rule, while respiratory rate and blood pressure are combined using a second. These two rules produce facts that serve as inputs to the final rule, leading to the output fact. Notably, this network design tended to produce greater accuracy than the previous two networks, so results for this model will be explored in slightly more detail.

Table 7 is a noteworthy performer of the third GDES network model. It represents trials 1 through 10, so the default fact value used in this case is 000.500. It also uses the first PR approach described in Section 3.2. As can be concluded from the average error and avg err columns, output from the target patient is always closer to the target value than the average patient. The most notable aspect of these trials is found at the 0.025 margin of error. Across seven of the trials, the correct patient falls within the margin. Meanwhile, across the trials, an average of 1.7 incorrect patients also fall within the margin. This is a 7:1.7 correct-to-incorrect ratio, or 4.12. The high ratio, along with seven correct patients falling within the margin of error, makes this trial collection one of the most accurate.

Table 8 represents an exceptional collection of trials for several reasons. It is trials 11 through 20, indicating the isolated data conversion method is used. It also utilizes the second PR approach. Specifically, these trials perform well at a 0.01 margin of error. While a relatively unimpressive four trials have included patients that fall within that margin, there is an average of only 0.5 incorrect patients also being included. This 4:0.5 ratio, or 8, is the highest of any collection of trials across the entirety of this experiment. Additionally, this is unexpected considering the isolated data conversion method generally underperforms. While this is a noteworthy ratio, the actual number of correct patients falling within the margin is not substantial in comparison to some of the other high performers, particularly those utilizing GDES model three.

Table 9 displays the lowest performing trials of set 3. In trials 13, 14, 16, 17, 18, and 19 the target patient deviated further from the target value than most other patients. It uses the isolated data conversion method and the third PR approach. Like the corresponding lowest performers of the other GDES models, no patients, correct or incorrect, fall within any listed margin of error. The commonalities between all of the lowest performers for each model are the isolated data conversion method and the third PR approach. This indicates that these tend to produce low-performing trials, especially when used in tandem.

Table 10 displays the results of another high-performing trial collection of GDES model three. These are trials 21 through 30 and use the second PR approach. In all trials except 28, the target patient is closer to the target value than most other patients. At an error margin of 0.01, there are two correct patients that fall within the margin while an average of 0.4 incorrect patients are also included. This correct-to-incorrect patient ratio is then 5. Expanding to a 0.025 margin of error improves the results further. Five correct patients fall within the margin of error while an average of 0.9 incorrect patients are also included. This ratio is then 5.56. This ratio is the third highest correct-to-incorrect patient ratio across the entire experiment, and the highest ratio of any error margin that includes five or more incorrect patients.

Table 11 displays the results of trials 41 through 50 of the third GDES network model and first PR approach. Of all trials, these ten produce the most desirable outcome. At a margin of error of 0.025 nine of ten correct patients are included with an average of 1.9 incorrect patients also falling within the margin. This ratio, 9:1.9 or 4.74 is among the very highest and the error margin includes substantially more correct patients than any other trial collection with a comparable ratio.

Table 12 summarizes each of the high performers with a few key details in order to compare the variables present across trials. As can be seen, the third model tends to outperform the first and second. A default value of 000.600 tends to outperform the others, as well as using the actual PR value to determine the target value. Only error margins of 0.01 and 0.025 are present in the top performers. While some of these variables tend to outperform other trials on average, they are not strictly superior. For example, using the actual PR value tended to produce higher correct-to-incorrect patient ratios on average; however, using the single 0.5 PR value results in higher numbers of incorrect patients falling within a 0.025 margin of error. Ultimately, the last column in Table 12 displays the results of the top performer. That combination of model, default fact value, and PR approach demonstrated in this case the ability to eliminate 80% of incorrect patients on average per trial while only eliminating the target patient in one trial.

## 5. Conclusions and Future Work

This paper has presented and analyzed a prospective technology, which is designed to help medical providers recognize misidentified patients. Notably, this is not performed via specific patient identification but rather by providing support or refutation for presumed identifications. Thus, this system could be used to provide alerts indicating possible misidentification for human follow-up. It could also be potentially paired with other indicator subsystems as part of a multi-factor patient misidentification system.

To assess the efficacy of the proposed approach, several algorithms for identifying misidentified patients, using gradient descent expert systems, were evaluated in this work. These included three different network designs, multiple ways of preparing data to supply it to the network, and different ways of generating the target value for a given patient, based on their historic vital signs data.

There are several key outcomes from the analysis of the trial data presented herein. First, it was shown that the GDES network design itself clearly affects the efficacy of the system’s ability to identify misidentified patients. Despite each of the three network designs utilizing the same starting fact values and the same number of facts and rules, there were clear differences in performance between the different network designs. In the case where the target value was generated with a single 0.5 input, the third network design outperformed the other two. This indicated a potential advantage associated with linking patient heart rates with end-tidal carbon dioxide and respiratory rates with blood pressure.

Second, it was shown that the comprehensive data conversion method consistently outperformed the isolated data conversion method. This is evident from the fact that trials 11 through 20 for each network design tended to be the least accurate, and these were the batches of trials that utilized the isolated conversion method. 

The third area of analysis is the target value used for training. Of the values used, there was no clearly superior performer. Different networks performed better with different values.

Overall, the results obtained are quite promising. While none of the trials demonstrated the capability of positively identifying all patients by only their vital signs, many of the trials demonstrated a consistent ability to eliminate many—and in some cases—the majority of incorrect patients, allowing the system to provide an effective warning for many single patient mix-up scenarios. In particular, the best performers, listed in Table 12, show that the GDES system can effectively rule out incorrectly identified patients in the majority of cases.

There are several other factors, which were not explored in the present study, that are key topics for potential future work. One area is assessing the level of training data that is needed. In this regard, two key considerations exist. The first is to determine what level of training is most effective. To this end, future work can focus on assessing whether using all 60,000 data records, which were used for this study for training, provides the best results. The second area of consideration is to assess what the cost and benefit tradeoff of using less than the optimal amount of data is, as operating with lower amounts of data would allow the system to provide misidentification warnings with less input data and potentially learn about a patient more promptly, before a mix-up can occur.

Another area for potential future work is the development of additional networks and their assessment. These networks could use some or all of the input data used in this study and potentially augment it with additional data types. In particular, the data analyzed could be augmented with image data, which could potentially be collected using providers’ tablet computers, providing another independent source of patient validation/misidentification warning.

In conjunction with the above, a third area of potential future work is the assessment of the efficacy of using other types of vital sign data. This analysis could compare the ease and cost of collection, the amount of data required and the performance of the system, presenting a trade-off analysis that could guide real-world implementation decision-making.

Overall, this work has shown the efficacy of using GDES in a different way from previous work, where network result values are compared to each other instead of presenting multiple subjects to a single network for classification. Additionally, this work has shown the potential promise of using patient vital sign data for misidentification prevention. The data and analysis presented have demonstrated a meaningful ability to eliminate incorrect patients using common vital sign data. Based on this initial work, a number of promising areas for additional work have been identified for future exploration.

## Figures and Tables

**Table 1:** Set 1, trials 1–10 summary data (single 0.5 PR value).

Average Error	Avg Err	Lowest	Correct				False			
			At 0.01	At 0.025	At 0.05	At 0.1	At 0.01	At 0.025	At 0.05	At 0.10
0.037	0.012	N	N	Y	Y	Y	0	5	7	8
0.044	0.035	N	N	N	Y	Y	1	5	7	8
0.129	0.064	N	N	N	N	Y	0	0	0	2
0.064	0.010	N	N	Y	Y	Y	1	2	6	6
0.034	0.033	N	N	N	Y	Y	3	5	7	8
0.045	0.004	Y	Y	Y	Y	Y	0	2	6	8
0.059	0.010	N	N	Y	Y	Y	1	3	4	8
0.056	0.024	N	N	Y	Y	Y	0	2	5	8
0.050	0.002	Y	Y	Y	Y	Y	2	3	5	8
0.153	0.100	N	N	N	N	Y	0	0	1	2

**Table 2:** Set 1, trials 11–20 summary data (all 0.5 PR values).

Average Error	Avg Err	Lowest	Correct				False			
			At 0.01	At 0.025	At 0.05	At 0.1	At 0.01	At 0.025	At 0.05	At 0.10
0.199	0.165	N	N	N	N	N	0	0	0	0
0.204	0.193	N	N	N	N	N	0	0	0	0
0.204	0.243	N	N	N	N	N	0	0	0	0
0.204	0.218	N	N	N	N	N	0	0	0	0
0.204	0.168	N	N	N	N	N	0	0	0	0
0.204	0.222	N	N	N	N	N	0	0	0	0
0.204	0.216	N	N	N	N	N	0	0	0	0
0.204	0.226	N	N	N	N	N	0	0	0	0
0.204	0.208	N	N	N	N	N	0	0	0	0
0.204	0.111	Y	N	N	N	N	0	0	0	0

**Table 3:** Set 1, trials 21–30 summary data (actual PR value).

Average Error	Avg Err	Lowest	Correct				False			
			At 0.01	At 0.025	At 0.05	At 0.1	At 0.01	At 0.025	At 0.05	At 0.10
0.053	0.024	N	N	Y	Y	Y	1	3	4	8
0.053	0.035	N	N	N	Y	Y	0	4	6	8
0.055	0.002	Y	Y	Y	Y	Y	0	1	4	8
0.076	0.016	Y	N	Y	Y	Y	0	2	3	7
0.049	0.030	N	N	N	Y	Y	0	2	7	8
0.061	0.002	Y	Y	Y	Y	Y	0	1	3	7
0.068	0.010	N	N	Y	Y	Y	1	3	3	7
0.062	0.085	N	N	N	N	Y	2	2	3	7
0.065	0.008	N	Y	Y	Y	Y	2	3	3	7
0.297	0.173	N	N	N	N	N	0	0	0	1

**Table 4:** Set 2, trials 1–10 summary data (average actual PR values).

Average Error	Avg Err	Lowest	Correct				False			
			At 0.01	At 0.025	At 0.05	At 0.1	At 0.01	At 0.025	At 0.05	At 0.10
0.040	0.008	N	Y	Y	Y	Y	1	4	7	7
0.038	0.022	N	N	Y	Y	Y	2	5	7	8
0.161	0.037	Y	N	N	Y	Y	0	0	0	1
0.096	0.012	Y	N	Y	Y	Y	0	1	4	6
0.074	0.010	Y	Y	Y	Y	Y	0	0	4	7
0.069	0.036	N	N	N	Y	Y	0	3	3	7
0.053	0.061	N	N	N	N	Y	2	5	6	7
0.321	0.305	N	N	N	N	N	0	0	0	0
0.038	0.010	N	Y	Y	Y	Y	1	5	7	8
0.060	0.007	N	Y	Y	Y	Y	1	2	5	7

**Table 5:** Set 2, trials 11–20 data (all 0.5 PR values).

Average Error	Avg Err	Lowest	Correct				False			
			At 0.01	At 0.025	At 0.05	At 0.1	At 0.01	At 0.025	At 0.05	At 0.10
0.402	0.407	N	N	N	N	N	0	0	0	0
0.423	0.384	N	N	N	N	N	0	0	0	0
0.423	0.457	N	N	N	N	N	0	0	0	0
0.423	0.468	N	N	N	N	N	0	0	0	0
0.423	0.418	N	N	N	N	N	0	0	0	0
0.423	0.334	Y	N	N	N	N	0	0	0	0
0.423	0.466	N	N	N	N	N	0	0	0	0
0.423	0.476	N	N	N	N	N	0	0	0	0
0.423	0.458	N	N	N	N	N	0	0	0	0
0.423	0.361	N	N	N	N	N	0	0	0	0

**Table 6:** Set 2, trials 21–30 summary data (actual PR value).

Average Error	Avg Err	Lowest	Correct				False			
			At 0.01	At 0.025	At 0.05	At 0.1	At 0.01	At 0.025	At 0.05	At 0.10
0.050	0.006	N	Y	Y	Y	Y	2	3	4	8
0.072	0.055	N	N	N	N	Y	0	1	5	6
0.159	0.029	Y	N	N	Y	Y	0	0	0	1
0.055	0.011	N	N	Y	Y	Y	2	2	4	7
0.121	0.027	Y	N	N	Y	Y	0	0	1	3
0.053	0.009	N	Y	Y	Y	Y	1	1	4	8
0.071	0.040	N	N	N	Y	Y	0	1	3	7
0.206	0.115	N	N	N	N	N	0	0	0	0
0.042	0.002	N	Y	Y	Y	Y	1	4	6	8
0.124	0.021	Y	N	Y	Y	Y	0	0	0	2

**Table 7:** Set 3, trials 1–10 summary data (single 0.5 PR value).

Average Error	Avg Err	Lowest	Correct				False			
			At 0.01	At 0.025	At 0.05	At 0.1	At 0.01	At 0.025	At 0.05	At 0.10
0.056	0.005	Y	Y	Y	Y	Y	0	0	4	9
0.026	0.011	N	N	Y	Y	Y	3	4	7	9
0.133	0.096	N	N	N	N	Y	0	1	1	2
0.090	0.007	Y	Y	Y	Y	Y	1	2	4	6
0.028	0.000	Y	Y	Y	Y	Y	1	4	8	9
0.112	0.022	Y	N	Y	Y	Y	0	0	1	3
0.100	0.023	Y	N	Y	Y	Y	0	0	1	3
0.085	0.048	N	N	N	Y	Y	0	2	4	6
0.082	0.025	N	N	Y	Y	Y	1	2	4	6
0.069	0.052	N	N	N	N	Y	2	2	5	7

**Table 8:** Set 3, trials 11–20 summary data (actual PR value).

Average Error	Avg Err	Lowest	Correct				False			
			At 0.01	At 0.025	At 0.05	At 0.1	At 0.01	At 0.025	At 0.05	At 0.10
0.056	0.002	Y	Y	Y	Y	Y	2	4	6	8
0.054	0.009	Y	Y	Y	Y	Y	0	2	7	8
0.058	0.062	N	N	N	N	Y	1	2	7	8
0.092	0.007	Y	Y	Y	Y	Y	0	2	4	6
0.068	0.104	N	N	N	N	N	1	3	7	8
0.054	0.027	N	N	N	Y	Y	0	3	7	8
0.054	0.012	N	N	Y	Y	Y	0	2	7	8
0.084	0.047	N	N	N	Y	Y	0	1	4	7
0.049	0.004	N	Y	Y	Y	Y	1	5	7	8
0.093	0.198	N	N	N	N	N	0	1	1	6

**Table 9:** Set 3, trials 11–20 summary data (all 0.5 PR values).

Average Error	Avg Err	Lowest	Correct				False			
			At 0.01	At 0.025	At 0.05	At 0.1	At 0.01	At 0.025	At 0.05	At 0.10
0.396	0.357	N	N	N	N	N	0	0	0	0
0.416	0.415	N	N	N	N	N	0	0	0	0
0.416	0.477	N	N	N	N	N	0	0	0	0
0.416	0.464	N	N	N	N	N	0	0	0	0
0.416	0.380	N	N	N	N	N	0	0	0	0
0.416	0.452	N	N	N	N	N	0	0	0	0
0.416	0.437	N	N	N	N	N	0	0	0	0
0.416	0.453	N	N	N	N	N	0	0	0	0
0.416	0.443	N	N	N	N	N	0	0	0	0
0.416	0.164	Y	N	N	N	N	0	0	0	0

**Table 10:** Set 3, trials 21–30 summary data (actual PR value).

Average Error	Avg Err	Lowest	Correct				False			
			At 0.01	At 0.025	At 0.05	At 0.1	At 0.01	At 0.025	At 0.05	At 0.10
0.132	0.007	Y	Y	Y	Y	Y	0	0	0	2
0.075	0.041	N	N	N	Y	Y	0	0	4	7
0.082	0.029	N	N	N	Y	Y	0	1	1	7
0.071	0.015	Y	N	Y	Y	Y	0	0	3	6
0.082	0.057	N	N	N	N	Y	0	0	3	6
0.111	0.022	Y	N	Y	Y	Y	0	0	1	3
0.097	0.094	N	N	N	N	Y	1	1	1	5
0.061	0.086	N	N	N	N	Y	1	2	4	7
0.054	0.006	N	Y	Y	Y	Y	2	5	5	7
0.111	0.023	Y	N	Y	Y	Y	0	0	0	2

**Table 11:** Set 3, trials 41–50 summary data (single 0.5 PR value).

Average Error	Avg Err	Lowest	Correct				False			
			At 0.01	At 0.025	At 0.05	At 0.1	At 0.01	At 0.025	At 0.05	At 0.10
0.058	0.005	Y	Y	Y	Y	Y	0	0	4	8
0.029	0.011	N	N	Y	Y	Y	3	4	7	9
0.135	0.096	N	N	N	N	Y	0	1	1	2
0.075	0.010	N	N	Y	Y	Y	1	1	3	7
0.030	0.000	Y	Y	Y	Y	Y	1	3	8	9
0.112	0.022	Y	N	Y	Y	Y	0	0	1	3
0.099	0.023	Y	N	Y	Y	Y	0	0	1	3
0.048	0.018	N	N	Y	Y	Y	1	4	5	8
0.027	0.019	N	N	Y	Y	Y	2	6	7	9
0.070	0.013	Y	N	Y	Y	Y	0	0	2	8

**Table 12:** Summary of standout data.

Model (Set)	1	2	3	3	3	3
Default Fact Value	000.600	000.600	000.500	000.500	000.600	000.550
PR Approach	Actual PR value	Actual PR value	Single 0.5 PR value	Actual PR value	Actual PR value	Single 0.5 PR value
Best Error Margin	0.01	0.01	0.025	0.01	0.025	0.025
Correct Patients	3	3	7	4	5	9
Avg Incorrect Patients	0.6	0.5	1.7	0.5	0.9	1.9
Correct-to-Incorrect Ratio	5	5	4.12	8	5.56	4.74

## Data Availability

No new data were collected during this study.

## Appendix A

This appendix provides an additional discussion of the technical implementation of the GDES system, building upon what is presented in Section 3.2. For the purposes of simplicity GUIDs will be written with the node name replacing the actual GUID value. Thus, the command:

SF:{24da3290-e934-4a9c-84e9-6a0d856e5073} = 000.393

would be presented as:

SF:{Heart Rate} = 000.393

Set fact commands are issued for each of the variables used in the given tests. Once the SF commands are issued, a training (TR) command is then issued. Training commands begin with a reference to a starting fact. As shown below, the blood pressure fact is used as the starting fact and the second GUID included in the TR command is that of the output fact. To train the network, this combination of starting fact and final fact is used:

SF:{Blood Pressure} = 000.459

SF:{Heart Rate} = 000.393

SF:{Respiratory Rate} = 000.222

SF:{End-tidal Carbon Dioxide} = 000.860

TR:{Blood Pressure} = 000.459>0001:0.05>{Output} = 000.500

Note that one of the SF commands is vestigial and not strictly necessary, as the initial fact included in a TR command is set by the TR command; however, all SF commands were issued for simplicity, as this approach allows the TR command issued to be changed without requiring changing the block of SF commands (and the issuance of an additional SF does not materially impact system operations). In the above example, the vestigial SF is for the blood pressure fact, which is set to the same value in lines 1 and 5 of the command text. The TR command sets the blood pressure fact to 000.459, even though it follows a SF command that has already set the blood pressure fact value to 000.459. While redundant, this is performed for simplicity and does not impact the network’s output.

In this example, 000.459 is the blood pressure (NBP) value from the data row being used for training. The value of 000.500, which is the midpoint of the valid output range, is used in all cases. This approach makes the trained patient the middle output value with other patients able to show a positive or negative deviation from this patient.

These commands, four SF commands followed by one TR command, are repeated for each row of data in the patient spreadsheets (60,000 records). Following these commands, a present (PR) command is issued. Two forms of this command were utilized. For some tests, a set value (e.g., 000.500) was used that is at or near the middle of the allowable fact value range:

PR:{Blood Pressure} = 000.500>>{Output}

For each set, in trials 1 through 20, the value of 000.500 was used. In trials 21 through 30 the value of 000.600 was used, trials 31 through 40 used 000.700, and trials 41 through 50 used 000.550.

A second approach utilized the blood pressure value from the last training row (so as to produce a natural output from the network). The form of this command was the same, except that the value assigned to blood pressure varied:

PR:{Blood Pressure} = 000.443>>{Output}

A third approach uses a set value, just like the first approach. The difference is that, just prior to the PR command being issued, all four facts are also set to that value:

SF:{Blood Pressure} = 000.500

SF:{Heart Rate} = 000.500

SF:{Respiratory Rate} = 000.500

SF:{End-tidal Carbon Dioxide} = 000.500

PR:{Blood Pressure} = 000.500>>{Output}

Finally, a fourth approach follows a similar pattern with one primary exception. The average value of each fact is calculated and used, instead of the set value in the SF command. The PR command again uses the value from blood pressure:

SF:{Blood Pressure} = 000.446

SF:{Heart Rate} = 000.399

SF:{Respiratory Rate} = 000.302

SF:{End-tidal Carbon Dioxide} = 000.812

PR:{Blood Pressure} = 000.446>>{Output}

The results from these four different approaches to generating a baseline value for a given patient are compared. The result of this PR command is what all other results, which are based on performing PRs using data from later in the patient data sets, were compared against. It is referred to as the initial training output value or the target value.

## Appendix B

This appendix presents data and limited analysis collected during the trails described in Section 4 of the paper.

### Appendix B.1

#### Appendix B.1.1. Trials 1–10

Table A1 presents data collected using a single 0.5 input to the PR command. Table A2 presents data collected using an actual value, from the dataset, as the PR command input. Table A3 presents data collected by supplying 0.5 values as all inputs before the PR command. Finally, Table A4 uses the average of all values for a field as the PR command input.

**Table A1:** Set 1, trials 1–10 data (single 0.5 PR value).

Actual	Average	Average Error	Max Error	Min Error
0.512	0.529	0.037	0.053	0.023
0.492	0.523	0.044	0.064	0.032
0.654	0.538	0.129	0.144	0.110
0.476	0.538	0.064	0.083	0.053
0.514	0.527	0.034	0.052	0.024
0.533	0.535	0.045	0.061	0.035
0.464	0.523	0.059	0.077	0.044
0.532	0.538	0.056	0.073	0.042
0.517	0.538	0.050	0.067	0.042
0.684	0.538	0.153	0.168	0.134

**Table A2:** Set 1, trials 1–10 data (actual PR value).

Actual	Average	Average Error	Max Error	Min Error
0.501	0.548	0.054	0.076	0.038
0.492	0.523	0.044	0.064	0.032
0.723	0.584	0.153	0.190	0.125
0.706	0.584	0.142	0.178	0.118
0.502	0.553	0.055	0.076	0.039
0.500	0.572	0.082	0.114	0.067
0.464	0.523	0.059	0.077	0.044
0.511	0.584	0.099	0.137	0.079
0.529	0.568	0.090	0.123	0.072
0.889	0.584	0.305	0.341	0.268

**Table A3:** Set 1, trials 1–10 data (all 0.5 PR values).

Actual	Average	Average Error	Max Error	Min Error
0.500	0.529	0.039	0.055	0.025
0.500	0.523	0.043	0.064	0.030
0.500	0.538	0.052	0.068	0.045
0.500	0.538	0.052	0.068	0.045
0.500	0.527	0.039	0.055	0.024
0.500	0.535	0.047	0.062	0.036
0.500	0.523	0.043	0.064	0.030
0.500	0.538	0.052	0.068	0.045
0.500	0.538	0.052	0.068	0.045
0.500	0.538	0.052	0.068	0.045

**Table A4:** Set 1, trials 1–10 data (average actual PR values).

Actual	Average	Average Error	Max Error	Min Error
0.498	0.529	0.040	0.056	0.027
0.492	0.523	0.044	0.064	0.032
0.708	0.538	0.172	0.187	0.153
0.701	0.538	0.167	0.182	0.148
0.494	0.527	0.041	0.057	0.027
0.502	0.535	0.046	0.061	0.036
0.464	0.523	0.059	0.077	0.044
0.526	0.538	0.053	0.071	0.041
0.546	0.538	0.061	0.079	0.045
0.815	0.538	0.277	0.292	0.259

Table A5, Table A6, Table A7 and Table A8 contain data similar to Table A1, Table A2, Table A3 and Table A4. However, rather than averaging values from each patient, these tables present data from only the target patient. This data facilitates the assessment of the system’s performance in the identification of the target patient. In these tables, row one corresponds to trial one and patient one, row two corresponds to trial two and patient two, and so forth.

**Table A5:** Set 1, trials 1–10 data (single 0.5 PR value).

Test Avg	Avg Err	Min	Max
0.500	0.012	0.007	0.020
0.527	0.035	0.027	0.064
0.717	0.064	0.060	0.067
0.486	0.010	0.004	0.026
0.482	0.033	0.011	0.049
0.536	0.004	0.011	0.019
0.474	0.010	0.007	0.019
0.508	0.024	0.015	0.041
0.515	0.002	0.004	0.011
0.584	0.100	0.056	0.126

**Table A6:** Set 1, trials 1–10 data (actual PR value).

Test Avg	Avg Err	Min	Max
0.493	0.007	0.002	0.016
0.527	0.035	0.027	0.064
0.792	0.069	0.059	0.080
0.716	0.010	0.004	0.026
0.487	0.015	0.016	0.051
0.524	0.024	0.004	0.044
0.474	0.010	0.007	0.019
0.485	0.026	0.002	0.049
0.516	0.013	0.009	0.024
0.709	0.180	0.130	0.302

**Table A7:** Set 1, trials 1–10 data (all 0.5 PR values).

Test Avg	Avg Err	Min	Max
0.500	0.000	0.005	0.007
0.527	0.027	0.019	0.056
0.717	0.217	0.213	0.221
0.486	0.014	0.002	0.028
0.482	0.018	0.003	0.034
0.536	0.036	0.022	0.052
0.474	0.026	0.017	0.043
0.508	0.008	0.010	0.017
0.515	0.015	0.005	0.021
0.584	0.084	0.058	0.128

**Table A8:** Set 1, trials 1–10 data (average actual PR values).

Test Avg	Avg Err	Min	Max
0.500	0.002	0.005	0.007
0.527	0.035	0.027	0.064
0.717	0.010	0.005	0.013
0.486	0.215	0.199	0.229
0.482	0.012	0.009	0.029
0.536	0.035	0.020	0.050
0.474	0.010	0.007	0.019
0.508	0.018	0.010	0.036
0.515	0.032	0.026	0.041
0.584	0.231	0.187	0.257

Table A9 presents data collected by dividing the values in Table A5 by the values in Table A1. Each row’s % err avg is calculated by the corresponding row of Table A5’s avg err divided by Table A1’s average error row. Likewise, % max avg and % min avg are the results of values from Table A5 being divided by corresponding values from Table A1. The bottom row contains the averages of each column. The lower the % err avg values, the closer the system output is to the target patient. A value of 100% would indicate that the target patient’s margin of error is equal to the average of all patients. A value greater than 100% indicates that the target patient’s margin of error is greater than the average of margin of error among all patients. Likewise, a value less than 100% indicates that the target patient had a lower margin of error than the average patient in that given trial. A low value indicates that the combination of the GDES network, default weight, and method for converting data to values 0 to 1 are effective at eliminating the incorrect patients from the pool of possible true identities.

**Table A9:** Set 1, trials 1–10 data (single 0.5 PR value).

% Err Avg	% Max Avg	% Min Avg
33.8%	37.0%	31.2%
78.1%	200.6%	41.2%
49.5%	60.8%	41.4%
15.5%	48.8%	4.8%
94.5%	93.6%	45.5%
8.2%	55.3%	17.8%
17.6%	43.6%	9.1%
43.0%	56.4%	35.5%
4.0%	16.5%	9.6%
65.2%	75.2%	41.4%
40.9%	68.78%	27.75%

**Table A10:** Set 1, trials 1–10 data (actual PR value).

% Err Avg	% Max Avg	% Min Avg
13.7%	21.4%	5.5%
78.1%	200.6%	41.2%
45.2%	63.7%	31.1%
7.1%	22.0%	2.3%
27.8%	67.1%	41.7%
29.8%	65.2%	3.7%
17.6%	43.6%	9.1%
26.6%	35.9%	3.2%
14.8%	19.1%	11.7%
59.1%	88.4%	48.6%
32.0%	62.7%	19.8%

**Table A11:** Set 1, trials 1–10 data (all 0.5 PR values).

% Err Avg	% Max Avg	% Min Avg
0.11%	20.70%	12.98%
61.26%	187.18%	28.79%
420.59%	495.51%	313.93%
27.12%	41.27%	4.49%
47.57%	62.57%	13.13%
77.64%	144.56%	35.23%
58.96%	66.93%	57.34%
14.67%	37.08%	14.00%
28.09%	46.07%	8.11%
162.56%	287.64%	84.75%
89.86%	138.95%	57.28%

**Table A12:** Set 1, trials 1–10 data (average actual PR values).

% Err Avg	% Max Avg	% Min Avg
5.63%	28.17%	8.89%
78.14%	200.63%	41.25%
5.61%	8.48%	2.94%
129.04%	134.32%	126.10%
30.33%	49.66%	33.94%
75.23%	140.07%	32.97%
17.65%	43.63%	9.12%
34.52%	50.35%	23.09%
51.31%	56.48%	51.59%
83.27%	88.03%	72.15%
51.07%	79.98%	40.20%

Table A13 presents results from further analyzing the data. The average error and avg err columns in this table are the same values as in Table A1 and Table A5. The lowest column contains a yes or no value. Yes indicates that the target patient had the lowest margin of error of all patients, while no indicates that this was not the case. Following this are four columns labeled correct at X.XX. These columns indicate whether the target patient’s value falls below the value listed for the column. Corresponding to these columns are the false at X.XX columns. These columns display the number of patients, other than the target patient, that have an average margin of error below the listed value.

An example of how to interpret the first row of Table A13 is as follows: the average error is 0.037, while the avg err is 0.012. This indicates that, in trial 1, patient one had a margin of error that is lower than the average of the patients. However, but because the lowest column indicates N, patient one does not have the lowest margin of error. The correct at X.XX columns in the first row read N, Y, Y, Y. This indicates that the target patient’s margin of error is above 0.01, but below 0.025. The False at X.XX builds on this by indicating that no patients had a margin of error below 0.01, but that five incorrect patients are below 0.025, and seven incorrect patients below 0.05. Interpreting all ten rows of the table will yield a clearer picture of how effectively the first GDES network works with a default weighting value of 0.5.

In this case, at an error margin of 0.01, there are two trials for which the target patient falls within the margin with an average of 0.8 incorrect patients also being included. This produces a correct-to-incorrect ratio of 2.5, the highest at any margin of error in Table A13. In Table A14, Table A15 and Table A16 the highest ratios and their error margins are 2.5 at 0.025, 1.4286 at 0.01, and 3.333 at 0.01, respectively. This indicates that in this batch of trials the fourth approach for running the PR command performs the best, in terms of this metric.

**Table A13:** Set 1, trials 1–10 summary data (single 0.5 PR value).

Average Error	Avg Err	Lowest	Correct				False			
			At 0.01	At 0.025	At 0.05	At 0.1	At 0.01	At 0.025	At 0.05	At 0.10
0.037	0.012	N	N	Y	Y	Y	0	5	7	8
0.044	0.035	N	N	N	Y	Y	1	5	7	8
0.129	0.064	N	N	N	N	Y	0	0	0	2
0.064	0.010	N	N	Y	Y	Y	1	2	6	6
0.034	0.033	N	N	N	Y	Y	3	5	7	8
0.045	0.004	Y	Y	Y	Y	Y	0	2	6	8
0.059	0.010	N	N	Y	Y	Y	1	3	4	8
0.056	0.024	N	N	Y	Y	Y	0	2	5	8
0.050	0.002	Y	Y	Y	Y	Y	2	3	5	8
0.153	0.100	N	N	N	N	Y	0	0	1	2

**Table A14:** Set 1, trials 1–10 summary data (actual PR value).

Average Error	Avg Err	Lowest	Correct				False			
			At 0.01	At 0.025	At 0.05	At 0.1	At 0.01	At 0.025	At 0.05	At 0.10
0.054	0.007	Y	Y	Y	Y	Y	1	3	5	7
0.044	0.035	N	N	N	Y	Y	1	5	7	8
0.153	0.069	N	N	N	N	Y	1	2	2	3
0.142	0.010	N	Y	Y	Y	Y	1	1	1	3
0.055	0.015	N	N	Y	Y	Y	1	3	5	6
0.082	0.024	N	N	Y	Y	Y	3	3	5	5
0.059	0.010	N	N	Y	Y	Y	1	3	4	8
0.099	0.026	N	N	N	Y	Y	1	3	4	5
0.090	0.013	Y	N	Y	Y	Y	0	1	5	6
0.305	0.180	N	N	N	N	N	0	0	0	1

**Table A15:** Set 1, trials 1–10 summary data (all 0.5 PR values).

Average Error	Avg Err	Lowest	Correct				False			
			At 0.01	At 0.025	At 0.05	At 0.1	At 0.01	At 0.025	At 0.05	At 0.10
0.039	0.000	Y	Y	Y	Y	Y	1	4	7	7
0.043	0.027	N	N	N	Y	Y	1	2	7	8
0.052	0.217	N	N	N	N	N	2	6	7	8
0.052	0.014	N	N	Y	Y	Y	2	5	6	7
0.039	0.018	N	N	Y	Y	Y	1	6	7	7
0.047	0.036	N	N	N	Y	Y	1	5	6	8
0.043	0.026	N	N	N	Y	Y	1	2	7	8
0.052	0.008	N	Y	Y	Y	Y	1	5	6	7
0.052	0.015	N	N	Y	Y	Y	2	5	6	7
0.052	0.084	N	N	N	N	Y	2	6	7	7

**Table A16:** Set 1, trials 1–10 summary data (average actual PR values).

Average Error	Avg Err	Lowest	Correct				False			
			At 0.01	At 0.025	At 0.05	At 0.1	At 0.01	At 0.025	At 0.05	At 0.10
0.040	0.002	Y	Y	Y	Y	Y	1	3	7	7
0.044	0.035	N	N	N	Y	Y	1	5	7	8
0.172	0.010	Y	Y	Y	Y	Y	0	0	0	0
0.167	0.215	N	N	N	N	N	0	1	1	2
0.041	0.012	N	N	Y	Y	Y	1	4	7	7
0.046	0.035	N	N	N	Y	Y	1	5	6	8
0.059	0.010	N	N	Y	Y	Y	1	3	4	8
0.053	0.018	N	N	Y	Y	Y	1	3	5	8
0.061	0.032	N	N	N	Y	Y	0	0	4	8
0.277	0.231	N	N	N	N	N	0	0	0	1

#### Appendix B.1.2. Trials 11–20

The tables in this and subsequent sections are structured in the same way as those previously described and can therefore be interpreted in the same fashion. Sets of trials that are notable are specifically discussed in this and subsequent sections.

As shown in Table A25, the % err avg average value is greater than 100%, indicating that the target patients, in general, have a greater margin of error than most other patients in the trials. Table A29 indicates that in only one trial was the target patient closest to the system output. It also indicates that, at an error margin of 0.05, only half of the trials contain the target patient, and in those cases, there are at least six (though usually seven or more) other patients that also fall within that margin. At an error margin of 0.025 the highest correct-to-incorrect patient ratio is attained as 1.22. In Table A30 and Table A31 the ratios from the other PR approaches are 1.4 at 0.05 and 1.36 at 0.05. Table 1 shows that, in that case, no patients correct or incorrect fell within a 0.1 margin of error. Thus, the PR approach that produces the highest ratio, in this case, is the second. It is not until the margin is increased to 0.1 that the target patient falls within the range for each trial, though in every trial there are at least eight other patients that fall within this range. Set 1, trials 11–20 are the least effective identification method tested. It uses the first GDES network, a default weight of 0.5, and the isolated data conversion method.

**Table A17:** Set 1, trials 11–20 data (single 0.5 PR value).

Actual	Average	Average Error	Max Error	Min Error
0.750	0.704	0.046	0.074	0.014
0.674	0.714	0.041	0.064	0.037
0.679	0.706	0.043	0.068	0.031
0.679	0.696	0.024	0.058	0.035
0.734	0.704	0.032	0.056	0.016
0.728	0.694	0.035	0.067	0.019
0.728	0.704	0.028	0.052	0.019
0.750	0.704	0.046	0.070	0.017
0.706	0.708	0.024	0.050	0.022
0.684	0.699	0.033	0.061	0.027

**Table A18:** Set 1, trials 11–20 data (actual PR value).

Actual	Average	Average Error	Max Error	Min Error
0.982	0.913	0.069	0.185	0.012
0.861	0.901	0.063	0.153	0.087
0.911	0.901	0.053	0.163	0.053
0.911	0.901	0.053	0.163	0.053
0.984	0.909	0.075	0.212	0.018
0.959	0.902	0.067	0.179	0.020
0.959	0.908	0.059	0.180	0.023
1.000	0.885	0.115	0.241	0.034
0.933	0.894	0.063	0.184	0.036
0.889	0.901	0.058	0.158	0.066

**Table A19:** Set 1, trials 11–20 data (all 0.5 PR values).

Actual	Average	Average Error	Max Error	Min Error
0.500	0.699	0.199	0.231	0.168
0.500	0.704	0.204	0.233	0.180
0.500	0.704	0.204	0.233	0.180
0.500	0.704	0.204	0.233	0.180
0.500	0.704	0.204	0.233	0.180
0.500	0.704	0.204	0.233	0.180
0.500	0.704	0.204	0.233	0.180
0.500	0.704	0.204	0.233	0.180
0.500	0.704	0.204	0.233	0.180
0.500	0.704	0.204	0.233	0.180

**Table A20:** Set 1, trials 11–20 data (average actual PR values).

Actual	Average	Average Error	Max Error	Min Error
0.423	0.699	0.276	0.309	0.245
0.509	0.704	0.195	0.224	0.171
0.708	0.704	0.027	0.051	0.023
0.679	0.704	0.041	0.065	0.030
0.454	0.704	0.250	0.279	0.226
0.449	0.704	0.255	0.284	0.232
0.648	0.704	0.063	0.090	0.045
0.526	0.538	0.053	0.071	0.041
0.546	0.704	0.158	0.187	0.134
0.815	0.704	0.111	0.135	0.082

**Table A21:** Set 1, trials 11–20 data (single 0.5 PR value).

Test Avg	Avg Err	Min	Max
0.665	0.085	0.017	0.130
0.693	0.019	0.034	0.064
0.743	0.064	0.060	0.067
0.693	0.014	0.039	0.059
0.668	0.066	0.010	0.104
0.668	0.059	0.016	0.098
0.716	0.012	0.018	0.037
0.726	0.024	0.007	0.055
0.708	0.002	0.013	0.013
0.611	0.073	0.026	0.102

**Table A22:** Set 1, trials 11–20 data (actual PR value).

Test Avg	Avg Err	Min	Max
0.976	0.006	0.001	0.016
0.818	0.042	0.115	0.190
0.976	0.066	0.051	0.082
0.968	0.058	0.031	0.090
0.857	0.127	0.003	0.273
0.927	0.032	0.026	0.243
0.927	0.032	0.026	0.243
0.916	0.084	0.028	0.127
0.918	0.015	0.029	0.228
0.802	0.087	0.022	0.275

**Table A23:** Set 1, trials 11–20 data (all 0.5 PR values).

Test Avg	Avg Err	Min	Max
0.665	0.165	0.121	0.233
0.693	0.193	0.140	0.238
0.743	0.243	0.239	0.246
0.718	0.218	0.191	0.250
0.668	0.168	0.130	0.244
0.722	0.222	0.216	0.243
0.716	0.216	0.191	0.245
0.726	0.226	0.195	0.243
0.708	0.208	0.193	0.218
0.611	0.111	0.082	0.158

**Table A24:** Set 1, trials 11–20 data (average actual PR values).

Test Avg	Avg Err	Min	Max
0.665	0.243	0.198	0.311
0.693	0.184	0.131	0.229
0.743	0.035	0.031	0.039
0.718	0.039	0.012	0.071
0.668	0.214	0.176	0.290
0.722	0.274	0.267	0.295
0.716	0.068	0.043	0.097
0.508	0.018	0.010	0.036
0.708	0.162	0.147	0.172
0.611	0.204	0.157	0.233

**Table A25:** Set 1, trials 11–20 data (single 0.5 PR value).

% Err Avg	% MaxAX Avg	% Min Avg
186.7%	174.8%	125.5%
46.9%	172.3%	52.8%
149.1%	218.6%	87.6%
60.5%	167.4%	67.8%
207.1%	186.9%	58.5%
168.6%	145.5%	84.0%
42.2%	93.6%	70.3%
51.2%	78.6%	40.2%
9.8%	57.6%	25.9%
219.8%	166.4%	92.9%
114.2%	146.17%	70.55%

**Table A26:** Set 1, trials 11–20 data (actual PR value).

% Err Avg	% Max Avg	% Min Avg
9.0%	8.7%	8.4%
67.1%	132.5%	124.4%
123.5%	154.1%	31.3%
108.9%	169.2%	18.7%
170.3%	128.5%	14.1%
47.2%	136.1%	133.0%
53.9%	134.7%	111.3%
72.9%	81.4%	52.5%
24.6%	123.9%	79.7%
150.8%	173.4%	32.5%
82.82%	124.25%	60.59%

**Table A27:** Set 1, trials 11–20 data (all 0.5 PR values).

% Err Avg	% Max Avg	% Min Avg
83.07%	139.02%	52.06%
94.77%	132.22%	60.19%
118.98%	136.67%	102.54%
107.07%	138.89%	82.12%
82.41%	135.28%	55.89%
108.99%	135.00%	92.65%
105.77%	136.11%	82.12%
111.03%	135.00%	83.83%
101.92%	121.11%	82.76%
54.22%	87.78%	35.25%
96.82%	129.71%	72.94%

**Table A28:** Set 1, trials 11–20 data (average actual PR values).

% Err Avg	% Max Avg	% Min Avg
87.82%	126.68%	64.09%
94.53%	133.92%	58.59%
132.33%	168.86%	60.90%
96.43%	233.55%	18.35%
85.65%	128.10%	63.17%
107.18%	127.21%	93.98%
106.76%	216.52%	47.67%
34.52%	50.35%	23.09%
102.48%	128.36%	78.51%
184.46%	191.09%	172.86%
103.22%	150.46%	68.12%

**Table A29:** Set 1, trials 11–20 summary data (single 0.5 PR value).

Average Error	Avg Err	Lowest	Correct				False			
			At 0.01	At 0.025	At 0.05	At 0.1	At 0.01	At 0.025	At 0.05	At 0.10
0.046	0.085	N	N	N	N	Y	0	4	7	8
0.041	0.019	N	N	Y	Y	Y	1	1	7	9
0.043	0.064	N	N	N	N	Y	0	2	7	9
0.024	0.014	N	N	Y	Y	Y	0	5	8	9
0.032	0.066	N	N	N	N	Y	3	6	8	8
0.035	0.059	N	N	N	N	Y	3	5	7	8
0.028	0.012	N	N	Y	Y	Y	4	6	7	8
0.046	0.024	N	N	Y	Y	Y	1	2	6	8
0.024	0.002	Y	Y	Y	Y	Y	0	6	8	9
0.033	0.073	N	N	N	N	Y	2	4	8	9

**Table A30:** Set 1, trials 11–20 summary data (actual PR value).

Average Error	Avg Err	Lowest	Correct				False			
			At 0.01	At 0.025	At 0.05	At 0.1	At 0.01	At 0.025	At 0.05	At 0.10
0.069	0.006	N	Y	Y	Y	Y	1	2	2	6
0.063	0.042	N	N	N	Y	Y	2	2	2	6
0.053	0.066	N	N	N	N	Y	2	3	3	8
0.053	0.058	N	N	N	N	Y	2	3	3	8
0.075	0.127	N	N	N	N	N	2	3	3	7
0.067	0.032	N	N	N	Y	Y	0	3	5	5
0.059	0.032	N	N	N	Y	Y	1	3	6	6
0.115	0.084	N	N	N	N	Y	0	1	2	4
0.063	0.015	N	N	Y	Y	Y	1	1	4	7
0.058	0.087	N	N	N	N	Y	0	0	5	9

**Table A31:** Set 1, trials 11–20 summary data (average actual PR values).

Average Error	Avg Err	Lowest	Correct				False			
			At 0.01	At 0.025	At 0.05	At 0.1	At 0.01	At 0.025	At 0.05	At 0.10
0.276	0.243	N	N	N	N	N	0	0	0	0
0.195	0.184	N	N	N	N	N	0	0	0	0
0.027	0.035	N	N	N	Y	Y	2	6	8	9
0.041	0.039	N	N	N	Y	Y	0	2	6	9
0.250	0.214	N	N	N	N	N	0	0	0	0
0.255	0.274	N	N	N	N	N	0	0	0	0
0.063	0.068	N	N	N	N	Y	0	1	3	9
0.053	0.018	N	N	Y	Y	Y	1	3	5	8
0.158	0.162	N	N	N	N	N	0	0	0	1
0.111	0.204	N	N	N	N	N	0	0	0	6

#### Appendix B.1.3. Trials 21–30

Data from trials 21 to 30 are presented in Table A32, Table A33, Table A34, Table A35, Table A36, Table A37, Table A38, Table A39, Table A40, Table A41, Table A42, Table A43, Table A44, Table A45 and Table A46. As seen in Table A40, the % err avg for Set 1, trials 21 through 30 is 41.6, indicating that the target patient had a lower margin of error than most other patients in those trials. Table A44 indicates that the target patient had the lowest margin of error in two trials. However, at a 0.05 margin, there were only two trials that did not include the target patient and an average of 3.6 incorrectly identified patients. This yields a correct-to-incorrect ratio of 2.22. At an error margin of 0.025, six correct patients fall within the margin with an average of 2.1 incorrect patients per trial. This is a higher ratio of 2.86, the highest of any error margin in these ten trials using the first PR approach. The highest ratios found in Table 2, Table A45 and Table A46 are 5 at 0.01, 1.36 at 0.05, and 3.33 at 0.01, respectively. Thus, the second PR approach performs best in this case.

**Table A32:** Set 1, trials 21–30 data (single 0.5 PR value).

Actual	Average	Average Error	Max Error	Min Error
0.552	0.532	0.053	0.074	0.044
0.492	0.532	0.053	0.073	0.033
0.557	0.532	0.055	0.078	0.045
0.456	0.532	0.076	0.095	0.050
0.512	0.532	0.049	0.069	0.032
0.640	0.557	0.085	0.105	0.073
0.464	0.532	0.068	0.087	0.044
0.633	0.560	0.079	0.098	0.067
0.467	0.532	0.065	0.084	0.042
0.734	0.588	0.153	0.168	0.134

**Table A33:** Set 1, trials 21–30 data (actual PR value).

Actual	Average	Average Error	Max Error	Min Error
0.552	0.532	0.053	0.074	0.044
0.492	0.532	0.053	0.073	0.033
0.557	0.532	0.055	0.078	0.045
0.456	0.532	0.076	0.095	0.050
0.512	0.532	0.049	0.069	0.032
0.599	0.559	0.061	0.085	0.047
0.464	0.532	0.068	0.087	0.044
0.598	0.561	0.062	0.085	0.045
0.467	0.532	0.065	0.084	0.042
0.889	0.591	0.297	0.335	0.258

**Table A34:** Set 1, trials 21–30 data (all 0.5 PR values).

Actual	Average	Average Error	Max Error	Min Error
0.600	0.532	0.085	0.112	0.068
0.600	0.532	0.085	0.112	0.068
0.600	0.532	0.085	0.112	0.068
0.600	0.532	0.085	0.112	0.068
0.600	0.532	0.085	0.112	0.068
0.600	0.557	0.059	0.078	0.050
0.600	0.532	0.085	0.112	0.068
0.600	0.560	0.058	0.076	0.050
0.600	0.532	0.085	0.112	0.068
0.889	0.591	0.297	0.335	0.258

**Table A35:** Set 1, trials 21–30 data (average actual PR values).

Actual	Average	Average Error	Max Error	Min Error
0.552	0.532	0.053	0.074	0.044
0.492	0.532	0.053	0.073	0.033
0.557	0.532	0.055	0.078	0.045
0.456	0.532	0.076	0.095	0.050
0.512	0.532	0.049	0.069	0.032
0.600	0.557	0.059	0.078	0.050
0.464	0.532	0.068	0.087	0.044
0.605	0.560	0.060	0.079	0.053
0.467	0.532	0.065	0.084	0.042
0.815	0.588	0.227	0.242	0.209

**Table A36:** Set 1, trials 21–30 data (single 0.5 PR value).

Test Avg	Avg Err	Min	Max
0.527	0.024	0.018	0.036
0.527	0.035	0.027	0.064
0.555	0.002	0.017	0.021
0.472	0.016	0.010	0.019
0.481	0.030	0.018	0.044
0.623	0.017	0.027	0.051
0.474	0.010	0.007	0.019
0.549	0.083	0.071	0.098
0.475	0.008	0.008	0.008
0.634	0.100	0.056	0.126

**Table A37:** Set 1, trials 21–30 data (actual PR value).

Test Avg	Avg Err	Min	Max
0.527	0.024	0.018	0.036
0.527	0.035	0.027	0.064
0.555	0.002	0.017	0.021
0.472	0.016	0.010	0.019
0.481	0.030	0.018	0.044
0.597	0.002	0.040	0.051
0.474	0.010	0.007	0.019
0.513	0.085	0.069	0.102
0.475	0.008	0.008	0.008
0.716	0.173	0.130	0.252

**Table A38:** Set 1, trials 21–30 data (all 0.5 PR values).

Test Avg	Avg Err	Min	Max
0.527	0.073	0.066	0.085
0.527	0.073	0.045	0.082
0.555	0.045	0.027	0.064
0.472	0.128	0.126	0.135
0.481	0.119	0.106	0.133
0.623	0.023	0.011	0.067
0.474	0.126	0.117	0.143
0.549	0.051	0.039	0.065
0.475	0.126	0.126	0.126
0.634	0.034	0.008	0.078

**Table A39:** Set 1, trials 21–30 data (average actual PR values).

Test Avg	Avg Err	Min	Max
0.527	0.024	0.018	0.036
0.527	0.035	0.027	0.064
0.555	0.002	0.017	0.021
0.472	0.016	0.010	0.019
0.481	0.030	0.018	0.044
0.623	0.023	0.011	0.067
0.474	0.010	0.007	0.019
0.549	0.056	0.044	0.070
0.475	0.008	0.008	0.008
0.634	0.181	0.137	0.207

**Table A40:** Set 1, trials 21–30 data (single 0.5 PR value).

% Err Avg	% Max Avg	% Min Avg
45.6%	48.6%	39.6%
65.5%	194.8%	36.2%
3.4%	36.7%	26.4%
21.1%	36.9%	10.0%
61.6%	63.4%	54.3%
19.8%	48.9%	37.2%
15.4%	43.6%	8.1%
105.8%	106.3%	99.6%
12.3%	19.2%	9.5%
65.2%	75.2%	41.4%
41.6%	67.36%	36.23%

**Table A41:** Set 1, trials 21–30 data (actual PR value).

% Err Avg	% Max Avg	% Min Avg
45.6%	48.6%	39.6%
65.5%	194.8%	36.2%
3.4%	36.7%	26.4%
21.1%	36.9%	10.0%
61.6%	63.4%	54.3%
3.0%	107.8%	47.2%
15.4%	43.6%	8.1%
137.1%	152.8%	120.1%
12.3%	19.2%	9.5%
58.1%	75.1%	50.5%
42.31%	77.89%	40.19%

**Table A42:** Set 1, trials 21–30 data (all 0.5 PR values).

% Err Avg	% Max Avg	% Min Avg
85.57%	97.42%	75.21%
86.58%	72.54%	65.68%
53.48%	56.96%	39.85%
151.00%	185.24%	119.72%
139.89%	156.46%	117.94%
39.02%	132.99%	14.52%
148.05%	172.69%	127.28%
87.84%	85.21%	77.43%
147.97%	185.24%	111.70%
53.87%	167.20%	9.36%
99.33%	131.20%	75.87%

**Table A43:** Set 1, trials 21–30 data (average actual PR values).

% Err Avg	% Max Avg	% Min Avg
45.63%	48.55%	39.64%
65.46%	194.79%	36.15%
3.37%	36.71%	26.43%
21.05%	36.89%	10.03%
61.62%	63.40%	54.35%
39.97%	134.72%	13.96%
15.41%	43.63%	8.07%
93.53%	88.44%	82.73%
12.28%	19.16%	9.50%
79.59%	85.55%	65.47%
43.79%	75.18%	34.63%

**Table A44:** Set 1, trials 21–30 summary data (single 0.5 PR value).

Average Error	Avg Err	Lowest	Correct				False			
			At 0.01	At 0.025	At 0.05	At 0.1	At 0.01	At 0.025	At 0.05	At 0.10
0.053	0.024	N	N	Y	Y	Y	1	3	4	8
0.053	0.035	N	N	N	Y	Y	0	4	6	8
0.055	0.002	Y	Y	Y	Y	Y	0	1	4	8
0.076	0.016	Y	N	Y	Y	Y	0	2	3	7
0.049	0.030	N	N	N	Y	Y	0	2	7	8
0.085	0.017	N	N	Y	Y	Y	0	1	2	5
0.068	0.010	N	N	Y	Y	Y	1	3	3	7
0.079	0.083	N	N	N	N	Y	0	2	3	5
0.065	0.008	N	Y	Y	Y	Y	2	3	3	7
0.153	0.100	N	N	N	N	Y	0	0	1	2

**Table A45:** Set 1, trials 21–30 summary data (all 0.5 PR values).

Average Error	Avg Err	Lowest	Correct				False			
			At 0.01	At 0.025	At 0.05	At 0.1	At 0.01	At 0.025	At 0.05	At 0.10
0.085	0.073	N	N	N	N	Y	0	1	2	5
0.085	0.073	N	N	N	N	Y	0	1	2	5
0.085	0.045	N	N	N	Y	Y	0	1	1	5
0.085	0.128	N	N	N	N	N	0	1	2	6
0.085	0.119	N	N	N	N	N	0	1	2	6
0.059	0.023	N	N	Y	Y	Y	1	1	2	9
0.085	0.126	N	N	N	N	N	0	1	2	6
0.058	0.051	N	N	N	N	Y	1	2	3	9
0.085	0.126	N	N	N	N	N	0	1	2	6
0.063	0.034	N	N	N	Y	Y	0	0	4	8

**Table A46:** Set 1, trials 21–30 summary data (average actual PR values).

Average Error	Avg Err	Lowest	Correct				False			
			At 0.01	At 0.025	At 0.05	At 0.1	At 0.01	At 0.025	At 0.05	At 0.10
0.053	0.024	N	N	Y	Y	Y	1	3	4	8
0.053	0.035	N	N	N	Y	Y	0	4	6	8
0.055	0.002	Y	Y	Y	Y	Y	0	1	4	8
0.076	0.016	Y	N	Y	Y	Y	0	2	3	7
0.049	0.030	N	N	N	Y	Y	0	2	7	8
0.059	0.023	N	N	Y	Y	Y	1	1	2	9
0.068	0.010	N	N	Y	Y	Y	1	3	3	7
0.060	0.056	N	N	N	N	Y	1	2	3	8
0.065	0.008	N	Y	Y	Y	Y	2	3	3	7
0.227	0.181	N	N	N	N	N	0	0	1	1

#### Appendix B.1.4. Trials 31–40

Data from trials 31 to 40, for the first network, are presented in Table A47, Table A48, Table A49, Table A50, Table A51, Table A52, Table A53, Table A54, Table A55, Table A56, Table A57, Table A58, Table A59, Table A60, Table A61 and Table A62. In this data, the system was tested with the first GDES network, a default weight of 0.7, and the comprehensive data conversion method. Table A55 indicates an average % err avg value of 40.4%, indicating that in most trials the target patient had a lower margin of error than the average. Table A59 shows that in three of the trials the target patient had the lowest margin of error. At an error margin of 0.05, seven of the target patients fall within that range and, in all but two of the trials, there are three or fewer incorrect patients that are also within that range. Upon expanding the margin to 0.1 there is only one additional trial that includes the target patient while the number of incorrect patients also falling within the margin increases to seven in most of the trials. The margin of error with the highest correct-to-incorrect ratio is 0.01 with a ratio of 4. The highest ratios in Table A60, Table A61 and Table A62 were 4 at an error margin of 0.01, 1.11 at 0.025 and at 0.05, and 3.33 at 0.01. Thus, in this case, the first and second PR approach produce an equally strong ratio.

**Table A47:** Set 1, trials 31–40 data (single 0.5 PR value).

Actual	Average	Average Error	Max Error	Min Error
0.552	0.540	0.061	0.081	0.047
0.492	0.540	0.061	0.083	0.033
0.557	0.540	0.064	0.084	0.049
0.456	0.540	0.084	0.105	0.050
0.512	0.540	0.057	0.079	0.032
0.712	0.550	0.227	0.193	0.148
0.464	0.540	0.076	0.097	0.044
0.684	0.540	0.144	0.180	0.140
0.467	0.540	0.074	0.094	0.042
0.784	0.638	0.153	0.168	0.134

**Table A48:** Set 1, trials 31–40 data (actual PR value).

Actual	Average	Average Error	Max Error	Min Error
0.552	0.540	0.061	0.081	0.047
0.492	0.540	0.061	0.083	0.033
0.557	0.540	0.064	0.084	0.049
0.456	0.540	0.084	0.105	0.050
0.512	0.540	0.057	0.079	0.032
0.697	0.546	0.151	0.184	0.141
0.464	0.540	0.076	0.097	0.044
0.684	0.540	0.144	0.180	0.140
0.467	0.540	0.074	0.094	0.042
0.889	0.598	0.290	0.331	0.248

**Table A49:** Set 1, trials 31–40 data (all 0.5 PR values).

Actual	Average	Average Error	Max Error	Min Error
0.700	0.540	0.160	0.194	0.152
0.700	0.540	0.160	0.194	0.152
0.700	0.540	0.160	0.194	0.152
0.700	0.540	0.160	0.194	0.152
0.700	0.540	0.160	0.194	0.152
0.700	0.550	0.150	0.182	0.140
0.700	0.540	0.160	0.194	0.152
0.700	0.540	0.160	0.194	0.152
0.700	0.540	0.160	0.194	0.152
0.700	0.638	0.086	0.102	0.077

**Table A50:** Set 1, trials 31–40 data (average actual PR values).

Actual	Average	Average Error	Max Error	Min Error
0.552	0.540	0.061	0.081	0.047
0.492	0.540	0.061	0.083	0.033
0.557	0.540	0.064	0.084	0.049
0.456	0.540	0.084	0.105	0.050
0.512	0.540	0.057	0.079	0.032
0.698	0.550	0.147	0.179	0.138
0.464	0.540	0.076	0.097	0.044
0.684	0.540	0.144	0.180	0.140
0.467	0.540	0.074	0.094	0.042
0.815	0.638	0.177	0.192	0.159

**Table A51:** Set 1, trials 31–40 data (0.5 PR value).

Test Avg	Avg Err	Min	Max
0.527	0.024	0.018	0.036
0.527	0.035	0.027	0.064
0.555	0.002	0.017	0.021
0.472	0.016	0.010	0.019
0.481	0.030	0.018	0.044
0.674	0.103	0.035	0.091
0.474	0.010	0.007	0.019
0.541	0.143	0.128	0.154
0.475	0.008	0.008	0.008
0.684	0.100	0.056	0.126

**Table A52:** Set 1, trials 31–40 data (actual PR value).

Test Avg	Avg Err	Min	Max
0.527	0.024	0.018	0.036
0.527	0.035	0.027	0.064
0.555	0.002	0.017	0.021
0.472	0.016	0.010	0.019
0.481	0.030	0.018	0.044
0.664	0.033	0.038	0.089
0.474	0.010	0.007	0.019
0.541	0.143	0.128	0.154
0.475	0.008	0.008	0.008
0.723	0.166	0.130	0.212

**Table A53:** Set 1, trials 31–40 data (all 0.5 PR values).

Test Avg	Avg Err	Min	Max
0.527	0.173	0.166	0.185
0.527	0.173	0.145	0.182
0.555	0.145	0.127	0.164
0.472	0.228	0.226	0.235
0.481	0.219	0.206	0.233
0.674	0.026	0.046	0.080
0.474	0.226	0.217	0.243
0.541	0.159	0.144	0.170
0.475	0.226	0.226	0.226
0.684	0.016	0.028	0.043

**Table A54:** Set 1, trials 31–40 data (average actual PR values).

Test Avg	Avg Err	Min	Max
0.527	0.024	0.018	0.036
0.527	0.035	0.027	0.064
0.555	0.002	0.017	0.021
0.472	0.016	0.010	0.019
0.481	0.030	0.018	0.044
0.674	0.023	0.049	0.077
0.474	0.010	0.007	0.019
0.541	0.143	0.128	0.154
0.475	0.008	0.008	0.008
0.684	0.131	0.087	0.157

**Table A55:** Set 1, trials 31–40 data (single 0.5 PR value).

% Err Avg	% Max Avg	% Min Avg
39.2%	44.3%	37.2%
56.3%	194.8%	31.8%
2.9%	33.8%	24.5%
18.9%	36.9%	9.1%
52.4%	55.4%	54.3%
45.4%	47.3%	23.4%
13.7%	43.6%	7.2%
99.3%	90.8%	85.8%
10.9%	19.2%	8.5%
65.2%	75.2%	41.4%
40.4%	64.13%	32.32%

**Table A56:** Set 1, trials 31–40 data (actual PR value).

% Err Avg	% Max Avg	% Min Avg
39.2%	44.3%	37.2%
56.3%	194.8%	31.8%
2.9%	33.8%	24.5%
18.9%	36.9%	9.1%
52.4%	55.4%	54.3%
22.0%	48.3%	26.8%
13.7%	43.6%	7.2%
99.3%	90.8%	85.8%
10.9%	19.2%	8.5%
57.0%	63.9%	52.5%
37.26%	63.10%	33.77%

**Table A57:** Set 1, trials 31–40 data (all 0.5 PR values).

% Err Avg	% Max Avg	% Min Avg
108.01%	109.39%	95.18%
108.55%	95.22%	93.63%
90.98%	84.60%	83.69%
142.75%	148.60%	120.97%
136.85%	135.75%	119.94%
17.25%	44.01%	33.02%
141.18%	143.00%	125.35%
99.34%	94.56%	87.70%
141.14%	148.60%	116.33%
18.64%	41.85%	36.36%
100.47%	104.56%	91.22%

**Table A58:** Set 1, trials 31–40 data (average actual PR values).

% Err Avg	% Max Avg	% Min Avg
39.25%	44.28%	37.23%
56.32%	194.79%	31.81%
2.92%	33.81%	24.49%
18.91%	36.89%	9.07%
52.44%	55.42%	54.35%
15.73%	43.16%	35.37%
13.68%	43.63%	7.24%
99.26%	90.78%	85.77%
10.85%	19.16%	8.49%
73.60%	81.60%	54.32%
38.30%	64.35%	34.81%

**Table A59:** Set 1, trials 31–40 summary data (single 0.5 PR value).

Average Error	Avg Err	Lowest	Correct				False			
			At 0.01	At 0.025	At 0.05	At 0.1	At 0.01	At 0.025	At 0.05	At 0.10
0.061	0.024	N	N	Y	Y	Y	1	3	3	7
0.061	0.035	N	N	N	Y	Y	0	4	6	7
0.064	0.002	Y	Y	Y	Y	Y	0	1	3	7
0.084	0.016	Y	N	Y	Y	Y	0	2	3	7
0.057	0.030	N	N	N	Y	Y	0	2	7	7
0.227	0.103	Y	N	N	N	N	0	0	0	0
0.076	0.010	N	N	Y	Y	Y	1	3	3	7
0.144	0.143	N	N	N	N	N	1	2	2	2
0.074	0.008	N	Y	Y	Y	Y	2	3	3	7
0.153	0.100	N	N	N	N	Y	0	0	1	2

**Table A60:** Set 1, trials 31–40 summary data (actual PR value).

Average Error	Avg Err	Lowest	Correct				False			
			At 0.01	At 0.025	At 0.05	At 0.1	At 0.01	At 0.025	At 0.05	At 0.10
0.061	0.024	N	N	Y	Y	Y	1	3	3	7
0.061	0.035	N	N	N	Y	Y	0	4	6	7
0.064	0.002	Y	Y	Y	Y	Y	0	1	3	7
0.084	0.016	Y	N	Y	Y	Y	0	2	3	7
0.057	0.030	N	N	N	Y	Y	0	2	7	7
0.151	0.033	N	N	N	Y	Y	0	1	1	1
0.076	0.010	N	N	Y	Y	Y	1	3	3	7
0.144	0.143	N	N	N	N	N	1	2	2	2
0.074	0.008	N	Y	Y	Y	Y	2	3	3	7
0.290	0.166	N	N	N	N	N	0	0	0	1

**Table A61:** Set 1, trials 31–40 summary data (all 0.5 PR values).

Average Error	Avg Err	Lowest	Correct				False			
			At 0.01	At 0.025	At 0.05	At 0.1	At 0.01	At 0.025	At 0.05	At 0.10
0.160	0.173	N	N	N	N	N	0	1	2	2
0.160	0.173	N	N	N	N	N	0	1	2	2
0.160	0.145	N	N	N	N	N	0	1	2	2
0.160	0.228	N	N	N	N	N	0	1	2	2
0.160	0.219	N	N	N	N	N	0	1	2	2
0.150	0.026	Y	N	N	Y	Y	0	0	1	1
0.160	0.226	N	N	N	N	N	0	1	2	2
0.160	0.159	N	N	N	N	N	0	1	2	2
0.160	0.226	N	N	N	N	N	0	1	2	2
0.086	0.016	N	N	Y	Y	Y	1	1	1	5

**Table A62:** Set 1, trials 31–40 summary data (average actual PR values).

Average Error	Avg Err	Lowest	Correct				False			
			At 0.01	At 0.025	At 0.05	At 0.1	At 0.01	At 0.025	At 0.05	At 0.10
0.061	0.024	N	N	Y	Y	Y	1	3	3	7
0.061	0.035	N	N	N	Y	Y	0	4	6	7
0.064	0.002	Y	Y	Y	Y	Y	0	1	3	7
0.084	0.016	Y	N	Y	Y	Y	0	2	3	7
0.057	0.030	N	N	N	Y	Y	0	2	7	7
0.147	0.023	Y	N	Y	Y	Y	0	0	1	1
0.076	0.010	N	N	Y	Y	Y	1	3	3	7
0.144	0.143	N	N	N	N	N	1	2	2	2
0.074	0.008	N	Y	Y	Y	Y	2	3	3	7
0.177	0.131	N	N	N	N	N	1	1	1	1

#### Appendix B.1.5. Trials 41–50

Table A63, Table A64, Table A65, Table A66, Table A67, Table A68, Table A69, Table A70, Table A71, Table A72, Table A73, Table A74, Table A75, Table A76, Table A77 and Table A78 present data for set 1, trials 41 through 50. These trials used the first GDES network and a default weight of 0.7. The average % ERR AVG across the trials was 45.1%. At a margin of error of 0.05, seven target patients were within the correct range, with an average of 3.8 incorrect patients also within the margin. At 0.1 all ten target patients fall within the margin and an average of 6.7 incorrect patients are also within the margin. In Table A75, Table A76, Table A77 and Table A78, the highest correct-to-incorrect ratios are 2.22 at an error margin of 0.01, 2.27 at 0.025, 1.11 at 0.1, and 2.16 at 0.05, respectively. This indicates that, in this case, the highest performing PR approach is the second. At an error margin of 0.025 there were five target patients that fell within the margin with an average of 2.2 incorrectly identified patients per trial, as shown in Table A76.

**Table A63:** Set 1, trials 41–50 data (single 0.5 PR value).

Actual	Average	Average Error	Max Error	Min Error
0.551	0.528	0.050	0.073	0.038
0.492	0.521	0.045	0.065	0.030
0.679	0.563	0.129	0.144	0.110
0.501	0.563	0.064	0.083	0.053
0.512	0.527	0.045	0.064	0.032
0.591	0.550	0.058	0.074	0.050
0.464	0.527	0.063	0.082	0.044
0.585	0.555	0.057	0.073	0.046
0.467	0.527	0.061	0.079	0.042
0.709	0.563	0.153	0.168	0.134

**Table A64:** Set 1, trials 41–50 data (actual PR value).

Actual	Average	Average Error	Max Error	Min Error
0.550	0.525	0.051	0.073	0.037
0.492	0.527	0.049	0.068	0.033
0.723	0.587	0.149	0.186	0.120
0.706	0.587	0.139	0.174	0.113
0.512	0.527	0.045	0.064	0.032
0.549	0.565	0.060	0.086	0.046
0.464	0.527	0.063	0.082	0.044
0.549	0.574	0.073	0.104	0.057
0.467	0.527	0.061	0.079	0.042
0.889	0.587	0.301	0.337	0.263

**Table A65:** Set 1, trials 41–50 data (all 0.5 PR values).

Actual	Average	Average Error	Max Error	Min Error
0.550	0.528	0.050	0.073	0.038
0.550	0.527	0.050	0.073	0.039
0.550	0.563	0.053	0.070	0.041
0.550	0.563	0.053	0.070	0.041
0.550	0.527	0.050	0.073	0.039
0.550	0.550	0.043	0.059	0.030
0.550	0.527	0.050	0.073	0.039
0.550	0.555	0.045	0.061	0.033
0.550	0.527	0.050	0.073	0.039
0.550	0.563	0.053	0.070	0.041

**Table A66:** Set 1, trials 41–50 data (average actual PR values).

Actual	Average	Average Error	Max Error	Min Error
0.550	0.528	0.050	0.073	0.038
0.492	0.527	0.049	0.068	0.033
0.708	0.563	0.152	0.167	0.133
0.701	0.563	0.147	0.162	0.128
0.512	0.527	0.045	0.064	0.032
0.551	0.550	0.043	0.059	0.030
0.464	0.527	0.063	0.082	0.044
0.561	0.555	0.047	0.063	0.036
0.467	0.527	0.061	0.079	0.042
0.815	0.563	0.252	0.267	0.234

**Table A67:** Set 1, trials 41–50 data (single 0.5 PR value).

Test Avg	Avg Err	Min	Max
0.527	0.024	0.017	0.036
0.527	0.035	0.027	0.064
0.742	0.064	0.060	0.067
0.511	0.010	0.004	0.026
0.481	0.030	0.018	0.044
0.584	0.007	0.023	0.031
0.474	0.010	0.007	0.019
0.534	0.050	0.040	0.066
0.475	0.008	0.008	0.008
0.609	0.100	0.056	0.126

**Table A68:** Set 1, trials 41–50 data (actual PR value).

Test Avg	Avg Err	Min	Max
0.526	0.024	0.017	0.035
0.527	0.035	0.027	0.064
0.792	0.069	0.059	0.080
0.716	0.010	0.004	0.026
0.481	0.030	0.018	0.044
0.561	0.012	0.017	0.052
0.474	0.010	0.007	0.019
0.497	0.052	0.032	0.072
0.475	0.008	0.008	0.008
0.712	0.176	0.130	0.277

**Table A69:** Set 1, trials 41–50 data (all 0.5 PR values).

Test Avg	Avg Err	Min	Max
0.527	0.023	0.017	0.035
0.527	0.023	0.005	0.032
0.742	0.192	0.188	0.196
0.511	0.039	0.023	0.053
0.481	0.069	0.056	0.083
0.584	0.034	0.010	0.064
0.474	0.076	0.067	0.093
0.534	0.016	0.005	0.031
0.475	0.076	0.076	0.076
0.609	0.059	0.033	0.103

**Table A70:** Set 1, trials 41–50 data (average actual PR values).

Test Avg	Avg Err	Min	Max
0.527	0.023	0.017	0.035
0.527	0.035	0.027	0.064
0.742	0.035	0.031	0.038
0.511	0.190	0.174	0.204
0.481	0.030	0.018	0.044
0.584	0.033	0.009	0.063
0.474	0.010	0.007	0.019
0.534	0.026	0.016	0.042
0.475	0.008	0.008	0.008
0.609	0.206	0.162	0.232

**Table A71:** Set 1, trials 41–50 data (single 0.5 PR value).

% Err Avg	% Max Avg	% Min Avg
47.4%	48.5%	44.8%
76.2%	213.8%	40.6%
49.5%	60.8%	41.4%
15.5%	48.8%	4.8%
67.5%	68.3%	54.3%
11.4%	46.6%	42.2%
16.5%	43.6%	8.6%
88.6%	90.9%	87.0%
13.1%	19.2%	10.1%
65.2%	75.2%	41.4%
45.1%	71.57%	37.52%

**Table A72:** Set 1, trials 41–50 data (actual PR value).

% Err Avg	% Max Avg	% Min Avg
46.2%	48.4%	45.6%
71.2%	194.8%	38.8%
46.3%	66.4%	31.8%
7.2%	23.0%	2.3%
67.5%	68.3%	54.3%
19.8%	112.4%	20.1%
16.5%	43.6%	8.6%
70.7%	69.6%	55.9%
13.1%	19.2%	10.1%
58.6%	82.0%	49.5%
41.71%	72.77%	31.70%

**Table A73:** Set 1, trials 41–50 data (all 0.5 PR values).

% Err Avg	% Max Avg	% Min Avg
46.37%	47.87%	43.10%
46.83%	43.00%	14.16%
362.63%	477.41%	268.19%
73.60%	75.61%	56.17%
137.21%	144.14%	112.63%
80.26%	215.29%	16.59%
151.06%	172.46%	126.96%
34.53%	51.28%	15.77%
150.91%	194.34%	103.07%
111.22%	251.53%	46.36%
119.46%	167.29%	80.30%

**Table A74:** Set 1, trials 41–50 data (average actual PR values).

% Err Avg	% Max Avg	% Min Avg
46.38%	47.87%	43.11%
71.24%	194.79%	38.80%
22.82%	28.50%	18.29%
129.59%	135.78%	126.24%
67.54%	68.32%	54.35%
78.36%	211.92%	15.35%
16.46%	43.63%	8.56%
55.92%	66.63%	45.06%
13.14%	19.16%	10.09%
81.61%	86.91%	69.16%
58.31%	90.35%	42.90%

**Table A75:** Set 1, trials 41–50 summary data (single 0.5 PR value).

Average Error	Avg Err	Lowest	Correct				False			
			At 0.01	At 0.025	At 0.05	At 0.1	At 0.01	At 0.025	At 0.05	At 0.10
0.050	0.024	N	N	Y	Y	Y	2	4	4	8
0.045	0.035	N	N	N	Y	Y	0	5	7	8
0.129	0.064	N	N	N	N	Y	0	0	0	2
0.064	0.010	N	N	Y	Y	Y	1	2	6	6
0.045	0.030	N	N	N	Y	Y	0	2	8	8
0.058	0.007	Y	Y	Y	Y	Y	1	2	2	9
0.063	0.010	N	N	Y	Y	Y	1	3	3	8
0.057	0.050	N	N	N	N	Y	2	3	4	8
0.061	0.008	N	Y	Y	Y	Y	2	3	3	8
0.153	0.100	N	N	N	N	Y	0	0	1	2

**Table A76:** Set 1, trials 41–50 summary data (actual PR value).

Average Error	Avg Err	Lowest	Correct				False			
			At 0.01	At 0.025	At 0.05	At 0.1	At 0.01	At 0.025	At 0.05	At 0.10
0.051	0.024	N	N	Y	Y	Y	2	4	4	8
0.049	0.035	N	N	N	Y	Y	0	4	7	8
0.149	0.069	N	N	N	N	Y	1	2	2	3
0.139	0.010	N	Y	Y	Y	Y	1	1	1	3
0.045	0.030	N	N	N	Y	Y	0	2	8	8
0.060	0.012	N	N	Y	Y	Y	1	1	4	8
0.063	0.010	N	N	Y	Y	Y	1	3	3	8
0.073	0.052	N	N	N	N	Y	1	2	3	6
0.061	0.008	N	Y	Y	Y	Y	2	3	3	8
0.301	0.176	N	N	N	N	N	0	0	0	1

**Table A77:** Set 1, trials 41–50 summary data (all 0.5 PR values).

Average Error	Avg Err	Lowest	Correct				False			
			At 0.01	At 0.025	At 0.05	At 0.1	At 0.01	At 0.025	At 0.05	At 0.10
0.050	0.023	N	N	Y	Y	Y	3	4	4	8
0.050	0.023	N	N	Y	Y	Y	2	4	4	8
0.053	0.192	N	N	N	N	N	1	4	6	9
0.053	0.039	N	N	N	Y	Y	1	4	5	8
0.050	0.069	N	N	N	N	Y	2	5	5	8
0.043	0.034	N	N	N	Y	Y	0	3	6	8
0.050	0.076	N	N	N	N	Y	2	5	5	8
0.045	0.016	N	N	Y	Y	Y	1	3	7	8
0.050	0.076	N	N	N	N	Y	2	5	5	8
0.053	0.059	N	N	N	N	Y	1	4	6	8

**Table A78:** Set 1, trials 41–50 summary data (average actual PR values).

Average Error	Avg Err	Lowest	Correct				False			
			At 0.01	At 0.025	At 0.05	At 0.1	At 0.01	At 0.025	At 0.05	At 0.10
0.050	0.023	N	N	Y	Y	Y	3	4	4	8
0.049	0.035	N	N	N	Y	Y	0	4	7	8
0.152	0.035	Y	N	N	Y	Y	0	0	0	2
0.147	0.190	N	N	N	N	N	0	0	1	3
0.045	0.030	N	N	N	Y	Y	0	2	8	8
0.043	0.033	N	N	N	Y	Y	0	3	6	8
0.063	0.010	N	N	Y	Y	Y	1	3	3	8
0.047	0.026	N	N	N	Y	Y	1	2	5	8
0.061	0.008	N	Y	Y	Y	Y	2	3	3	8
0.252	0.206	N	N	N	N	N	0	0	0	1

The second network design, shown in Figure 4, links heart rate and respiratory rate into a single rate rule while blood pressure and end-tidal carbon dioxide combine into another rule. These rules produce facts that serve as inputs to the final rule, which produces the output.

A similar set of tests was performed with this second network. Trials 1 through 10 utilized a default weight of 0.5 and the comprehensive data conversion method. Trials 11 through 20 used a default weight of 0.5 and the isolated data conversion method. Trials 21 through 50 utilized the comprehensive data conversion method with default weights of 0.6, 0.7, and 0.55 for trials 21 through 30, 31 through 40, and 41 through 50, respectively.

### Appendix B.2

#### Appendix B.2.1. Trials 1–10

Table A79, Table A80, Table A81, Table A82, Table A83, Table A84, Table A85, Table A86, Table A87, Table A88, Table A89, Table A90, Table A91, Table A92, Table A93 and Table A94 present data for trials 1 through 10 for set 2. The performance for these trials is similar to the first 10 trials of set 1, with the % ERR AVG equaling 44.57%. At an error margin of 0.05, nine target patients fell within the margin along with an average of 4.4 false patients. As seen in Table A94, at an error margin of 0.01 there are 4 correct patients included with an average of 0.7 incorrect patients as well. This equals a correct-to-incorrect patient ratio of 5.71. This is the highest ratio found in these ten trials across all PR approaches. The remaining approaches had their best ratios of 2.05 at an error margin of 0.05, 2.5 at 0.05, and 2.86 at 0.01 as shown in Table A91, Table A92 and Table A93, respectively.

**Table A79:** Set 2, trials 1–10 data (single 0.5 PR value).

Actual	Average	Average Error	Max Error	Min Error
0.515	0.527	0.038	0.055	0.025
0.498	0.526	0.039	0.056	0.027
0.644	0.482	0.167	0.194	0.140
0.434	0.506	0.072	0.093	0.055
0.540	0.565	0.049	0.065	0.037
0.515	0.482	0.069	0.095	0.052
0.497	0.484	0.057	0.083	0.051
0.782	0.482	0.299	0.326	0.272
0.493	0.527	0.042	0.058	0.031
0.500	0.482	0.060	0.086	0.051

**Table A80:** Set 2, trials 1–10 data (actual PR value).

Actual	Average	Average Error	Max Error	Min Error
0.500	0.552	0.054	0.080	0.045
0.500	0.550	0.054	0.080	0.044
0.644	0.482	0.167	0.194	0.140
0.500	0.519	0.043	0.068	0.037
0.499	0.611	0.113	0.145	0.098
0.515	0.482	0.069	0.095	0.052
0.500	0.485	0.058	0.084	0.051
0.782	0.482	0.299	0.326	0.272
0.500	0.552	0.552	0.577	0.528
0.500	0.482	0.060	0.086	0.051

**Table A81:** Set 2, trials 1–10 data (all 0.5 PR values).

Actual	Average	Average Error	Max Error	Min Error
0.500	0.527	0.039	0.056	0.026
0.500	0.526	0.039	0.056	0.026
0.500	0.482	0.060	0.086	0.051
0.500	0.506	0.043	0.063	0.031
0.500	0.565	0.074	0.089	0.060
0.500	0.482	0.060	0.086	0.051
0.500	0.484	0.058	0.084	0.050
0.500	0.482	0.060	0.086	0.051
0.500	0.527	0.039	0.056	0.026
0.500	0.527	0.039	0.056	0.026

**Table A82:** Set 2, trials 1–10 data (average actual PR values).

Actual	Average	Average Error	Max Error	Min Error
0.497	0.527	0.040	0.057	0.028
0.503	0.526	0.038	0.056	0.025
0.637	0.482	0.161	0.188	0.134
0.511	0.603	0.096	0.119	0.086
0.500	0.565	0.074	0.089	0.061
0.515	0.482	0.069	0.095	0.052
0.476	0.484	0.053	0.077	0.053
0.803	0.482	0.321	0.347	0.293
0.508	0.527	0.038	0.055	0.024
0.500	0.482	0.060	0.086	0.051

**Table A83:** Set 2, trials 1–10 data (single 0.5 PR value).

Test Avg	Avg Err	Min	Max
0.505	0.011	0.006	0.016
0.526	0.028	0.009	0.055
0.673	0.029	0.004	0.049
0.454	0.020	0.010	0.032
0.509	0.030	0.018	0.044
0.479	0.036	0.052	0.101
0.537	0.040	0.022	0.062
0.498	0.283	0.275	0.301
0.498	0.005	0.001	0.008
0.493	0.007	0.000	0.049

**Table A84:** Set 2, trials 1–10 data (actual PR value).

Test Avg	Avg Err	Min	Max
0.496	0.004	0.001	0.012
0.540	0.040	0.013	0.087
0.673	0.029	0.004	0.049
0.520	0.020	0.010	0.032
0.526	0.027	0.052	0.073
0.479	0.036	0.052	0.101
0.538	0.038	0.020	0.059
0.498	0.283	0.275	0.301
0.499	0.499	0.494	0.501
0.493	0.007	0.000	0.049

**Table A85:** Set 2, trials 1–10 data (all 0.5 PR values).

Test Avg	Avg Err	Min	Max
0.505	0.005	0.001	0.009
0.526	0.026	0.007	0.053
0.673	0.173	0.148	0.193
0.454	0.046	0.034	0.055
0.509	0.009	0.005	0.022
0.479	0.021	0.067	0.086
0.537	0.037	0.019	0.059
0.498	0.002	0.007	0.019
0.498	0.002	0.001	0.006
0.473	0.027	0.000	0.068

**Table A86:** Set 2, trials 1–10 data (average actual PR values).

Test Avg	Avg Err	Min	Max
0.505	0.008	0.002	0.012
0.526	0.022	0.003	0.049
0.673	0.037	0.012	0.056
0.523	0.012	0.001	0.031
0.509	0.010	0.004	0.023
0.479	0.036	0.052	0.101
0.537	0.061	0.043	0.083
0.498	0.305	0.296	0.322
0.498	0.010	0.007	0.014
0.493	0.007	0.000	0.049

**Table A87:** Set 2, trials 1–10 data (single 0.5 PR value).

% Err Avg	% Max Avg	% Min Avg
27.70%	29.53%	23.87%
70.90%	202.50%	15.41%
17.50%	34.72%	2.07%
27.42%	57.86%	11.10%
61.63%	67.74%	46.98%
52.08%	105.62%	99.33%
70.41%	121.02%	26.55%
94.78%	100.94%	92.22%
11.62%	24.74%	1.18%
11.63%	56.30%	0.00%
44.57%	80.10%	31.87%

**Table A88:** Set 2, trials 1–10 data (actual PR value).

% Err Avg	% Max Avg	% Min Avg
7.69%	14.97%	1.82%
74.54%	197.27%	16.44%
17.50%	34.72%	2.07%
45.34%	86.74%	15.32%
24.17%	73.72%	35.86%
52.08%	105.62%	99.33%
65.84%	117.44%	23.66%
94.78%	100.94%	92.22%
90.34%	94.90%	85.62%
11.63%	56.30%	0.00%
48.39%	88.26%	37.23%

**Table A89:** Set 2, trials 1–10 data (all 0.5 PR values).

% Err Avg	% Max Avg	% Min Avg
12.00%	36.23%	1.83%
66.56%	203.43%	12.00%
290.89%	379.31%	171.79%
106.19%	107.20%	87.30%
12.65%	36.61%	5.08%
34.77%	132.02%	99.25%
63.55%	116.53%	22.44%
3.24%	22.05%	13.79%
5.03%	10.89%	3.02%
69.02%	121.74%	0.00%
66.39%	116.60%	41.65%

**Table A90:** Set 2, trials 1–10 data (average actual PR values).

% Err Avg	% Max Avg	% Min Avg
19.00%	43.77%	3.26%
58.30%	199.51%	6.00%
22.79%	41.88%	6.13%
12.48%	35.57%	0.84%
13.26%	37.19%	4.49%
52.08%	105.62%	99.33%
115.35%	156.04%	55.70%
95.13%	100.87%	92.70%
25.98%	30.27%	25.49%
11.63%	56.30%	0.00%
42.60%	80.70%	29.39%

**Table A91:** Set 2, trials 1–10 summary data (single 0.5 PR value).

Average Error	Avg Err	Lowest	Correct				False			
			At 0.01	At 0.025	At 0.05	At 0.1	At 0.01	At 0.025	At 0.05	At 0.10
0.038	0.011	Y	N	Y	Y	Y	0	4	7	8
0.039	0.028	N	N	N	Y	Y	2	5	7	8
0.167	0.029	Y	N	N	Y	Y	0	0	0	0
0.072	0.020	N	N	Y	Y	Y	1	1	4	6
0.049	0.030	N	N	N	Y	Y	3	4	5	8
0.069	0.036	N	N	N	Y	Y	0	3	3	7
0.057	0.040	N	N	N	Y	Y	2	3	6	7
0.299	0.283	N	N	N	N	N	0	0	0	0
0.042	0.005	Y	Y	Y	Y	Y	2	4	7	7
0.060	0.007	N	Y	Y	Y	Y	1	2	5	7

**Table A92:** Set 2, trials 1–10 summary data (actual PR value).

Average Error	Avg Err	Lowest	Correct				False			
			At 0.01	At 0.025	At 0.05	At 0.1	At 0.01	At 0.025	At 0.05	At 0.10
0.054	0.004	N	Y	Y	Y	Y	2	3	5	7
0.054	0.040	N	N	N	Y	Y	3	4	5	7
0.167	0.029	Y	N	N	Y	Y	0	0	0	0
0.043	0.020	N	N	Y	Y	Y	2	4	6	8
0.113	0.027	Y	N	N	Y	Y	0	0	2	4
0.069	0.036	N	N	N	Y	Y	0	3	3	7
0.058	0.038	N	N	N	Y	Y	2	3	6	7
0.299	0.283	N	N	N	N	N	0	0	0	0
0.552	0.499	N	N	N	N	N	0	0	0	0
0.060	0.007	N	Y	Y	Y	Y	1	2	5	7

**Table A93:** Set 2, trials 1–10 summary data (all 0.5 PR values).

Average Error	Avg Err	Lowest	Correct				False			
			At 0.01	At 0.025	At 0.05	At 0.1	At 0.01	At 0.025	At 0.05	At 0.10
0.039	0.005	N	Y	Y	Y	Y	1	3	7	7
0.039	0.026	N	N	N	Y	Y	2	4	7	8
0.060	0.173	N	N	N	N	N	2	3	6	8
0.043	0.046	N	N	N	Y	Y	1	4	7	8
0.074	0.009	Y	Y	Y	Y	Y	0	0	4	7
0.060	0.021	N	N	Y	Y	Y	2	2	5	7
0.058	0.037	N	N	N	Y	Y	2	3	6	7
0.060	0.002	Y	Y	Y	Y	Y	1	2	5	7
0.039	0.002	Y	Y	Y	Y	Y	1	3	7	7
0.039	0.027	N	N	N	Y	Y	2	4	7	7

**Table A94:** Set 2, trials 1–10 summary data (average actual PR values).

Average Error	Avg Err	Lowest	Correct				False			
			At 0.01	At 0.025	At 0.05	At 0.1	At 0.01	At 0.025	At 0.05	At 0.10
0.040	0.008	N	Y	Y	Y	Y	1	4	7	7
0.038	0.022	N	N	Y	Y	Y	2	5	7	8
0.161	0.037	Y	N	N	Y	Y	0	0	0	1
0.096	0.012	Y	N	Y	Y	Y	0	1	4	6
0.074	0.010	Y	Y	Y	Y	Y	0	0	4	7
0.069	0.036	N	N	N	Y	Y	0	3	3	7
0.053	0.061	N	N	N	N	Y	2	5	6	7
0.321	0.305	N	N	N	N	N	0	0	0	0
0.038	0.010	N	Y	Y	Y	Y	1	5	7	8
0.060	0.007	N	Y	Y	Y	Y	1	2	5	7

#### Appendix B.2.2. Trials 11–20

Data for trials 11 through 20 of set 2 is presented in Table A95, Table A96, Table A97, Table A98, Table A99, Table A100, Table A101, Table A102, Table A103, Table A104, Table A105, Table A106, Table A107, Table A108 and Table A109. The % ERR AVG is higher than many other sets of trials at 82.86%. This indicates that the target patient average margin of error is only slightly lower than the overall average error. At a 0.05 margin, five of the ten target patients fall within the range with an average of 4.1 additional incorrect patients also having error levels within this range. Increasing the margin to 0.1 increases the number of target patients falling within the margin to nine while also increasing the average number of erroneously identified patients to 6.8. Table 3 shows that, in that case, no patients, correct or incorrect, fell within even a 0.1 margin of error. The highest correct-to-incorrect ratio found in these trials was 3.33, which was achieved with both the first and second PR approach at an error margin of 0.01. The fourth approach, presented in Table A109 yielded a ratio of 2.5 at an error margin of 0.025. As shown in Table 3, no patients, correct or incorrect, fell within even a 0.1 margin of error, for the actual PR value approach.

**Table A95:** Set 2, trials 11–20 data (single 0.5 PR value).

Actual	Average	Average Error	Max Error	Min Error
0.900	0.923	0.048	0.091	0.039
0.809	0.926	0.117	0.168	0.077
0.894	0.923	0.050	0.095	0.042
0.809	0.927	0.119	0.168	0.080
0.934	0.912	0.050	0.101	0.027
0.894	0.934	0.054	0.101	0.045
1.000	0.922	0.078	0.124	0.025
0.932	0.923	0.042	0.084	0.024
0.956	0.933	0.032	0.073	0.021
0.934	0.923	0.042	0.084	0.024

**Table A96:** Set 2, trials 11–20 data (actual PR value).

Actual	Average	Average Error	Max Error	Min Error
0.900	0.923	0.048	0.091	0.039
0.809	0.922	0.114	0.166	0.084
0.894	0.923	0.050	0.095	0.042
0.894	0.923	0.050	0.095	0.042
0.934	0.923	0.042	0.084	0.024
0.894	0.923	0.050	0.094	0.042
0.894	0.923	0.050	0.094	0.042
1.000	0.923	0.077	0.121	0.025
0.956	0.923	0.042	0.084	0.025
0.934	0.923	0.042	0.084	0.024

**Table A97:** Set 2, trials 11–20 data (all 0.5 PR values).

Actual	Average	Average Error	Max Error	Min Error
0.500	0.902	0.402	0.468	0.354
0.500	0.923	0.423	0.475	0.380
0.500	0.923	0.423	0.475	0.380
0.500	0.923	0.423	0.475	0.380
0.500	0.923	0.423	0.475	0.380
0.500	0.923	0.423	0.475	0.380
0.500	0.923	0.423	0.475	0.380
0.500	0.923	0.423	0.475	0.380
0.500	0.923	0.423	0.475	0.380
0.500	0.923	0.423	0.475	0.380

**Table A98:** Set 2, trials 11–20 data (average actual PR values).

Actual	Average	Average Error	Max Error	Min Error
0.600	0.923	0.324	0.375	0.280
0.642	0.923	0.282	0.333	0.238
0.861	0.923	0.068	0.116	0.060
0.606	0.923	0.317	0.369	0.274
0.694	0.923	0.229	0.281	0.186
0.654	0.923	0.270	0.321	0.226
0.826	0.923	0.098	0.149	0.077
0.803	0.923	0.120	0.172	0.091
0.781	0.923	0.142	0.194	0.109
0.875	0.923	0.060	0.106	0.051

**Table A99:** Set 2, trials 11–20 data (single 0.5 PR value).

Test Avg	Avg Err	Min	Max
0.913	0.013	0.028	0.036
0.884	0.075	0.002	0.180
0.957	0.063	0.012	0.103
0.968	0.160	0.133	0.192
0.918	0.016	0.054	0.060
0.834	0.061	0.078	0.164
0.966	0.034	0.005	0.059
0.976	0.044	0.013	0.061
0.958	0.002	0.013	0.013
0.861	0.073	0.026	0.102

**Table A100:** Set 2, trials 11–20 data (actual PR value).

Test Avg	Avg Err	Min	Max
0.913	0.013	0.028	0.036
0.884	0.075	0.002	0.180
0.957	0.063	0.012	0.103
0.968	0.075	0.048	0.107
0.918	0.016	0.054	0.060
0.834	0.061	0.078	0.164
0.966	0.072	0.047	0.101
0.976	0.024	0.007	0.055
0.958	0.002	0.013	0.013
0.861	0.073	0.026	0.102

**Table A101:** Set 2, trials 11–20 data (all 0.5 PR values).

Test Avg	Avg Err	Min	Max
0.907	0.407	0.371	0.483
0.884	0.384	0.307	0.488
0.957	0.457	0.405	0.496
0.968	0.468	0.441	0.500
0.918	0.418	0.380	0.494
0.834	0.334	0.230	0.472
0.966	0.466	0.441	0.495
0.976	0.476	0.445	0.493
0.958	0.458	0.443	0.468
0.861	0.361	0.332	0.408

**Table A102:** Set 2, trials 11–20 data (average actual PR values).

Test Avg	Avg Err	Min	Max
0.913	0.313	0.273	0.337
0.884	0.242	0.165	0.347
0.957	0.096	0.045	0.136
0.968	0.362	0.335	0.394
0.918	0.224	0.186	0.300
0.834	0.180	0.077	0.319
0.966	0.140	0.116	0.170
0.976	0.173	0.142	0.190
0.958	0.177	0.162	0.187
0.861	0.014	0.033	0.043

**Table A103:** Set 2, trials 11–20 data (single 0.5 PR value).

% Err Avg	% Max Avg	% Min Avg
26.87%	92.19%	30.67%
64.04%	234.03%	1.19%
125.74%	242.89%	12.15%
134.79%	238.48%	78.66%
31.61%	220.37%	53.73%
112.48%	174.30%	162.14%
44.10%	47.54%	19.65%
106.65%	249.49%	15.46%
7.28%	59.10%	17.77%
175.07%	121.12%	106.92%
82.86%	167.95%	49.83%

**Table A104:** Set 2, trials 11–20 data (actual PR value).

% Err Avg	% Max Avg	% Min Avg
26.87%	92.19%	30.67%
66.12%	214.97%	1.20%
125.74%	242.89%	12.15%
148.40%	252.37%	50.18%
38.24%	251.59%	64.52%
120.42%	185.71%	173.82%
142.74%	240.48%	49.81%
30.77%	45.64%	27.94%
5.66%	50.71%	15.44%
175.07%	121.12%	106.92%
88.00%	169.77%	53.27%

**Table A105:** Set 2, trials 11–20 data (all 0.5 PR values).

% Err Avg	% Max Avg	% Min Avg
101.24%	136.34%	79.20%
90.63%	128.59%	64.53%
107.92%	130.70%	85.27%
110.62%	131.75%	92.85%
98.74%	130.04%	80.01%
78.77%	124.37%	48.43%
109.99%	130.43%	92.85%
112.52%	129.91%	93.69%
108.14%	123.32%	93.17%
85.16%	107.51%	69.90%
100.37%	127.30%	79.99%

**Table A106:** Set 2, trials 11–20 data (average actual PR values).

% Err Avg	% Max Avg	% Min Avg
96.75%	120.18%	72.58%
85.92%	145.59%	49.48%
141.18%	226.97%	38.25%
114.16%	144.06%	90.80%
97.68%	161.46%	66.20%
66.69%	140.93%	23.80%
143.22%	220.42%	77.28%
144.04%	208.56%	82.58%
124.20%	172.03%	83.27%
24.21%	65.22%	40.66%
103.81%	160.54%	62.49%

**Table A107:** Set 2, trials 11–20 data (single 0.5 PR value).

Average Error	Avg Err	Lowest	Correct				False			
			At 0.01	At 0.025	At 0.05	At 0.1	At 0.01	At 0.025	At 0.05	At 0.10
0.048	0.013	Y	N	Y	Y	Y	0	2	3	9
0.117	0.075	N	N	N	N	Y	0	0	0	1
0.050	0.063	N	N	N	N	Y	1	3	4	9
0.119	0.160	N	N	N	N	N	0	0	0	2
0.050	0.016	Y	N	Y	Y	Y	0	2	5	7
0.054	0.061	N	N	N	N	Y	0	3	3	9
0.078	0.034	N	N	N	Y	Y	0	1	4	6
0.042	0.044	N	N	N	Y	Y	0	3	7	9
0.032	0.002	N	Y	Y	Y	Y	2	5	7	8
0.042	0.073	N	N	N	N	Y	0	4	8	8

**Table A108:** Set 2, trials 11–20 data (actual PR value).

Average Error	Avg Err	Lowest	Correct				False			
			At 0.01	At 0.025	At 0.05	At 0.1	At 0.01	At 0.025	At 0.05	At 0.10
0.048	0.013	Y	N	Y	Y	Y	0	2	3	9
0.114	0.075	N	N	N	N	Y	0	0	1	2
0.050	0.063	N	N	N	N	Y	1	3	4	9
0.050	0.075	N	N	N	N	Y	1	3	4	9
0.042	0.016	Y	N	Y	Y	Y	0	3	6	8
0.050	0.061	N	N	N	N	Y	0	3	4	9
0.050	0.072	N	N	N	N	Y	0	3	4	9
0.077	0.024	Y	N	Y	Y	Y	0	0	4	6
0.042	0.002	N	Y	Y	Y	Y	1	4	6	8
0.042	0.073	N	N	N	N	Y	0	4	8	8

**Table A109:** Set 2, trials 11–20 data (average actual PR values).

Average Error	Avg Err	Lowest	Correct				False			
			At 0.01	At 0.025	At 0.05	At 0.1	At 0.01	At 0.025	At 0.05	At 0.10
0.324	0.313	N	N	N	N	N	0	0	0	0
0.282	0.242	N	N	N	N	N	0	0	0	0
0.068	0.096	N	N	N	N	Y	1	2	3	6
0.317	0.362	N	N	N	N	N	0	0	0	0
0.229	0.224	N	N	N	N	N	0	0	0	0
0.270	0.180	Y	N	N	N	N	0	0	0	0
0.098	0.140	N	N	N	N	N	1	1	2	5
0.120	0.173	N	N	N	N	N	0	0	1	3
0.142	0.177	N	N	N	N	N	0	0	0	2
0.060	0.014	N	N	Y	Y	Y	1	1	4	8

#### Appendix B.2.3. Trials 21–30

Data for trials 21 through 30, using the second GDES network, are presented in Table A110, Table A111, Table A112, Table A113, Table A114, Table A115, Table A116, Table A117, Table A118, Table A119, Table A120, Table A121, Table A122, Table A123 and Table A124. The % err avg is 45.5, which is quite similar to many of the previous trials. At an error margin of 0.025, five of the target patients were identified while only an average of 1.6 incorrect patients were also incorrectly identified. Expanding the margin to 0.05 allows nine of the target patients to be identified with an average of 2.8 incorrect patients falling within the margin. As seen in Table 4, at an error margin of 0.025, five correct patients fall within the margin while an average of 1.2 incorrect patients are identified. This leads to a correct-to-incorrect ratio of 4.17. This is the highest ratio of these trials. The highest ratios from the other PR value, as shown in Table A122, Table A123 and Table A124, were 3.21 at 0.05, 1.79 at 0.05, and 1.67 at 0.025, respectively.

**Table A110:** Set 2, trials 21–30 data (single 0.5 PR value).

Actual	Average	Average Error	Max Error	Min Error
0.671	0.610	0.073	0.082	0.066
0.607	0.616	0.049	0.066	0.033
0.644	0.491	0.159	0.194	0.130
0.484	0.541	0.058	0.075	0.041
0.590	0.616	0.047	0.065	0.034
0.651	0.565	0.088	0.104	0.074
0.545	0.527	0.044	0.061	0.035
0.782	0.482	0.299	0.326	0.272
0.563	0.616	0.063	0.079	0.054
0.600	0.491	0.124	0.158	0.095

**Table A111:** Set 2, trials 21–30 data (actual PR value).

Actual	Average	Average Error	Max Error	Min Error
0.600	0.608	0.050	0.089	0.050
0.561	0.620	0.072	0.114	0.059
0.644	0.491	0.159	0.194	0.130
0.600	0.562	0.055	0.083	0.047
0.499	0.620	0.121	0.160	0.097
0.598	0.567	0.053	0.082	0.044
0.600	0.544	0.071	0.094	0.057
0.414	0.620	0.206	0.245	0.172
0.956	0.923	0.042	0.084	0.025
0.600	0.491	0.124	0.158	0.095

**Table A112:** Set 2, trials 21–30 data (all 0.5 PR values).

Actual	Average	Average Error	Max Error	Min Error
0.600	0.605	0.045	0.061	0.031
0.600	0.616	0.047	0.065	0.033
0.600	0.491	0.124	0.158	0.095
0.600	0.560	0.058	0.075	0.040
0.600	0.616	0.047	0.065	0.033
0.600	0.565	0.055	0.072	0.038
0.600	0.542	0.070	0.089	0.057
0.600	0.616	0.047	0.065	0.033
0.600	0.616	0.047	0.065	0.033
0.600	0.491	0.124	0.158	0.095

**Table A113:** Set 2, trials 21–30 data (average actual PR values).

Actual	Average	Average Error	Max Error	Min Error
0.601	0.605	0.045	0.061	0.031
0.559	0.616	0.065	0.081	0.058
0.637	0.482	0.161	0.188	0.134
0.599	0.541	0.060	0.077	0.054
0.500	0.616	0.117	0.131	0.112
0.599	0.565	0.055	0.071	0.038
0.541	0.542	0.040	0.059	0.032
0.803	0.482	0.321	0.347	0.293
0.528	0.616	0.090	0.106	0.086
0.600	0.491	0.124	0.158	0.095

**Table A114:** Set 2, trials 21–30 data (single 0.5 PR value).

Test Avg	Avg Err	Min	Max
0.654	0.017	0.015	0.024
0.631	0.024	0.009	0.027
0.673	0.029	0.004	0.049
0.498	0.015	0.010	0.021
0.559	0.030	0.018	0.044
0.641	0.011	0.022	0.037
0.497	0.048	0.018	0.066
0.498	0.283	0.275	0.301
0.590	0.027	0.027	0.027
0.579	0.021	0.000	0.149

**Table A115:** Set 2, trials 21–30 data (actual PR value).

Test Avg	Avg Err	Min	Max
0.594	0.006	0.001	0.010
0.616	0.055	0.035	0.112
0.673	0.029	0.004	0.049
0.611	0.011	0.005	0.020
0.526	0.027	0.052	0.073
0.607	0.009	0.022	0.063
0.560	0.040	0.019	0.060
0.528	0.115	0.098	0.136
0.958	0.002	0.013	0.013
0.579	0.021	0.000	0.149

**Table A116:** Set 2, trials 21–30 data (all 0.5 PR values).

Test Avg	Avg Err	Min	Max
0.654	0.054	0.048	0.056
0.631	0.031	0.016	0.034
0.673	0.073	0.048	0.093
0.513	0.088	0.079	0.094
0.559	0.041	0.028	0.055
0.641	0.040	0.014	0.073
0.558	0.042	0.029	0.053
0.601	0.001	0.011	0.016
0.590	0.011	0.011	0.011
0.579	0.021	0.000	0.149

**Table A117:** Set 2, trials 21–30 data (average actual PR values).

Test Avg	Avg Err	Min	Max
0.654	0.053	0.047	0.055
0.631	0.072	0.057	0.074
0.673	0.037	0.012	0.056
0.498	0.100	0.094	0.105
0.559	0.060	0.046	0.073
0.641	0.041	0.015	0.073
0.558	0.018	0.006	0.031
0.498	0.305	0.296	0.322
0.590	0.062	0.062	0.062
0.579	0.021	0.000	0.149

**Table A118:** Set 2, trials 21–30 data (single 0.5 PR value).

% Err Avg	% Max Avg	% Min Avg
23.53%	28.84%	22.86%
49.26%	79.82%	13.68%
18.44%	37.39%	2.07%
25.34%	51.30%	13.33%
63.49%	67.74%	50.95%
11.96%	35.38%	29.03%
108.88%	108.15%	51.49%
94.78%	100.94%	92.22%
42.16%	49.21%	33.52%
17.16%	93.75%	0.00%
45.50%	65.25%	30.92%

**Table A119:** Set 2, trials 21–30 data (actual PR value).

% Err Avg	% Max Avg	%Min Avg
12.55%	11.27%	2.08%
75.76%	187.87%	30.78%
18.44%	37.39%	2.07%
20.85%	42.62%	5.99%
22.47%	74.47%	32.58%
16.27%	143.76%	27.33%
56.22%	63.03%	32.43%
55.46%	79.09%	39.86%
5.66%	50.71%	15.44%
17.16%	93.75%	0.00%
30.08%	78.40%	18.86%

**Table A120:** Set 2, trials 21–30 data (all 0.5 PR values).

% Err Avg	% Max Avg	%Min Avg
120.07%	182.60%	77.63%
65.29%	102.60%	24.63%
59.29%	97.88%	30.30%
151.10%	198.30%	125.96%
85.60%	85.76%	83.91%
72.73%	191.54%	19.58%
59.16%	59.99%	50.07%
1.65%	49.00%	16.17%
22.12%	32.16%	16.17%
17.16%	93.75%	0.00%
65.42%	109.36%	44.44%

**Table A121:** Set 2, trials 21–30 data (average actual PR values).

% Err Avg	% Max Avg	% Min Avg
118.18%	180.36%	76.33%
110.22%	128.78%	69.98%
22.79%	41.88%	6.13%
165.71%	174.77%	136.56%
51.16%	64.56%	35.01%
74.93%	195.50%	20.89%
44.33%	94.64%	10.15%
95.13%	100.87%	92.70%
68.24%	71.35%	57.91%
17.16%	93.75%	0.00%
76.79%	114.65%	50.57%

**Table A122:** Set 2, trials 21–30 summary data (single 0.5 PR value).

Average Error	Avg Err	Lowest	Correct				False			
			At 0.01	At 0.025	At 0.05	At 0.1	At 0.01	At 0.025	At 0.05	At 0.10
0.073	0.017	Y	N	Y	Y	Y	0	1	1	8
0.049	0.024	N	N	Y	Y	Y	1	4	6	8
0.159	0.029	Y	N	N	Y	Y	0	0	0	1
0.058	0.015	N	N	Y	Y	Y	0	1	4	7
0.047	0.030	N	N	N	Y	Y	3	4	5	8
0.088	0.011	N	N	Y	Y	Y	1	1	1	4
0.044	0.048	N	N	N	Y	Y	2	3	6	8
0.299	0.283	N	N	N	N	N	0	0	0	0
0.063	0.027	N	N	N	Y	Y	1	2	5	7
0.124	0.021	Y	N	Y	Y	Y	0	0	0	2

**Table A123:** Set 2, trials 21–30 summary data (all 0.5 PR values).

Average Error	Avg Err	Lowest	Correct				False			
			At 0.01	At 0.025	At 0.05	At 0.1	At 0.01	At 0.025	At 0.05	At 0.10
0.045	0.054	N	N	N	N	Y	1	4	5	8
0.047	0.031	N	N	N	Y	Y	1	4	6	8
0.124	0.073	N	N	N	N	Y	0	1	1	2
0.058	0.088	N	N	N	N	Y	0	0	4	9
0.047	0.041	N	N	N	Y	Y	1	4	6	8
0.055	0.040	N	N	N	Y	Y	0	0	3	9
0.070	0.042	N	N	N	Y	Y	1	1	2	6
0.047	0.001	Y	Y	Y	Y	Y	0	3	6	8
0.047	0.011	N	N	Y	Y	Y	1	3	6	8
0.124	0.021	Y	N	Y	Y	Y	0	0	0	2

**Table A124:** Set 2, trials 21–30 summary data (average actual PR values).

Average Error	Avg Err	Lowest	Correct				False			
			At 0.01	At 0.025	At 0.05	At 0.1	At 0.01	At 0.025	At 0.05	At 0.10
0.045	0.053	N	N	N	N	Y	1	4	5	8
0.065	0.072	N	N	N	N	Y	1	1	6	7
0.161	0.037	Y	N	N	Y	Y	0	0	0	1
0.060	0.100	N	N	N	N	N	1	2	3	8
0.117	0.060	N	N	N	N	Y	0	1	1	4
0.055	0.041	N	N	N	Y	Y	0	0	3	9
0.040	0.018	N	N	Y	Y	Y	2	3	5	8
0.321	0.305	N	N	N	N	N	0	0	0	0
0.090	0.062	N	N	N	N	Y	1	1	2	5
0.124	0.021	Y	N	Y	Y	Y	0	0	0	2

#### Appendix B.2.4. Trials 31–40

Table A125, Table A126, Table A127, Table A128, Table A129, Table A130, Table A131, Table A132, Table A133, Table A134, Table A135, Table A136, Table A137, Table A138, Table A139 and Table A140 present the data from trials 31 through 40 for the second GDES network. Roughly similar to most other trials, the % err avg for this set was 45.71%. Additionally, similar to the previous ten trials, an error margin of 0.025 yielded five identified target patients with 1.7 incorrect patients also identified, on average. Expanding the error margin to 0.05 yielded 8 target patients identified and an average of 3.2 incorrect patients also being identified. This is shown in Table A137. The highest correct-to-incorrect ratio is thus 2.94 at 0.025. This is a greater ratio than any found from the results of Table A138, Table A139 and Table A140, which have ratios of 2.5 at 0.01, 1.25 at 0.025, and 0.48 at 0.025, respectively.

**Table A125:** Set 2, trials 31–40 data (single 0.5 PR value).

Actual	Average	Average Error	Max Error	Min Error
0.754	0.668	0.096	0.118	0.078
0.657	0.668	0.047	0.066	0.037
0.666	0.668	0.049	0.068	0.035
0.517	0.562	0.050	0.067	0.026
0.640	0.668	0.046	0.065	0.039
0.798	0.668	0.131	0.153	0.115
0.632	0.635	0.038	0.055	0.035
0.534	0.668	0.134	0.152	0.125
0.613	0.668	0.061	0.079	0.059
0.508	0.668	0.160	0.178	0.146

**Table A126:** Set 2, trials 31–40 data (actual PR value).

Actual	Average	Average Error	Max Error	Min Error
0.625	0.629	0.052	0.104	0.050
0.561	0.627	0.080	0.130	0.056
0.636	0.629	0.054	0.104	0.050
0.700	0.605	0.096	0.129	0.079
0.499	0.629	0.130	0.176	0.100
0.657	0.629	0.063	0.109	0.052
0.700	0.603	0.097	0.130	0.080
0.414	0.629	0.215	0.261	0.175
0.526	0.629	0.103	0.152	0.078
0.613	0.629	0.053	0.105	0.051

**Table A127:** Set 2, trials 31–40 data (all 0.5 PR values).

Actual	Average	Average Error	Max Error	Min Error
0.700	0.668	0.060	0.081	0.042
0.700	0.668	0.060	0.081	0.042
0.700	0.668	0.060	0.081	0.042
0.700	0.637	0.072	0.089	0.054
0.700	0.668	0.060	0.081	0.042
0.700	0.668	0.060	0.081	0.042
0.700	0.635	0.074	0.090	0.056
0.700	0.668	0.060	0.081	0.042
0.700	0.668	0.060	0.081	0.042
0.700	0.668	0.060	0.081	0.042

**Table A128:** Set 2, trials 31–40 data (average actual PR value).

Actual	Average	Average Error	Max Error	Min Error
0.627	0.668	0.053	0.071	0.047
0.559	0.668	0.109	0.127	0.105
0.628	0.668	0.053	0.070	0.047
0.646	0.562	0.091	0.109	0.083
0.500	0.668	0.168	0.186	0.152
0.659	0.668	0.048	0.066	0.036
0.606	0.635	0.038	0.055	0.038
0.407	0.668	0.261	0.279	0.240
0.528	0.668	0.140	0.158	0.130
0.700	0.668	0.060	0.081	0.042

**Table A129:** Set 2, trials 31–40 data (single 0.5 PR value).

Test Avg	Avg Err	Min	Max
0.735	0.019	0.018	0.027
0.681	0.024	0.009	0.027
0.699	0.033	0.018	0.035
0.535	0.019	0.019	0.019
0.609	0.030	0.018	0.044
0.805	0.007	0.009	0.026
0.619	0.013	0.008	0.029
0.651	0.117	0.106	0.132
0.640	0.027	0.027	0.027
0.584	0.076	0.035	0.192

**Table A130:** Set 2, trials 31–40 data (actual PR value).

Test Avg	Avg Err	Min	Max
0.618	0.007	0.001	0.011
0.623	0.062	0.035	0.123
0.673	0.038	0.014	0.052
0.703	0.003	0.003	0.008
0.526	0.027	0.052	0.073
0.704	0.047	0.020	0.150
0.588	0.112	0.078	0.149
0.528	0.115	0.098	0.136
0.612	0.087	0.018	0.114
0.623	0.010	0.080	0.155

**Table A131:** Set 2, trials 31–40 data (all 0.5 PR values).

Test Avg	Avg Err	Min	Max
0.735	0.035	0.027	0.036
0.681	0.019	0.017	0.034
0.699	0.001	0.001	0.017
0.597	0.103	0.098	0.109
0.609	0.091	0.078	0.105
0.805	0.105	0.089	0.124
0.619	0.081	0.076	0.096
0.651	0.049	0.034	0.061
0.640	0.061	0.061	0.061
0.584	0.116	0.000	0.227

**Table A132:** Set 2, trials 31–40 data (average actual PR values).

Test Avg	Avg Err	Min	Max
0.735	0.108	0.101	0.110
0.681	0.122	0.107	0.125
0.699	0.071	0.056	0.073
0.535	0.111	0.111	0.111
0.609	0.110	0.096	0.123
0.805	0.146	0.130	0.165
0.619	0.013	0.002	0.019
0.651	0.244	0.233	0.259
0.640	0.112	0.112	0.112
0.584	0.116	0.000	0.227

**Table A133:** Set 2, trials 31–40 data (single 0.5 PR value).

% Err Avg	% Max Avg	% Min Avg
19.60%	22.55%	22.41%
50.75%	72.01%	13.68%
66.20%	100.00%	25.89%
37.12%	71.43%	27.82%
65.46%	67.74%	44.47%
5.61%	22.59%	5.89%
34.42%	52.17%	22.37%
87.20%	105.85%	69.45%
43.14%	45.03%	33.52%
47.61%	131.96%	19.67%
45.71%	69.13%	28.52%

**Table A134:** Set 2, trials 31–40 data (actual PR value).

% Err Avg	% Max Avg	% Min Avg
13.06%	10.08%	3.02%
77.25%	217.78%	26.96%
69.69%	103.73%	12.96%
3.29%	10.41%	2.55%
20.99%	72.18%	29.60%
75.38%	285.85%	17.93%
115.40%	114.41%	97.74%
53.25%	77.69%	37.40%
84.04%	145.41%	11.84%
19.27%	304.82%	75.90%
53.16%	134.24%	31.59%

**Table A135:** Set 2, trials 31–40 data (all 0.5 PR values).

% Err Avg	% Max Avg	% Min Avg
57.86%	86.12%	33.33%
31.67%	41.98%	39.47%
2.50%	20.37%	2.39%
142.71%	179.84%	123.03%
151.09%	186.60%	129.01%
174.78%	295.45%	109.26%
110.03%	135.34%	106.92%
82.03%	81.34%	74.69%
100.85%	144.74%	74.69%
193.12%	280.25%	0.00%
104.66%	145.20%	69.28%

**Table A136:** Set 2, trials 31–40 data (average actual PR values).

% Err Avg	% Max Avg	% Min Avg
202.95%	232.24%	141.65%
112.01%	118.91%	84.32%
134.67%	157.89%	79.60%
122.65%	133.49%	101.69%
65.23%	80.43%	51.50%
305.83%	451.92%	195.62%
34.84%	48.80%	3.73%
93.43%	108.14%	83.36%
79.69%	86.10%	70.61%
193.12%	280.25%	0.00%
134.44%	169.82%	81.21%

**Table A137:** Set 2, trials 31–40 summary data (single 0.5 PR value).

Average Error	Avg Err	Lowest	Correct				False			
			At 0.01	At 0.025	At 0.05	At 0.1	At 0.01	At 0.025	At 0.05	At 0.10
0.096	0.019	Y	N	Y	Y	Y	0	0	0	3
0.047	0.024	N	N	Y	Y	Y	1	4	6	8
0.049	0.033	N	N	N	Y	Y	0	2	5	8
0.050	0.019	Y	N	Y	Y	Y	0	3	5	8
0.046	0.030	N	N	N	Y	Y	3	4	5	8
0.131	0.007	Y	Y	Y	Y	Y	0	0	0	2
0.038	0.013	N	N	Y	Y	Y	1	2	6	8
0.134	0.117	N	N	N	N	N	0	0	0	2
0.061	0.027	N	N	N	Y	Y	1	2	5	7
0.160	0.076	Y	N	N	N	Y	0	0	0	0

**Table A138:** Set 2, trials 31–40 summary data (actual PR value).

Average Error	Avg Err	Lowest	Correct				False			
			At 0.01	At 0.025	At 0.05	At 0.1	At 0.01	At 0.025	At 0.05	At 0.10
0.052	0.007	N	Y	Y	Y	Y	2	4	5	8
0.080	0.062	N	N	N	N	Y	0	0	3	6
0.054	0.038	N	N	N	Y	Y	1	4	5	6
0.096	0.003	Y	Y	Y	Y	Y	0	0	2	4
0.130	0.027	Y	N	N	Y	Y	0	0	1	1
0.063	0.047	N	N	N	Y	Y	0	2	5	6
0.097	0.112	N	N	N	N	N	1	1	3	5
0.215	0.115	N	N	N	N	N	0	0	0	0
0.103	0.087	N	N	N	N	Y	2	2	2	5
0.053	0.010	N	N	Y	Y	Y	2	4	4	8

**Table A139:** Set 2, trials 31–40 summary data (all 0.5 PR values).

Average Error	Avg Err	Lowest	Correct				False			
			At 0.01	At 0.025	At 0.05	At 0.1	At 0.01	At 0.025	At 0.05	At 0.10
0.060	0.035	N	N	N	Y	Y	1	2	3	7
0.060	0.019	N	N	Y	Y	Y	1	1	3	7
0.060	0.001	Y	Y	Y	Y	Y	0	1	3	7
0.072	0.103	N	N	N	N	N	1	1	3	7
0.060	0.091	N	N	N	N	Y	1	2	4	7
0.060	0.105	N	N	N	N	N	1	2	4	8
0.074	0.081	N	N	N	N	Y	1	1	3	7
0.060	0.049	N	N	N	Y	Y	1	2	3	7
0.060	0.061	N	N	N	N	Y	1	2	4	7
0.060	0.116	N	N	N	N	N	1	2	4	8

**Table A140:** Set 2, trials 31–40 summary data (average actual PR values).

Average Error	Avg Err	Lowest	Correct				False			
			At 0.01	At 0.025	At 0.05	At 0.1	At 0.01	At 0.025	At 0.05	At 0.10
0.053	0.108	N	N	N	N	N	0	5	6	8
0.109	0.122	N	N	N	N	N	0	0	1	6
0.053	0.071	N	N	N	N	Y	1	5	6	7
0.091	0.111	N	N	N	N	N	0	0	1	5
0.168	0.110	N	N	N	N	N	0	0	0	1
0.048	0.146	N	N	N	N	N	1	5	7	9
0.038	0.013	N	N	Y	Y	Y	2	4	6	8
0.261	0.244	N	N	N	N	N	0	0	0	0
0.140	0.112	N	N	N	N	N	0	0	0	2
0.060	0.116	N	N	N	N	N	1	2	4	8

#### Appendix B.2.5. Trials 41–50

Data from trials 41 through 50, for the second GDES network, are presented in Table A141, Table A142, Table A143, Table A144, Table A145, Table A146, Table A147, Table A148, Table A149, Table A150, Table A151, Table A152, Table A153, Table A154, Table A155 and Table A156. For the 0.5 PR value, a % ERR AVG of 43.07% was observed. At an 0.025 margin of error, there are four correctly identified patients and an average of 1.8 incorrectly identified patients. Expanding this margin to 0.05 yielded nine correctly identified patients with an average of three incorrect patients being identified per trial. Thus, at an error margin of 0.05, the ratio of correct-to-incorrect patients is 3. This is outperformed by the second PR approach, as shown in Table A154. At an error margin of 0.025, its correct-to-incorrect ratio is 3.57. Table A155 and Table A156 show that, ultimately, the highest ratios found for the other approaches were 2.31 at an error margin of 0.025 and 2.65 at 0.05, respectively.

**Table A141:** Set 2, trials 41–50 data (single 0.5 PR value).

Actual	Average	Average Error	Max Error	Min Error
0.589	0.562	0.051	0.066	0.037
0.569	0.582	0.045	0.061	0.033
0.644	0.487	0.163	0.194	0.135
0.496	0.573	0.077	0.099	0.064
0.565	0.591	0.048	0.065	0.035
0.565	0.512	0.072	0.094	0.057
0.516	0.509	0.047	0.070	0.043
0.782	0.512	0.270	0.302	0.241
0.538	0.591	0.064	0.079	0.051
0.550	0.487	0.088	0.118	0.065

**Table A142:** Set 2, trials 41–50 data (actual PR value).

Actual	Average	Average Error	Max Error	Min Error
0.550	0.580	0.050	0.082	0.037
0.550	0.605	0.066	0.102	0.056
0.644	0.487	0.163	0.194	0.135
0.550	0.541	0.044	0.070	0.037
0.499	0.616	0.117	0.152	0.099
0.549	0.518	0.056	0.080	0.051
0.550	0.514	0.061	0.085	0.053
0.782	0.482	0.299	0.326	0.272
0.526	0.616	0.090	0.128	0.083
0.550	0.487	0.088	0.118	0.065

**Table A143:** Set 2, trials 41–50 data (all 0.5 PR values).

Actual	Average	Average Error	Max Error	Min Error
0.550	0.562	0.038	0.053	0.027
0.550	0.582	0.048	0.063	0.035
0.550	0.487	0.088	0.118	0.065
0.550	0.530	0.047	0.065	0.032
0.550	0.591	0.056	0.072	0.041
0.500	0.482	0.060	0.086	0.051
0.550	0.509	0.064	0.087	0.052
0.550	0.487	0.088	0.118	0.065
0.550	0.591	0.056	0.072	0.041
0.550	0.487	0.088	0.118	0.065

**Table A144:** Set 2, trials 41–50 data (average actual PR values).

Actual	Average	Average Error	Max Error	Min Error
0.549	0.562	0.038	0.054	0.027
0.549	0.582	0.049	0.063	0.036
0.637	0.487	0.157	0.188	0.129
0.547	0.571	0.047	0.067	0.042
0.500	0.591	0.094	0.109	0.087
0.550	0.512	0.060	0.082	0.050
0.509	0.509	0.047	0.068	0.042
0.803	0.487	0.316	0.347	0.288
0.528	0.591	0.071	0.086	0.060
0.550	0.487	0.088	0.118	0.065

**Table A145:** Set 2, trials 41–50 data (single 0.5 PR value).

Test Avg	Avg Err	Min	Max
0.575	0.014	0.011	0.018
0.594	0.025	0.013	0.032
0.673	0.029	0.004	0.049
0.506	0.010	0.001	0.024
0.534	0.030	0.017	0.044
0.540	0.025	0.039	0.074
0.543	0.027	0.014	0.044
0.498	0.283	0.275	0.301
0.565	0.026	0.027	0.027
0.536	0.014	0.000	0.099

**Table A146:** Set 2, trials 41–50 data (actual PR value).

Test Avg	Avg Err	Min	Max
0.545	0.005	0.000	0.011
0.600	0.049	0.031	0.107
0.673	0.029	0.004	0.049
0.566	0.016	0.008	0.026
0.526	0.027	0.052	0.073
0.531	0.018	0.046	0.068
0.548	0.002	0.015	0.017
0.498	0.283	0.275	0.301
0.559	0.033	0.018	0.039
0.536	0.014	0.000	0.099

**Table A147:** Set 2, trials 41–50 data (all 0.5 PR values).

Test Avg	Avg Err	Min	Max
0.575	0.025	0.020	0.028
0.594	0.044	0.033	0.051
0.673	0.123	0.098	0.143
0.480	0.070	0.060	0.077
0.534	0.016	0.003	0.030
0.479	0.021	0.067	0.086
0.543	0.007	0.010	0.020
0.498	0.052	0.043	0.069
0.565	0.014	0.015	0.015
0.536	0.014	0.000	0.099

**Table A148:** Set 2, trials 41–50 data (average actual PR values).

Test Avg	Avg Err	Min	Max
0.575	0.026	0.022	0.029
0.594	0.045	0.034	0.052
0.673	0.037	0.012	0.056
0.506	0.041	0.027	0.050
0.534	0.035	0.021	0.048
0.540	0.010	0.055	0.059
0.543	0.034	0.022	0.052
0.498	0.305	0.296	0.322
0.565	0.036	0.037	0.037
0.536	0.014	0.000	0.099

**Table A149:** Set 2, trials 41–50 data (single 0.5 PR value).

% Err Avg	% Max Avg	% Min Avg
27.30%	28.71%	27.41%
55.21%	94.38%	21.98%
17.96%	36.01%	2.07%
13.42%	37.52%	0.87%
62.55%	67.74%	50.36%
34.99%	78.68%	69.65%
56.47%	102.73%	19.78%
105.16%	113.71%	99.59%
41.69%	51.61%	33.52%
16.01%	83.19%	0.00%
43.07%	69.43%	32.52%

**Table A150:** Set 2, trials 41–50 data (actual PR value).

% Err Avg	% Max Avg	% Min Avg
10.41%	13.53%	0.29%
75.29%	190.81%	30.38%
17.96%	36.01%	2.07%
34.88%	69.08%	12.10%
23.29%	73.12%	34.19%
32.50%	91.73%	85.41%
2.99%	29.09%	19.47%
94.78%	100.94%	92.22%
36.60%	47.27%	14.01%
16.01%	83.19%	0.00%
34.47%	73.48%	29.01%

**Table A151:** Set 2, trials 41–50 data (all 0.5 PR values).

% Err Avg	% Max Avg	% Min Avg
64.87%	103.48%	38.40%
91.43%	144.92%	51.67%
140.26%	220.25%	82.77%
148.90%	186.00%	118.98%
27.75%	41.06%	7.40%
34.77%	132.02%	99.25%
11.36%	23.01%	19.72%
59.08%	66.46%	58.28%
25.73%	35.76%	20.18%
16.01%	83.19%	0.00%
62.02%	103.62%	49.67%

**Table A152:** Set 2, trials 41–50 data (average actual PR values).

% Err Avg	% Max Avg	% Min Avg
67.44%	107.65%	40.35%
92.44%	145.30%	52.95%
23.42%	43.51%	6.13%
86.39%	74.60%	63.97%
37.23%	54.47%	19.27%
16.69%	110.22%	71.78%
73.21%	124.07%	31.51%
96.42%	102.62%	92.70%
51.53%	60.63%	42.34%
16.01%	83.19%	0.00%
56.08%	90.63%	42.10%

**Table A153:** Set 2, trials 41–50 summary data (single 0.5 PR value).

Average Error	Avg Err	Lowest	Correct				False			
			At 0.01	At 0.025	At 0.05	At 0.1	At 0.01	At 0.025	At 0.05	At 0.10
0.051	0.014	Y	N	Y	Y	Y	0	1	4	9
0.045	0.025	N	N	Y	Y	Y	2	4	5	8
0.163	0.029	Y	N	N	Y	Y	0	0	0	0
0.077	0.010	N	N	Y	Y	Y	1	1	2	7
0.048	0.030	N	N	N	Y	Y	3	4	5	8
0.072	0.025	N	N	N	Y	Y	0	1	3	7
0.047	0.027	N	N	N	Y	Y	2	4	6	8
0.270	0.283	N	N	N	N	N	0	0	0	0
0.064	0.026	N	N	N	Y	Y	1	2	4	7
0.088	0.014	N	N	Y	Y	Y	0	1	1	5

**Table A154:** Set 2, trials 41–50 summary data (actual PR value).

Average Error	Avg Err	Lowest	Correct				False			
			At 0.01	At 0.025	At 0.05	At 0.1	At 0.01	At 0.025	At 0.05	At 0.10
0.050	0.005	Y	Y	Y	Y	Y	0	2	6	7
0.066	0.049	N	N	N	Y	Y	1	2	6	6
0.163	0.029	Y	N	N	Y	Y	0	0	0	0
0.044	0.016	N	N	Y	Y	Y	2	4	5	8
0.117	0.027	Y	N	N	Y	Y	0	0	1	4
0.056	0.018	N	N	Y	Y	Y	2	2	5	7
0.061	0.002	Y	Y	Y	Y	Y	1	1	3	7
0.299	0.283	N	N	N	N	N	0	0	0	0
0.090	0.033	N	N	N	Y	Y	2	2	2	6
0.088	0.014	N	N	Y	Y	Y	0	1	1	5

**Table A155:** Set 2, trials 41–50 summary data (all 0.5 PR values).

Average Error	Avg Err	Lowest	Correct				False			
			At 0.01	At 0.025	At 0.05	At 0.1	At 0.01	At 0.025	At 0.05	At 0.10
0.038	0.025	N	N	Y	Y	Y	2	4	7	8
0.048	0.044	N	N	N	Y	Y	2	5	5	8
0.088	0.123	N	N	N	N	N	0	2	2	6
0.047	0.070	N	N	N	N	Y	1	2	5	8
0.056	0.016	N	N	Y	Y	Y	0	3	4	7
0.060	0.021	N	N	Y	Y	Y	2	2	5	7
0.064	0.007	Y	Y	Y	Y	Y	0	2	3	7
0.088	0.052	N	N	N	N	Y	0	2	2	5
0.056	0.014	N	N	Y	Y	Y	0	3	4	7
0.088	0.014	N	N	Y	Y	Y	0	1	1	5

**Table A156:** Set 2, trials 41–50 summary data (average actual PR values).

Average Error	Avg Err	Lowest	Correct				False			
			At 0.01	At 0.025	At 0.05	At 0.1	At 0.01	At 0.025	At 0.05	At 0.10
0.038	0.026	N	N	N	Y	Y	2	4	7	8
0.049	0.045	N	N	N	Y	Y	2	5	5	8
0.157	0.037	Y	N	N	Y	Y	0	0	0	1
0.047	0.041	N	N	N	Y	Y	3	5	6	7
0.094	0.035	N	N	N	Y	Y	0	1	1	5
0.060	0.010	N	N	Y	Y	Y	1	1	3	7
0.047	0.034	N	N	N	Y	Y	1	4	6	8
0.316	0.305	N	N	N	N	N	0	0	0	0
0.071	0.036	N	N	N	Y	Y	1	1	5	7
0.088	0.014	N	N	Y	Y	Y	0	1	1	5

The third network design, which is depicted in Figure 5, combines the heart rate and end-tidal carbon dioxide facts with a single rule, while respiratory rate and blood pressure are combined using a second. These two rules produce facts that serve as inputs to the final rule, leading to the output fact. Notably, this network design tended to produce greater accuracy than the previous two networks.

### Appendix B.3

#### Appendix B.3.1. Trials 1–10

Table A157, Table A158, Table A159, Table A160, Table A161, Table A162, Table A163, Table A164, Table A165, Table A166, Table A167, Table A168, Table A169, Table A170 and Table A171 display the results form trials 1 through 10 with the third GDES network. A % ERR AVG of 33.49% was produced with the single 0.5 PR value. This value is lower than those found in any other set. At an error margin of 0.025, seven patients were correctly identified with an average of 1.7 patients also being incorrectly identified per trial. Expanding the error margin to 0.05 correctly identifies one additional patient (eight total) and increases the average number of incorrectly identified patients per trial from 1.7 to 3.9. As shown in Table A169, at an error margin of 0.025, five correct patients fall within the margin while an average of 1.1 incorrect patients are also identified. This leads to a correct-to-incorrect ratio of 4.54. This is the highest among all of these trials. The highest ratios and their error margins for the other techniques, presented in Table 5, Table A170 and Table A171, are 4.12 at 0.025, 1.18 at 0.025, and 3.68 at 0.025, respectively.

**Table A157:** Set 3, trials 1–10 data (single 0.5 PR value).

Actual	Average	Average Error	Max Error	Min Error
0.398	0.453	0.056	0.071	0.041
0.435	0.452	0.026	0.032	0.017
0.720	0.606	0.133	0.156	0.110
0.516	0.606	0.090	0.112	0.081
0.472	0.453	0.028	0.043	0.029
0.685	0.603	0.112	0.138	0.086
0.664	0.603	0.100	0.125	0.074
0.501	0.584	0.085	0.106	0.076
0.530	0.603	0.082	0.105	0.074
0.592	0.603	0.069	0.094	0.052

**Table A158:** Set 3, trials 1–10 data (actual PR value).

Actual	Average	Average Error	Max Error	Min Error
0.370	0.487	0.117	0.153	0.088
0.439	0.487	0.061	0.097	0.044
0.720	0.603	0.136	0.161	0.110
0.516	0.603	0.092	0.116	0.083
0.431	0.487	0.067	0.103	0.045
0.685	0.603	0.112	0.138	0.086
0.664	0.603	0.100	0.125	0.074
0.499	0.588	0.091	0.112	0.083
0.530	0.603	0.082	0.105	0.074
0.592	0.603	0.069	0.094	0.052

**Table A159:** Set 3, trials 1–10 data (all 0.5 PR values).

Actual	Average	Average Error	Max Error	Min Error
0.500	0.453	0.047	0.063	0.046
0.500	0.453	0.047	0.063	0.046
0.500	0.603	0.104	0.128	0.094
0.500	0.603	0.104	0.128	0.094
0.500	0.453	0.047	0.063	0.046
0.500	0.603	0.104	0.128	0.094
0.500	0.603	0.104	0.128	0.094
0.500	0.584	0.086	0.107	0.077
0.500	0.603	0.104	0.128	0.094
0.500	0.603	0.104	0.128	0.094

**Table A160:** Set 3, trials 1–10 data (average actual PR values).

Actual	Average	Average Error	Max Error	Min Error
0.371	0.453	0.082	0.096	0.066
0.437	0.453	0.026	0.041	0.016
0.712	0.603	0.130	0.155	0.105
0.511	0.603	0.096	0.119	0.086
0.432	0.453	0.029	0.044	0.017
0.685	0.603	0.112	0.138	0.086
0.642	0.603	0.087	0.112	0.065
0.517	0.584	0.074	0.094	0.066
0.544	0.603	0.074	0.098	0.064
0.533	0.603	0.080	0.103	0.072

**Table A161:** Set 3, trials 1–10 data (single 0.5 PR value).

Test Avg	Avg Err	Min	Max
0.393	0.005	0.000	0.019
0.446	0.011	0.000	0.037
0.816	0.096	0.081	0.102
0.523	0.007	0.005	0.026
0.472	0.000	0.000	0.000
0.707	0.022	0.009	0.037
0.641	0.023	0.001	0.048
0.550	0.048	0.024	0.069
0.554	0.025	0.016	0.031
0.540	0.052	0.024	0.135

**Table A162:** Set 3, trials 1–10 data (actual PR value).

Test Avg	Avg Err	Min	Max
0.376	0.007	0.011	0.016
0.473	0.034	0.004	0.121
0.816	0.096	0.081	0.102
0.523	0.007	0.005	0.026
0.488	0.057	0.008	0.095
0.707	0.022	0.009	0.037
0.641	0.023	0.001	0.048
0.547	0.048	0.023	0.070
0.554	0.025	0.016	0.031
0.540	0.052	0.024	0.135

**Table A163:** Set 3, trials 1–10 data (all 0.5 PR values).

Test Avg	Avg Err	Min	Max
0.393	0.107	0.102	0.121
0.446	0.054	0.028	0.065
0.816	0.316	0.300	0.322
0.523	0.023	0.010	0.042
0.472	0.028	0.028	0.028
0.707	0.207	0.194	0.222
0.641	0.141	0.117	0.164
0.550	0.050	0.025	0.071
0.554	0.054	0.045	0.060
0.540	0.040	0.044	0.115

**Table A164:** Set 3, trials 1–10 data (average actual PR values).

Test Avg	Avg Err	Min	Max
0.393	0.022	0.009	0.027
0.446	0.009	0.002	0.035
0.816	0.104	0.088	0.110
0.523	0.012	0.001	0.031
0.472	0.040	0.040	0.040
0.707	0.022	0.009	0.037
0.641	0.000	0.022	0.025
0.550	0.033	0.008	0.054
0.554	0.010	0.001	0.016
0.540	0.006	0.077	0.082

**Table A165:** Set 3, trials 1–10 data (single 0.5 PR value).

% Err Avg	% Max Avg	% Min Avg
9.43%	25.95%	0.00%
40.78%	222.22%	0.00%
72.33%	92.43%	51.70%
8.23%	32.28%	4.90%
0.00%	0.00%	0.00%
19.69%	42.95%	6.90%
22.60%	37.88%	0.68%
57.03%	91.52%	22.33%
29.85%	41.27%	14.74%
74.94%	143.69%	45.19%
33.49%	73.02%	14.64%

**Table A166:** Set 3, trials 1–10 data (actual PR value).

% Err Avg	% Max Avg	% Min Avg
5.72%	18.29%	6.86%
55.62%	275.94%	4.11%
70.67%	92.81%	49.98%
8.08%	31.31%	4.76%
85.89%	209.77%	7.75%
19.69%	42.95%	6.90%
22.60%	37.88%	0.68%
52.99%	84.18%	20.22%
29.85%	41.27%	14.74%
74.94%	143.69%	45.19%
42.61%	97.81%	16.12%

**Table A167:** Set 3, trials 1–10 data (all 0.5 PR values).

% Err Avg	% Max Avg	% Min Avg
228.34%	223.93%	190.82%
115.84%	102.93%	61.47%
302.48%	341.66%	233.92%
21.97%	44.10%	7.80%
59.59%	61.47%	44.34%
198.03%	235.39%	151.27%
135.46%	173.75%	90.84%
57.93%	92.22%	23.42%
51.75%	63.76%	35.09%
37.85%	122.21%	33.92%
120.92%	146.14%	87.29%

**Table A168:** Set 3, trials 1–10 data (average actual PR values).

% Err Avg	% Max Avg	% Min Avg
26.48%	41.00%	8.81%
32.88%	218.75%	4.84%
79.69%	103.89%	56.76%
12.48%	35.57%	0.84%
138.55%	240.96%	90.70%
19.69%	42.95%	6.90%
0.16%	33.98%	22.34%
44.89%	81.74%	8.76%
13.47%	25.02%	1.02%
8.13%	114.37%	74.24%
37.64%	93.82%	27.52%

**Table A169:** Set 3, trials 1–10 summary data (actual PR value).

Average Error	Avg Err	Lowest	Correct				False			
			At 0.01	At 0.025	At 0.05	At 0.1	At 0.01	At 0.025	At 0.05	At 0.10
0.117	0.007	Y	Y	Y	Y	Y	0	0	0	3
0.061	0.034	N	N	N	Y	Y	1	2	5	8
0.136	0.096	N	N	N	N	Y	0	1	1	2
0.092	0.007	Y	Y	Y	Y	Y	0	2	4	6
0.067	0.057	N	N	N	N	Y	1	1	4	7
0.112	0.022	Y	N	Y	Y	Y	0	0	1	3
0.100	0.023	Y	N	Y	Y	Y	0	0	1	3
0.091	0.048	N	N	N	Y	Y	1	1	4	6
0.082	0.025	N	N	Y	Y	Y	1	2	4	6
0.069	0.052	N	N	N	N	Y	2	2	5	7

**Table A170:** Set 3, trials 1–10 summary data (all 0.5 PR values).

Average Error	Avg Err	Lowest	Correct				False			
			At 0.01	At 0.025	At 0.05	At 0.1	At 0.01	At 0.025	At 0.05	At 0.10
0.047	0.107	N	N	N	N	N	2	3	5	9
0.047	0.054	N	N	N	N	Y	2	3	5	8
0.104	0.316	N	N	N	N	N	1	2	3	6
0.104	0.023	N	N	Y	Y	Y	1	1	2	5
0.047	0.028	N	N	N	Y	Y	2	3	4	8
0.104	0.207	N	N	N	N	N	1	2	3	6
0.104	0.141	N	N	N	N	N	1	2	3	6
0.086	0.050	N	N	N	Y	Y	0	2	4	6
0.104	0.054	N	N	N	N	Y	1	2	3	5
0.104	0.040	N	N	N	Y	Y	1	2	2	5

**Table A171:** Set 3, trials 1–10 summary data (average actual PR values).

Average Error	Avg Err	Lowest	Correct				False			
			At 0.01	At 0.025	At 0.05	At 0.1	At 0.01	At 0.025	At 0.05	At 0.10
0.082	0.022	Y	N	Y	Y	Y	0	0	0	5
0.026	0.009	N	Y	Y	Y	Y	3	4	7	9
0.130	0.104	N	N	N	N	N	1	1	1	2
0.096	0.012	Y	N	Y	Y	Y	0	1	4	6
0.029	0.040	N	N	N	Y	Y	3	5	7	9
0.112	0.022	Y	N	Y	Y	Y	0	0	1	3
0.087	0.000	Y	Y	Y	Y	Y	0	0	2	5
0.074	0.033	N	N	N	Y	Y	1	3	4	7
0.074	0.010	N	N	Y	Y	Y	1	3	3	7
0.080	0.006	Y	Y	Y	Y	Y	0	2	4	6

#### Appendix B.3.2. Trials 11–20

Table A172, Table A173, Table A174, Table A175, Table A176, Table A177, Table A178, Table A179, Table A180, Table A181, Table A182, Table A183, Table A184 and Table A185 present the results from trials 11 through 20, with the third GDES network. The % err avg from the base 0.5 PR values is 87.11%. At an 0.05 margin of error, five patients fall within the margin with an average of 5.6 incorrect patients per trial also being identified. Expanding to a 0.1 margin of error allows for seven correct patient identifications with an average of 7.1 incorrect per trial. It is worth noting that, at an error margin of 0.01, three target patients fall within the margin with an average of 0.6 incorrect patients per trial also falling within this margin. While this is a relatively low number of correctly identified patients, this is a high correct-to-incorrect ratio of 5. Furthermore, as seen in Table 6, at an error margin of 0.01, four correct patients are identified with an average of 0.5 incorrect patients falling within the margin of error as well. The correct-to-incorrect ratio in this case is thus 8.00. While it is a relatively low number of correct patients identified, given the low error margin, this is noteworthy, as the ratio is the highest from any batches of trials. Table 7 shows that, in that case, no patients, correct or incorrect, were identified within a 0.1 margin of error. As seen in Table A185, the only margin of error that included any correct patients was 0.1 with one correct patient falling within the margin and an average of 1.7 incorrect patients also being included, resulting in a ratio of 0.59.

**Table A172:** Set 3, trials 11–20 data (single 0.5 PR value).

Actual	Average	Average Error	Max Error	Min Error
0.970	0.916	0.056	0.092	0.027
0.924	0.916	0.054	0.089	0.035
0.916	0.916	0.058	0.093	0.039
0.924	0.944	0.030	0.057	0.025
0.984	0.916	0.068	0.106	0.029
0.925	0.916	0.054	0.089	0.035
1.000	0.916	0.084	0.122	0.040
0.695	0.916	0.227	0.266	0.208
0.940	0.916	0.049	0.084	0.030
0.862	0.916	0.093	0.132	0.059

**Table A173:** Set 3, trials 11–20 data (actual PR value).

Actual	Average	Average Error	Max Error	Min Error
0.970	0.916	0.056	0.092	0.027
0.924	0.916	0.054	0.089	0.035
0.916	0.916	0.058	0.093	0.039
0.516	0.603	0.092	0.116	0.083
0.984	0.916	0.068	0.106	0.029
0.925	0.916	0.054	0.089	0.035
0.925	0.916	0.054	0.089	0.035
1.000	0.916	0.084	0.122	0.040
0.940	0.916	0.049	0.084	0.030
0.862	0.916	0.093	0.132	0.059

**Table A174:** Set 3, trials 11–20 data (all 0.5 PR values).

Actual	Average	Average Error	Max Error	Min Error
0.500	0.896	0.396	0.456	0.342
0.500	0.916	0.416	0.460	0.378
0.500	0.916	0.416	0.460	0.378
0.500	0.916	0.416	0.460	0.378
0.500	0.916	0.416	0.460	0.378
0.500	0.916	0.416	0.460	0.378
0.500	0.916	0.416	0.460	0.378
0.500	0.916	0.416	0.460	0.378
0.500	0.916	0.416	0.460	0.378
0.500	0.916	0.416	0.460	0.378

**Table A175:** Set 3, trials 11–20 data (average actual PR values).

Actual	Average	Average Error	Max Error	Min Error
0.669	0.916	0.248	0.291	0.229
0.757	0.916	0.177	0.216	0.146
0.883	0.916	0.077	0.115	0.050
0.722	0.916	0.206	0.244	0.181
0.744	0.916	0.188	0.226	0.159
0.685	0.916	0.235	0.276	0.216
0.826	0.916	0.123	0.161	0.081
0.566	0.916	0.350	0.395	0.313
0.765	0.916	0.171	0.209	0.138
0.804	0.916	0.140	0.178	0.099

**Table A176:** Set 3, trials 11–20 data (single 0.5 PR value).

Test Avg	Avg Err	Min	Max
0.972	0.002	0.009	0.011
0.915	0.009	0.039	0.059
0.977	0.062	0.040	0.069
0.964	0.040	0.017	0.076
0.880	0.104	0.006	0.167
0.952	0.027	0.017	0.041
0.937	0.063	0.034	0.101
0.953	0.258	0.209	0.299
0.943	0.004	0.012	0.014
0.664	0.198	0.045	0.290

**Table A177:** Set 3, trials 11–20 data (actual PR value).

Test Avg	Avg Err	Min	Max
0.972	0.002	0.009	0.011
0.915	0.009	0.039	0.059
0.977	0.062	0.040	0.069
0.523	0.007	0.005	0.026
0.880	0.104	0.006	0.167
0.952	0.027	0.017	0.041
0.937	0.012	0.026	0.042
0.953	0.047	0.007	0.097
0.943	0.004	0.012	0.014
0.664	0.198	0.045	0.290

**Table A178:** Set 3, trials 11–20 data (all 0.5 PR values).

Test Avg	Avg Err	Min	Max
0.857	0.357	0.276	0.436
0.915	0.415	0.365	0.463
0.977	0.477	0.456	0.484
0.964	0.464	0.441	0.500
0.880	0.380	0.318	0.478
0.952	0.452	0.442	0.466
0.937	0.437	0.399	0.467
0.953	0.453	0.404	0.493
0.943	0.443	0.428	0.454
0.664	0.164	0.072	0.317

**Table A179:** Set 3, trials 11–20 data (average actual PR values).

Test Avg	Avg Err	Min	Max
0.972	0.303	0.292	0.312
0.915	0.158	0.108	0.206
0.977	0.095	0.073	0.102
0.964	0.243	0.220	0.279
0.880	0.136	0.074	0.234
0.952	0.268	0.258	0.281
0.937	0.112	0.074	0.141
0.953	0.387	0.338	0.428
0.943	0.178	0.163	0.189
0.664	0.140	0.014	0.232

**Table A180:** Set 3, trials 11–20 data (single 0.5 PR value).

% Err Avg	% Max Avg	% Min Avg
3.84%	41.51%	9.28%
17.07%	110.48%	66.11%
106.67%	177.00%	43.17%
132.34%	304.00%	29.82%
151.92%	157.75%	20.76%
50.67%	116.05%	19.13%
74.55%	83.96%	83.09%
113.53%	143.20%	78.69%
7.93%	46.59%	13.63%
212.52%	220.36%	76.47%
87.11%	140.09%	44.02%

**Table A181:** Set 3, trials 11–20 data (actual PR value).

% Err Avg	% Max Avg	% Min Avg
3.84%	41.51%	9.28%
17.07%	110.48%	66.11%
106.67%	177.00%	43.17%
8.08%	31.31%	4.76%
151.92%	157.75%	20.76%
50.67%	116.05%	19.13%
22.49%	118.91%	29.26%
56.17%	79.39%	17.54%
7.93%	46.59%	13.63%
212.52%	220.36%	76.47%
63.74%	109.94%	30.01%

**Table A182:** Set 3, trials 11–20 data (all 0.5 PR values).

% Err Avg	% Max Avg	% Min Avg
90.21%	127.43%	60.47%
99.77%	122.34%	79.33%
114.78%	127.89%	99.00%
111.64%	132.12%	95.85%
91.47%	126.30%	69.01%
108.82%	123.00%	96.07%
105.16%	123.27%	86.72%
108.89%	130.27%	87.70%
106.66%	119.83%	93.02%
39.35%	83.76%	15.65%
97.68%	121.62%	78.28%

**Table A183:** Set 3, trials 11–20 data (average actual PR values).

% Err Avg	% Max Avg	% Min Avg
122.15%	136.12%	100.31%
88.92%	141.14%	50.09%
122.23%	202.80%	63.37%
117.88%	153.49%	89.96%
72.54%	147.22%	32.52%
113.80%	129.82%	93.43%
91.08%	174.61%	45.71%
110.55%	136.60%	85.66%
104.32%	136.64%	77.92%
99.83%	129.76%	13.57%
104.33%	148.82%	65.25%

**Table A184:** Set 3, trials 11–20 summary data (single 0.5 PR value).

Average Error	Avg Err	Lowest	Correct				False			
			At 0.01	At 0.025	At 0.05	At 0.1	At 0.01	At 0.025	At 0.05	At 0.10
0.056	0.002	Y	Y	Y	Y	Y	2	4	6	8
0.054	0.009	Y	Y	Y	Y	Y	0	2	7	8
0.058	0.062	N	N	N	N	Y	1	2	7	8
0.030	0.040	N	N	N	Y	Y	1	4	8	9
0.068	0.104	N	N	N	N	N	1	3	7	8
0.054	0.027	N	N	N	Y	Y	0	3	7	8
0.084	0.063	N	N	N	N	Y	0	1	5	7
0.227	0.258	N	N	N	N	N	0	0	1	1
0.049	0.004	N	Y	Y	Y	Y	1	5	7	8
0.093	0.198	N	N	N	N	N	0	1	1	6

**Table A185:** Set 3, trials 11–20 summary data (average actual PR values).

Average Error	Avg Err	Lowest	Correct				False			
			At 0.01	At 0.025	At 0.05	At 0.1	At 0.01	At 0.025	At 0.05	At 0.10
0.248	0.303	N	N	N	N	N	1	1	1	1
0.177	0.158	N	N	N	N	N	0	0	0	1
0.077	0.095	N	N	N	N	Y	1	1	2	8
0.206	0.243	N	N	N	N	N	0	0	0	1
0.188	0.136	N	N	N	N	N	0	0	0	1
0.235	0.268	N	N	N	N	N	0	1	1	1
0.123	0.112	N	N	N	N	N	0	0	0	2
0.350	0.387	N	N	N	N	N	0	0	0	1
0.171	0.178	N	N	N	N	N	0	0	0	0
0.140	0.140	N	N	N	N	N	0	0	0	1

#### Appendix B.3.3. Trials 21–30

Data from trials 21 through 30 with the third GDES network are presented in Table A186, Table A187, Table A188, Table A189, Table A190, Table A191, Table A192, Table A193, Table A194, Table A195, Table A196, Table A197, Table A198, Table A199 and Table A200. A % err avg of 40.95% was produced by the base 0.5 PR value approach. Seven target patients fell within the 0.025 margin of error, along with an average of 1.9 incorrect patients per trial. Expanding the margin to 0.05 yielded nine correctly identified patients with an average of four incorrect patients also being identified per trial. As shown in Table 8, at an error margin of 0.025, five correct patients are identified while an average of 0.9 incorrect patients also fall within the margin. The correct-to-incorrect ratio, in this case, is 5.56. This is the highest ratio from any margin of error displayed in Table 8. The techniques presented in Table A198, Table A199 and Table A200 produce best ratios of 3.68 at 0.025, 0.91 at 0.025, and 2 at 0.025, respectively.

**Table A186:** Set 3, trials 21–30 data (single 0.5 PR value).

Actual	Average	Average Error	Max Error	Min Error
0.448	0.507	0.060	0.076	0.041
0.485	0.512	0.035	0.060	0.017
0.541	0.507	0.042	0.054	0.044
0.484	0.545	0.061	0.080	0.044
0.522	0.507	0.032	0.047	0.030
0.685	0.604	0.111	0.138	0.085
0.448	0.507	0.060	0.076	0.041
0.634	0.555	0.084	0.101	0.070
0.504	0.507	0.029	0.043	0.026
0.600	0.507	0.093	0.113	0.081

**Table A187:** Set 3, trials 21–30 data (actual PR value).

Actual	Average	Average Error	Max Error	Min Error
0.370	0.502	0.132	0.170	0.091
0.439	0.502	0.075	0.114	0.045
0.560	0.502	0.082	0.123	0.053
0.600	0.538	0.071	0.101	0.049
0.431	0.502	0.082	0.120	0.048
0.685	0.604	0.111	0.138	0.085
0.583	0.502	0.097	0.141	0.063
0.599	0.553	0.061	0.085	0.047
0.466	0.502	0.054	0.093	0.053
0.600	0.502	0.111	0.155	0.073

**Table A188:** Set 3, trials 21–30 data (all 0.5 PR values).

Actual	Average	Average Error	Max Error	Min Error
0.600	0.507	0.093	0.113	0.081
0.600	0.507	0.093	0.113	0.081
0.600	0.507	0.093	0.113	0.081
0.600	0.541	0.062	0.078	0.054
0.600	0.507	0.093	0.113	0.081
0.600	0.604	0.069	0.095	0.053
0.600	0.507	0.093	0.113	0.081
0.600	0.555	0.060	0.077	0.047
0.600	0.507	0.093	0.113	0.081
0.600	0.507	0.093	0.113	0.081

**Table A189:** Set 3, trials 21–30 data (average actual PR values).

Actual	Average	Average Error	Max Error	Min Error
0.371	0.507	0.136	0.151	0.116
0.437	0.507	0.070	0.085	0.051
0.552	0.507	0.051	0.065	0.052
0.599	0.541	0.060	0.077	0.054
0.432	0.453	0.029	0.044	0.017
0.685	0.604	0.111	0.138	0.085
0.471	0.507	0.042	0.058	0.024
0.606	0.555	0.064	0.081	0.051
0.469	0.507	0.044	0.060	0.025
0.338	0.507	0.169	0.184	0.149

**Table A190:** Set 3, trials 21–30 data (single 0.5 PR value).

Test Avg	Avg Err	Min	Max
0.443	0.005	0.000	0.019
0.496	0.011	0.000	0.037
0.506	0.034	0.019	0.056
0.498	0.015	0.010	0.021
0.522	0.000	0.000	0.000
0.707	0.022	0.009	0.037
0.485	0.037	0.037	0.037
0.549	0.085	0.073	0.099
0.485	0.019	0.019	0.019
0.584	0.016	0.000	0.115

**Table A191:** Set 3, trials 21–30 data (actual PR value).

Test Avg	Avg Err	Min	Max
0.376	0.007	0.011	0.016
0.480	0.041	0.009	0.121
0.531	0.029	0.019	0.047
0.615	0.015	0.010	0.021
0.488	0.057	0.008	0.095
0.707	0.022	0.009	0.037
0.489	0.094	0.025	0.131
0.513	0.086	0.071	0.103
0.472	0.006	0.019	0.027
0.577	0.023	0.000	0.161

**Table A192:** Set 3, trials 21–30 data (all 0.5 PR values).

Test Avg	Avg Err	Min	Max
0.443	0.157	0.152	0.171
0.496	0.104	0.078	0.115
0.506	0.094	0.078	0.115
0.498	0.102	0.095	0.106
0.522	0.078	0.078	0.078
0.707	0.107	0.094	0.122
0.485	0.115	0.115	0.115
0.549	0.051	0.039	0.065
0.485	0.115	0.115	0.115
0.584	0.016	0.000	0.115

**Table A193:** Set 3, trials 21–30 data (average actual PR values).

Test Avg	Avg Err	Min	Max
0.443	0.072	0.059	0.077
0.496	0.059	0.048	0.085
0.506	0.046	0.030	0.067
0.498	0.100	0.094	0.105
0.472	0.040	0.040	0.040
0.707	0.022	0.009	0.037
0.485	0.015	0.015	0.015
0.549	0.057	0.045	0.071
0.485	0.016	0.017	0.017
0.584	0.246	0.147	0.262

**Table A194:** Set 3, trials 21–30 data (single 0.5 PR value).

% Err Avg	% Max Avg	% Min Avg
8.76%	24.25%	0.00%
30.20%	222.22%	0.00%
82.17%	102.59%	41.67%
23.79%	47.75%	12.59%
0.00%	0.00%	0.00%
19.94%	43.30%	6.90%
61.30%	90.91%	48.49%
101.06%	104.85%	98.55%
64.59%	71.43%	43.02%
17.72%	101.63%	0.00%
40.95%	80.89%	25.12%

**Table A195:** Set 3, trials 21–30 data (actual PR value).

% Err Avg	% Max Avg	% Min Avg
5.07%	17.62%	6.17%
54.97%	268.00%	7.46%
35.11%	38.30%	35.00%
20.47%	43.25%	9.96%
70.15%	195.45%	6.64%
19.94%	43.30%	6.90%
96.40%	93.11%	39.04%
141.50%	151.07%	121.04%
10.87%	35.55%	28.97%
20.72%	104.07%	0.00%
47.52%	98.97%	26.12%

**Table A196:** Set 3, trials 21–30 data (all 0.5 PR values).

% Err Avg	% Max Avg	% Min Avg
169.67%	188.70%	150.68%
112.65%	101.63%	96.83%
101.25%	101.63%	96.83%
164.91%	175.62%	136.38%
84.14%	96.83%	68.94%
155.25%	227.95%	98.90%
124.06%	142.77%	101.63%
84.60%	83.88%	82.04%
124.06%	142.77%	101.63%
17.72%	101.63%	0.00%
113.83%	136.34%	93.39%

**Table A197:** Set 3, trials 21–30 data (average actual PR values).

% Err Avg	% Max Avg	% Min Avg
52.62%	66.47%	38.63%
83.32%	165.53%	56.17%
89.89%	102.84%	57.97%
165.71%	174.77%	136.56%
138.55%	240.96%	90.70%
19.94%	43.30%	6.90%
34.23%	61.70%	24.87%
89.38%	88.20%	87.68%
37.54%	66.80%	27.55%
145.05%	176.02%	79.70%
85.62%	118.66%	60.67%

**Table A198:** Set 3, trials 21–30 summary data (single 0.5 PR value).

Average Error	Avg Err	Lowest	Correct				False			
			At 0.01	At 0.025	At 0.05	At 0.1	At 0.01	At 0.025	At 0.05	At 0.10
0.060	0.005	Y	Y	Y	Y	Y	0	0	4	8
0.035	0.011	N	N	Y	Y	Y	2	3	7	9
0.042	0.034	N	N	N	Y	Y	1	3	5	9
0.061	0.015	N	N	Y	Y	Y	0	1	3	7
0.032	0.000	Y	Y	Y	Y	Y	1	3	7	9
0.111	0.022	Y	N	Y	Y	Y	0	0	1	3
0.060	0.037	N	N	N	Y	Y	1	1	4	8
0.084	0.085	N	N	N	N	Y	0	2	2	5
0.029	0.019	N	N	Y	Y	Y	2	6	7	9
0.093	0.016	Y	N	Y	Y	Y	0	0	0	4

**Table A199:** Set 3, trials 21–30 summary data (all 0.5 PR values).

Average Error	Avg Err	Lowest	Correct				False			
			At 0.01	At 0.025	At 0.05	At 0.1	At 0.01	At 0.025	At 0.05	At 0.10
0.093	0.157	N	N	N	N	N	0	1	1	5
0.093	0.104	N	N	N	N	N	0	1	1	5
0.093	0.094	N	N	N	N	Y	0	1	1	4
0.062	0.102	N	N	N	N	N	1	2	3	8
0.093	0.078	N	N	N	N	Y	0	1	1	4
0.069	0.107	N	N	N	N	N	2	2	6	7
0.093	0.115	N	N	N	N	N	0	1	1	5
0.060	0.051	N	N	N	N	Y	0	1	3	9
0.093	0.115	N	N	N	N	N	0	1	1	5
0.093	0.016	Y	N	Y	Y	Y	0	0	0	4

**Table A200:** Set 3, trials 21–30 summary data (average actual PR values).

Average Error	Avg Err	Lowest	Correct				False			
			At 0.01	At 0.025	At 0.05	At 0.1	At 0.01	At 0.025	At 0.05	At 0.10
0.136	0.072	Y	N	N	N	Y	0	0	0	0
0.070	0.059	N	N	N	N	Y	1	1	4	7
0.051	0.046	N	N	N	Y	Y	0	2	4	8
0.060	0.100	N	N	N	N	N	1	2	3	8
0.029	0.040	N	N	N	Y	Y	3	5	7	9
0.111	0.022	Y	N	Y	Y	Y	0	0	1	3
0.042	0.015	Y	N	Y	Y	Y	0	2	5	8
0.064	0.057	N	N	N	N	Y	1	1	3	8
0.044	0.016	Y	N	Y	Y	Y	0	2	5	8
0.169	0.246	N	N	N	N	N	0	0	0	0

#### Appendix B.3.4. Trials 31–40

Data from trials 31 through 40 with the third GDES network are presented in Table A201, Table A202, Table A203, Table A204, Table A205, Table A206, Table A207, Table A208, Table A209, Table A210, Table A211, Table A212, Table A213, Table A214, Table A215 and Table A216. A % err avg of 43.71% is produced by the base 0.5 PR technique. As shown in Table A213, at a margin of error of 0.025, six patients are correctly identified by the 0.5 PR technique along with an average of 1.9 incorrect patient identifications per trial. This leads to a correct-to-incorrect ratio of 3.16. Table A216 shows that, at an error margin of 0.01, one correct patient falls within the margin of error while an average of 0.2 incorrect patients fall within the margin, resulting in a ratio of 5.00, the highest of any within these trials. The highest ratios from other techniques, presented in Table A214 and Table A215, are 3.68 at an error margin level of 0.05 and 1.25 at 0.025.

**Table A201:** Set 3, trials 31–40 data (single 0.5 PR value).

Actual	Average	Average Error	Max Error	Min Error
0.498	0.562	0.065	0.081	0.041
0.535	0.562	0.035	0.052	0.015
0.591	0.562	0.046	0.059	0.044
0.517	0.565	0.053	0.087	0.017
0.572	0.562	0.036	0.052	0.030
0.628	0.564	0.074	0.091	0.069
0.498	0.562	0.065	0.081	0.041
0.806	0.564	0.243	0.265	0.226
0.554	0.562	0.033	0.048	0.026
0.700	0.562	0.138	0.163	0.123

**Table A202:** Set 3, trials 31–40 data (actual PR value).

Actual	Average	Average Error	Max Error	Min Error
0.370	0.517	0.147	0.190	0.091
0.439	0.516	0.089	0.134	0.045
0.560	0.517	0.085	0.125	0.061
0.646	0.517	0.136	0.192	0.101
0.431	0.517	0.097	0.140	0.048
0.487	0.517	0.058	0.100	0.062
0.583	0.517	0.098	0.141	0.072
0.700	0.527	0.173	0.224	0.133
0.466	0.517	0.069	0.112	0.054
0.700	0.517	0.183	0.242	0.140

**Table A203:** Set 3, trials 31–40 data (all 0.5 PR values).

Actual	Average	Average Error	Max Error	Min Error
0.700	0.562	0.138	0.163	0.123
0.700	0.562	0.138	0.163	0.123
0.700	0.562	0.138	0.163	0.123
0.700	0.562	0.138	0.163	0.123
0.700	0.562	0.138	0.163	0.123
0.700	0.562	0.138	0.163	0.123
0.700	0.562	0.138	0.163	0.123
0.700	0.567	0.133	0.156	0.116
0.700	0.562	0.138	0.163	0.123
0.700	0.562	0.138	0.163	0.123

**Table A204:** Set 3, trials 31–40 data (average actual PR values).

Actual	Average	Average Error	Max Error	Min Error
0.371	0.562	0.191	0.206	0.166
0.437	0.562	0.125	0.140	0.100
0.552	0.562	0.033	0.048	0.025
0.646	0.562	0.091	0.109	0.083
0.432	0.562	0.130	0.145	0.105
0.490	0.562	0.072	0.088	0.049
0.471	0.562	0.091	0.107	0.066
0.696	0.567	0.130	0.152	0.113
0.469	0.562	0.093	0.109	0.068
0.338	0.562	0.224	0.239	0.199

**Table A205:** Set 3, trials 31–40 data (single 0.5 PR value).

Test Avg	Avg Err	Min	Max
0.493	0.005	0.000	0.019
0.546	0.011	0.000	0.037
0.556	0.034	0.019	0.056
0.535	0.019	0.019	0.019
0.572	0.000	0.000	0.000
0.578	0.051	0.037	0.112
0.535	0.037	0.037	0.037
0.587	0.220	0.217	0.223
0.535	0.019	0.019	0.019
0.676	0.024	0.000	0.165

**Table A206:** Set 3, trials 31–40 data (actual PR value).

Test Avg	Avg Err	Min	Max
0.376	0.007	0.011	0.016
0.487	0.048	0.009	0.121
0.531	0.029	0.019	0.047
0.665	0.019	0.019	0.019
0.488	0.057	0.008	0.095
0.477	0.011	0.095	0.141
0.496	0.086	0.025	0.131
0.478	0.222	0.196	0.231
0.508	0.042	0.027	0.069
0.663	0.037	0.000	0.261

**Table A207:** Set 3, trials 31–40 data (all 0.5 PR values).

Test Avg	Avg Err	Min	Max
0.493	0.207	0.202	0.221
0.546	0.154	0.128	0.165
0.556	0.144	0.128	0.165
0.535	0.165	0.165	0.165
0.572	0.128	0.128	0.128
0.578	0.123	0.035	0.184
0.535	0.165	0.165	0.165
0.587	0.113	0.110	0.116
0.535	0.165	0.165	0.165
0.676	0.024	0.000	0.165

**Table A208:** Set 3, trials 31–40 data (average actual PR values).

Test Avg	Avg Err	Min	Max
0.493	0.122	0.109	0.127
0.546	0.109	0.098	0.135
0.556	0.004	0.017	0.020
0.535	0.111	0.111	0.111
0.572	0.140	0.140	0.140
0.578	0.088	0.027	0.176
0.535	0.065	0.065	0.065
0.587	0.109	0.107	0.113
0.535	0.067	0.067	0.067
0.676	0.338	0.197	0.362

**Table A209:** Set 3, trials 31–40 data (single 0.5 PR value).

% Err Avg	% Max Avg	% Min Avg
8.18%	22.76%	0.00%
30.17%	250.00%	0.00%
74.53%	93.91%	41.67%
35.04%	111.11%	21.28%
0.00%	0.00%	0.00%
68.26%	122.33%	53.90%
57.24%	90.91%	45.51%
90.44%	95.88%	84.04%
56.18%	71.43%	38.54%
17.03%	101.13%	0.00%
43.71%	95.95%	28.49%

**Table A210:** Set 3, trials 31–40 data (actual PR value).

% Err Avg	% Max Avg	% Min Avg
4.55%	17.62%	5.52%
54.18%	268.00%	6.34%
34.22%	37.72%	30.35%
13.59%	18.34%	9.66%
59.29%	195.45%	5.70%
18.10%	227.05%	94.67%
88.09%	93.11%	34.10%
128.37%	146.80%	103.19%
60.37%	127.19%	24.02%
20.36%	107.94%	0.00%
48.11%	123.92%	31.36%

**Table A211:** Set 3, trials 31–40 data (all 0.5 PR values).

% Err Avg	% Max Avg	% Min Avg
149.76%	164.83%	135.15%
111.57%	104.45%	101.13%
103.93%	104.45%	101.13%
119.21%	134.64%	101.13%
92.48%	104.45%	78.46%
88.50%	112.47%	28.56%
119.21%	134.64%	101.13%
84.80%	94.86%	74.60%
119.21%	134.64%	101.13%
17.03%	101.13%	0.00%
100.57%	119.06%	82.24%

**Table A212:** Set 3, trials 31–40 data (average actual PR values).

% Err Avg	% Max Avg	% Min Avg
63.86%	76.58%	52.56%
87.15%	135.20%	69.78%
12.58%	80.00%	35.20%
122.65%	133.49%	101.69%
108.04%	133.52%	96.25%
122.08%	356.71%	30.65%
70.81%	97.21%	60.31%
84.36%	94.59%	73.97%
71.44%	97.29%	61.04%
151.36%	182.05%	82.27%
89.43%	138.66%	66.37%

**Table A213:** Set 3, trials 31–40 summary data (single 0.5 PR value).

Average Error	Avg Err	Lowest	Correct				False			
			At 0.01	At 0.025	At 0.05	At 0.1	At 0.01	At 0.025	At 0.05	At 0.10
0.065	0.005	Y	Y	Y	Y	Y	0	0	4	8
0.035	0.011	N	N	Y	Y	Y	3	4	7	8
0.046	0.034	N	N	N	Y	Y	1	3	4	9
0.053	0.019	Y	N	Y	Y	Y	0	2	4	8
0.036	0.000	Y	Y	Y	Y	Y	1	3	7	8
0.074	0.051	N	N	N	N	Y	0	0	2	8
0.065	0.037	N	N	N	Y	Y	1	1	4	8
0.243	0.220	N	N	N	N	N	0	0	0	0
0.033	0.019	N	N	Y	Y	Y	2	6	7	8
0.138	0.024	Y	N	Y	Y	Y	0	0	0	0

**Table A214:** Set 3, trials 31–40 summary data (actual PR value).

Average Error	Avg Err	Lowest	Correct				False			
			At 0.01	At 0.025	At 0.05	At 0.1	At 0.01	At 0.025	At 0.05	At 0.10
0.147	0.007	Y	Y	Y	Y	Y	0	0	0	1
0.089	0.048	N	N	N	Y	Y	0	0	3	7
0.085	0.029	Y	N	N	Y	Y	0	0	0	6
0.136	0.019	N	N	Y	Y	Y	0	1	1	1
0.097	0.057	N	N	N	N	Y	0	0	2	6
0.058	0.011	N	N	Y	Y	Y	2	5	6	6
0.098	0.086	N	N	N	N	Y	0	0	0	6
0.173	0.222	N	N	N	N	N	0	0	1	2
0.069	0.042	N	N	N	Y	Y	1	3	5	7
0.183	0.037	N	N	N	Y	Y	0	0	1	1

**Table A215:** Set 3, trials 31–40 summary data (all 0.5 PR values).

Average Error	Avg Err	Lowest	Correct				False			
			At 0.01	At 0.025	At 0.05	At 0.1	At 0.01	At 0.025	At 0.05	At 0.10
0.138	0.207	N	N	N	N	N	0	1	1	1
0.138	0.154	N	N	N	N	N	0	1	1	1
0.138	0.144	N	N	N	N	N	0	1	1	1
0.138	0.165	N	N	N	N	N	0	1	1	1
0.138	0.128	N	N	N	N	N	0	1	1	1
0.138	0.123	N	N	N	N	N	0	1	1	1
0.138	0.165	N	N	N	N	N	0	1	1	1
0.133	0.113	N	N	N	N	N	0	0	1	1
0.138	0.165	N	N	N	N	N	0	1	1	1
0.138	0.024	Y	N	Y	Y	Y	0	0	0	0

**Table A216:** Set 3, trials 31–40 summary data (average actual PR values).

Average Error	Avg Err	Lowest	Correct				False			
			At 0.01	At 0.025	At 0.05	At 0.1	At 0.01	At 0.025	At 0.05	At 0.10
0.191	0.122	Y	N	N	N	N	0	0	0	0
0.125	0.109	N	N	N	N	N	0	0	0	4
0.033	0.004	Y	Y	Y	Y	Y	1	5	7	8
0.091	0.111	N	N	N	N	N	0	0	1	5
0.130	0.140	N	N	N	N	N	0	0	0	1
0.072	0.088	N	N	N	N	Y	1	1	4	7
0.091	0.065	N	N	N	N	Y	0	1	1	5
0.130	0.109	N	N	N	N	N	0	0	1	1
0.093	0.067	N	N	N	N	Y	0	1	1	5
0.224	0.338	N	N	N	N	N	0	0	0	0

#### Appendix B.3.5. Trials 41–50

Data from trials 41 through 50 using the third GDES network are presented in Table A217, Table A218, Table A219, Table A220, Table A221, Table A222, Table A223, Table A224, Table A225, Table A226, Table A227, Table A228, Table A229, Table A230 and Table A231. These trials are the most accurate of any presented. The % err avg for the base 0.5 PR technique is 29.96%, the lowest of any batch of trials. An error margin of 0.025 yields nine correctly identified patients, with an average of 1.9 incorrectly identified patients per trial. This is nearly as precise as the results of trials 11 through 20 of set 3. It also identifies three times the number of correct patients. In half of these trials, the target patient was the patient with the lowest margin of error, which is a higher rate than any other batch of trials. Table A229 and Table A230 also have their highest ratios at an error margin of 0.025. Those ratios were 3.89 and 0.95, respectively. Table A231 presents the fourth PR approach and shows that, at an error margin of 0.01, two correct patients were identified with an average of 0.8 incorrect patients falling within the margin.

**Table A217:** Set 3, trials 41–50 data (single 0.5 PR value).

Actual	Average	Average Error	Max Error	Min Error
0.423	0.480	0.058	0.074	0.041
0.460	0.480	0.029	0.044	0.015
0.720	0.603	0.135	0.161	0.110
0.496	0.571	0.075	0.095	0.064
0.497	0.480	0.030	0.044	0.030
0.685	0.603	0.112	0.138	0.086
0.664	0.603	0.099	0.125	0.074
0.563	0.565	0.048	0.067	0.039
0.479	0.480	0.027	0.041	0.026
0.550	0.480	0.070	0.088	0.063

**Table A218:** Set 3, trials 41–50 data (actual PR value).

Actual	Average	Average Error	Max Error	Min Error
0.370	0.494	0.125	0.160	0.091
0.439	0.494	0.068	0.104	0.045
0.720	0.603	0.135	0.161	0.110
0.550	0.575	0.048	0.070	0.039
0.431	0.494	0.074	0.110	0.048
0.685	0.603	0.112	0.138	0.086
0.664	0.603	0.099	0.125	0.074
0.549	0.569	0.047	0.069	0.038
0.466	0.494	0.049	0.086	0.052
0.550	0.494	0.078	0.115	0.047

**Table A219:** Set 3, trials 41–50 data (all 0.5 PR values).

Actual	Average	Average Error	Max Error	Min Error
0.550	0.480	0.070	0.088	0.063
0.550	0.480	0.070	0.088	0.063
0.550	0.603	0.071	0.096	0.062
0.550	0.571	0.047	0.067	0.042
0.550	0.480	0.070	0.088	0.063
0.550	0.603	0.071	0.096	0.062
0.550	0.603	0.071	0.096	0.062
0.550	0.565	0.045	0.064	0.039
0.550	0.480	0.070	0.088	0.063
0.550	0.480	0.070	0.088	0.063

**Table A220:** Set 3, trials 41–50 data (average actual PR values).

Actual	Average	Average Error	Max Error	Min Error
0.371	0.480	0.109	0.124	0.091
0.437	0.480	0.047	0.063	0.030
0.712	0.603	0.129	0.155	0.105
0.547	0.571	0.047	0.067	0.042
0.432	0.480	0.051	0.067	0.034
0.685	0.603	0.112	0.138	0.086
0.642	0.603	0.086	0.112	0.065
0.561	0.565	0.047	0.067	0.039
0.469	0.480	0.027	0.043	0.020
0.338	0.480	0.142	0.157	0.124

**Table A221:** Set 3, trials 41–50 data (single 0.5 PR value).

Test Avg	Avg Err	Min	Max
0.418	0.005	0.000	0.019
0.471	0.011	0.000	0.037
0.816	0.096	0.081	0.102
0.506	0.010	0.001	0.024
0.497	0.000	0.000	0.000
0.707	0.022	0.009	0.037
0.641	0.023	0.001	0.048
0.545	0.018	0.002	0.038
0.460	0.019	0.019	0.019
0.537	0.013	0.000	0.090

**Table A222:** Set 3, trials 41–50 data (actual PR value).

Test Avg	Avg Err	Min	Max
0.376	0.007	0.011	0.016
0.477	0.038	0.009	0.121
0.816	0.096	0.081	0.102
0.561	0.010	0.001	0.024
0.488	0.057	0.008	0.095
0.707	0.022	0.009	0.037
0.641	0.023	0.001	0.048
0.530	0.019	0.000	0.040
0.454	0.012	0.006	0.027
0.534	0.016	0.000	0.111

**Table A223:** Set 3, trials 41–50 data (all 0.5 PR values).

Test Avg	Avg Err	Min	Max
0.418	0.132	0.127	0.146
0.471	0.079	0.053	0.090
0.816	0.266	0.250	0.272
0.506	0.044	0.030	0.053
0.497	0.053	0.053	0.053
0.707	0.157	0.144	0.172
0.641	0.091	0.067	0.114
0.545	0.005	0.011	0.025
0.460	0.090	0.090	0.090
0.537	0.013	0.000	0.090

**Table A224:** Set 3, trials 41–50 data (average actual PR values).

Test Avg	Avg Err	Min	Max
0.418	0.047	0.034	0.052
0.471	0.034	0.023	0.060
0.816	0.104	0.088	0.110
0.506	0.041	0.027	0.050
0.497	0.065	0.065	0.065
0.707	0.022	0.009	0.037
0.641	0.000	0.022	0.025
0.545	0.017	0.000	0.036
0.460	0.009	0.009	0.009
0.537	0.199	0.122	0.212

**Table A225:** Set 3, trials 41–50 data (single 0.5 PR value).

% Err Avg	% Max Avg	% Min Avg
9.08%	25.07%	0.00%
36.94%	250.00%	0.00%
71.04%	92.81%	49.98%
13.74%	37.74%	0.90%
0.00%	0.00%	0.00%
19.81%	42.95%	6.90%
22.76%	37.88%	0.68%
37.99%	56.12%	4.37%
69.81%	71.43%	45.68%
18.41%	102.10%	0.00%
29.96%	71.61%	10.85%

**Table A226:** Set 3, trials 41–50 data (actual PR value).

% Err Avg	% Max Avg	% Min Avg
5.38%	17.62%	6.56%
55.50%	268.00%	8.17%
71.04%	92.81%	49.98%
21.71%	61.68%	1.23%
77.23%	195.45%	7.25%
19.81%	42.95%	6.90%
22.76%	37.88%	0.68%
40.77%	57.91%	1.20%
24.59%	31.51%	11.51%
20.20%	96.77%	0.00%
35.90%	90.26%	9.35%

**Table A227:** Set 3, trials 41–50 data (all 0.5 PR values).

% Err Avg	% Max Avg	% Min Avg
189.40%	201.43%	165.06%
113.72%	102.10%	84.06%
373.36%	436.85%	261.23%
93.01%	79.21%	72.00%
75.88%	84.06%	60.12%
220.16%	275.95%	150.47%
128.40%	182.62%	69.49%
11.54%	38.25%	29.07%
128.86%	142.74%	102.10%
18.41%	102.10%	0.00%
135.27%	164.53%	99.36%

**Table A228:** Set 3, trials 41–50 data (average actual PR values).

% Err Avg	% Max Avg	% Min Avg
42.80%	57.24%	27.03%
71.41%	203.39%	36.74%
80.13%	103.89%	56.76%
86.39%	74.60%	63.97%
127.42%	194.03%	97.60%
19.81%	42.95%	6.90%
0.17%	33.98%	22.34%
34.99%	54.09%	0.10%
31.58%	42.71%	20.00%
140.09%	171.17%	77.73%
63.48%	97.81%	40.92%

**Table A229:** Set 3, trials 41–50 summary data (actual PR value).

Average Error	Avg Err	Lowest	Correct				False			
			At 0.01	At 0.025	At 0.05	At 0.1	At 0.01	At 0.025	At 0.05	At 0.10
0.125	0.007	Y	Y	Y	Y	Y	0	0	0	3
0.068	0.038	N	N	N	Y	Y	0	1	5	8
0.135	0.096	N	N	N	N	Y	0	1	1	2
0.048	0.010	N	N	Y	Y	Y	1	5	6	8
0.074	0.057	N	N	N	N	Y	0	1	4	6
0.112	0.022	Y	N	Y	Y	Y	0	0	1	3
0.099	0.023	Y	N	Y	Y	Y	0	0	1	3
0.047	0.019	N	N	Y	Y	Y	1	4	6	8
0.049	0.012	N	N	Y	Y	Y	2	5	5	8
0.078	0.016	Y	N	Y	Y	Y	0	1	1	7

**Table A230:** Set 3, trials 41–50 summary data (all 0.5 PR values).

Average Error	Avg Err	Lowest	Correct				False			
			At 0.01	At 0.025	At 0.05	At 0.1	At 0.01	At 0.025	At 0.05	At 0.10
0.070	0.132	N	N	N	N	N	0	1	3	9
0.070	0.079	N	N	N	N	Y	0	1	3	8
0.071	0.266	N	N	N	N	N	3	3	5	8
0.047	0.044	N	N	N	Y	Y	3	5	6	7
0.070	0.053	N	N	N	N	Y	0	1	3	8
0.071	0.157	N	N	N	N	N	3	3	5	8
0.071	0.091	N	N	N	N	Y	3	3	5	7
0.045	0.005	Y	Y	Y	Y	Y	3	3	6	8
0.070	0.090	N	N	N	N	Y	0	1	3	8
0.070	0.013	Y	N	Y	Y	Y	0	0	2	8

**Table A231:** Set 3, trials 41–50 summary data (average actual PR values).

Average Error	Avg Err	Lowest	Correct				False			
			At 0.01	At 0.025	At 0.05	At 0.1	At 0.01	At 0.025	At 0.05	At 0.10
0.109	0.047	Y	N	N	Y	Y	0	0	0	4
0.047	0.034	N	N	N	Y	Y	0	4	5	8
0.129	0.104	N	N	N	N	N	1	1	1	2
0.047	0.041	N	N	N	Y	Y	3	5	6	7
0.051	0.065	N	N	N	N	Y	0	1	6	8
0.112	0.022	Y	N	Y	Y	Y	0	0	1	3
0.086	0.000	Y	Y	Y	Y	Y	0	0	2	6
0.047	0.017	N	N	Y	Y	Y	1	4	5	8
0.027	0.009	N	Y	Y	Y	Y	3	4	7	9
0.142	0.199	N	N	N	N	N	0	0	0	1
